# Updated therapeutic options for human brucellosis: A systematic review and network meta-analysis of randomized controlled trials

**DOI:** 10.1371/journal.pntd.0012405

**Published:** 2024-08-22

**Authors:** Shanjun Huang, Jiaying Xu, Hao Wang, Zhuo Li, Ruifang Song, Yiting Zhang, Menghan Lu, Xin Han, Tian Ma, Yingtong Wang, Jiaxin Hao, Shanshan Song, Qing Zhen, Tiejun Shui

**Affiliations:** 1 Department of Epidemiology and Biostatistics, Key Laboratory of Zoonosis, Ministry of Education, School of Public Health, Jilin University, Changchun, Jilin, PR China; 2 The Second Hospital of Dalian Medical University; 3 State Key Laboratory for Diagnosis and Treatment of Severe Zoonotic Infectious Diseases, Changchun, China; 4 Yunnan Center for Disease Control and Prevention, Kunming, Yunnan, China; George Washington University School of Medicine and Health Sciences, UNITED STATES OF AMERICA

## Abstract

**Background:**

In clinical practice guidelines, there is no consensus about the medications that should be initially offered to patients with brucellosis. To provide informative evidence, we compared and ranked brucellosis medications based on their efficacy and safety.

**Methods:**

For this systematic review and network meta-analysis, we searched 4 English databases and 3 Chinese databases, from the date of database inception to December 13, 2023. We included randomized controlled trials (RCTs) involving children and adolescents with brucellosis, comparing different antibiotic regimens. We excluded studies explicitly targeting patients with spondylitis brucellosis, endocarditis brucellosis, and neuro-brucellosis. The primary outcomes were overall failure (efficacy) and side effects (safety). Secondary outcomes were relapse and therapeutic failure. Pairwise meta-analysis was first examined. Data were analyzed using random effects network meta-analysis, with subgroup and sensitivity analyses performed. The Confidence in Network Meta-Analysis (CINeMA) framework was used to assess the certainty of evidence. The protocol was preregistered in PROSPERO (CRD42023491331).

**Results:**

Of the 11,747 records identified through the database search, 43 RCTs were included in the network meta-analysis. Compared with standard therapy (Doxycycline + Rifampicin), Rifampicin + Tetracyclines (RR 4.96; 95% CI 1.47 to 16.70; very low certainty of evidence), Doxycycline + TMP/SMX (RR 0.18; 95% CI 0.06 to 0.52; low certainty of evidence), Doxycycline + Quinolones (RR 0.27; 95% CI 0.11 to 0.71; low certainty of evidence), Streptomycin + Tetracyclines (RR 0.04; 95% CI 0.01 to 0.16; low certainty of evidence), and Single (RR 0.05; 95% CI 0.02 to 0.16; moderate certainty of evidence) were less efficacious. Doxycycline + Gentamicin ranked the best in efficacy (SUCRA values: 0.94), the second is Triple (SUCRA values: 0.87), and the third is Doxycycline + Streptomycin (SUCRA values: 0.78).

**Conclusions:**

Brucellosis medications differ in efficacy and safety. Doxycycline + Gentamicin, Triple, and Doxycycline + Streptomycin have superior efficacy and safety. Treatment of brucellosis should strike a balance between efficacy, safety, and cost.

## Author summary

Brucellosis, with nearly 500,000 new cases outbreak in more than 170 countries or regions worldwide each year, is a zoonotic infectious disease caused by *Brucella*. Due to the lack of an effective human vaccine and the impracticality of eradicating brucellosis in animals globally, treatment remains the primary strategy for addressing human brucellosis. However, there is no consensus in clinical practice guidelines regarding the initial medications for brucellosis treatment. Therefore, we compared and ranked brucellosis medications based on their efficacy and safety to aid clinicians in matching risk/benefits of drugs to brucellosis treatment. In this systematic review and network meta-analysis of 43 randomized clinical trials with 4,283 patients, 6 weeks of doxycycline plus 1 to 2 weeks of gentamicin or plus 2 to 3 weeks of streptomycin is the best therapy for human brucellosis. Six weeks of rifampicin plus quinolones treatment is considered as an alternative therapy. If finances allow, consider triple therapy. Single, Streptomycin + Tetracyclines, Streptomycin + TMP/SMX, and Rifampicin + TMP/SMX therapies cannot currently be recommended.

## Introduction

Brucellosis is a zoonotic infection caused by the gram-negative, intracellular parasitic bacterium, and is listed as one of the “seven neglected endemic zoonoses,” according to the World Health Organization (WHO) [1]. During the COVID-19 pandemic, the incidence of brucellosis showed a significant increase [2]. Economic losses from human brucellosis are significant, including medical costs for treatment and income loss from missed workdays [3].

The initial treatment failure and recurrence of brucellosis are common, varying from 5% to 15% in mild cases [4]. Moreover, due to the nonspecific clinical symptoms of brucellosis, misdiagnosis often leads to disease progression into the chronic phase [5]. Although rarely fatal, complications arising at this stage can result in disability, skeletal deformities, and, in severe cases, death [6,7]. The presence of relapses, chronicity, sequelae, and the potential for mortality underscore the seriousness of brucellosis as a threat, emphasizing the imperative to implement appropriate measures for its treatment and control [8]. However, from the initially adopted monotherapy to the 21-day course of treatment with tetracycline and streptomycin recommended by WHO in 1971 [9], the results have been unsatisfactory because of high relapses after apparently successful treatment [10]. In 1986, WHO recommended the combination of doxycycline-rifampicin as the treatment of choice for human brucellosis, and it is still used today [11]. Nevertheless, subsequent research revealed that in practice, the combination therapy of doxycycline and rifampicin was not as effective as the traditional treatment involving tetracycline and streptomycin [12,13]. Furthermore, with the emergence of multidrug-resistant strains of *Brucella*, treating brucellosis increasingly requires regimens that do not include rifampicin (commonly used in the treatment of tuberculosis) [14,15]. Later studies found that combination therapy with aminoglycosides or triple therapy has shown promising results [15,16]. Yet, there are still many challenges to overcome, such as the need for parenteral administration of aminoglycosides and the economic burden of triple therapy.

There is inconsistency in treatment recommendations due to the lack of large-scale, double-blind randomized controlled trials (RCTs) on pharmacological interventions for brucellosis. Network meta-analyses can compare the effects of multiple interventions even without direct RCTs, providing high-level evidence for treatment guidelines [17]. However, no such analyses exist for brucellosis treatments.

To fill this gap, our objective is to establish an evidence-based hierarchy of the comparative efficacy and safety of medications for treating brucellosis, thereby assisting clinicians, patients, and policymakers in making informed decisions regarding the most suitable treatments for human brucellosis.

## Methods

### Protocol and registration

The results of the systematic review and network meta-analysis followed the Preferred Reporting Items for Systematic Reviews and Meta-Analyses (PRISMA) extension statement for reporting of systematic reviews incorporating network meta-analyses [18]. See **S1 Table** for the PRISMA-NMA checklist. The study protocol was preregistered in PROSPERO (CRD42023491331). Revisions to the predetermined protocol, accompanied by justifications, are listed in **S2 Table**.

### Search strategy and selection criteria

We searched 4 English databases (PubMed, Embase, Web of Science, and the Cochrane Central Register of Controlled Trials) and 3 Chinese databases (China National Knowledge Infrastructure (CNKI), SinoMed, and Wanfang Data), from the date of database inception to December 13, 2023. We combined Medical Subject Headings (MeSH) and Entry Terms to develop a search strategy. Searched characters using the following keywords: “Brucellosis,” “Brucella,” “Malta Fever,” “Gibraltar Fever,” etc. In addition, we adopted a highly sensitive search strategy for RCTs provided by the Cochrane Library [19]. See **S3 Table** for search strategies of relevant databases. At the same time, we also reviewed researches from the ClinicalTrials.gov and hand-searched the references in the included studies to ensure the inclusion of all relevant literature. Finally, we compared previous systematic reviews and meta-analyses on related topics to ensure that we did not miss literature.

In this systematic review and network meta-analysis, we included RCTs that enrolled children and adolescents, or both, with a primary diagnosis of brucellosis according to the clinical symptoms, epidemiological history, and laboratory tests. We included double-arm or multi-arm RCTs that tested any single or combination antibiotic treatment for *Brucella* infections compared with another antibiotic regimen (different drugs or different duration of treatment). To avoid violation of the transitivity assumption [20], we excluded studies specifically targeting patients with spondylitis brucellosis, endocarditis brucellosis, and neuro-brucellosis. We also excluded studies that focused on pregnant women.

### Study selection and data extraction

After training and calibration exercises to ensure sufficient agreement, 2 reviewers (HSJ and XJY) screened the search results and extracted data independently, in case of doubt discussion with a third reviewer (ZQ) resolved the issue. Data extracted included trial characteristics (i.e., author, year published, interventions, and country of enrolment), patient characteristics (i.e., age, sex, sample size, inclusion and exclusion criteria, time to defervescence, and follow-up time), and our outcomes of interest. Our 2 predefined primary outcomes were overall failure, defined as the sum of relapse and therapeutic failure, and side effects, defined as uncomfortable symptoms that occur during the administration of the drug, such as nausea and vomiting, abdominal pain and diarrhea, etc., serious side effects such as ototoxicity, hepatotoxicity, nephrotoxicity, and skin reactions. Secondary outcomes included relapse, defined as the reappearance of relevant clinical symptoms, rise in antibody titers or positive results on cultures after the end of treatment, during the follow-up period, and therapeutic failure, defined as the patient’s symptoms not improving by the end of treatment [21,22]. Based on the above definition, if a study reported only the events of relapse or treatment failure, we treated either as overall failure. In addition, we used overall failure to measure the efficacy of the treatment regimen and side effects to measure the safety of the treatment regimen. The selection of the outcome endpoint was based on the outcome endpoint reported in original studies. For the outcome data of different follow-up periods, we selected only the one with the longest follow-up as the final data for extraction. Extraction of data based on the principles of intention-to-treat analysis is preferred, but we also extracted data from participants who completed the study (modified intention-to-treat analyses) if that was the only analysis reported. Finally, for studies published more than once (i.e., duplicates), we included only the report with the most informative and complete data.

### Statistical analysis

We selected the WHO-recommended standard treatment as the control due to its strong connection with other network interventions [23]. We conducted a pairwise meta-analysis using a random effects model, assessing heterogeneity via the I^2^ statistic and 95% CIs. A network meta-analysis followed, incorporating class and individual data in a frequentist framework, computing RR and 95% CIs. We assessed local and global inconsistency using the loop-specific approach and the design-by-treatment test and visualized the intervention network. The surface under the cumulative ranking curve (SUCRA) value indicated intervention possibilities. To identify heterogeneity and inconsistency sources, we performed subgroup and sensitivity analyses. We evaluated the risk of bias of RCTs with the Cochrane tool [24] and assessed confidence in primary outcome estimates using the Confidence In Network Meta-Analysis (CINeMA) framework. Small-study effects were analyzed using a comparison-adjusted funnel plot and Egger’s test [25]. Analyses were conducted in R 4.3.1 and Stata 17.0. Detailed descriptions of data analysis can be found in **S4 Table**.

## Results

### Study selections and quality assessment

**Fig 1:**
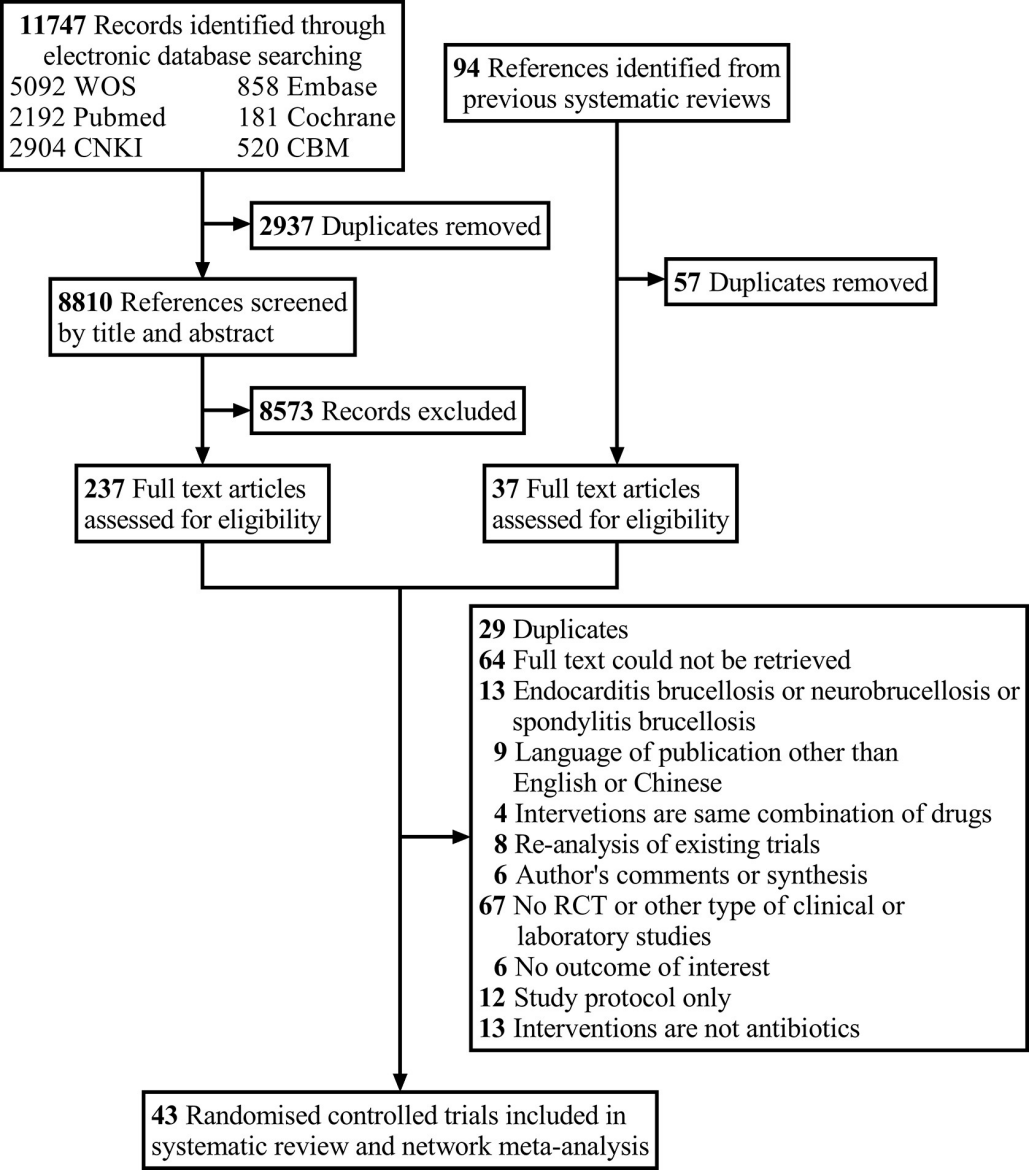
Flowchart of study selection.

We initially retrieved 11,747 records through the database search and screened 8,810 records after excluding duplicates. After screening based on the title and abstract, the remaining 237 articles may meet the criteria. With the addition of 37 articles extracted from previous systematic reviews, a total of 274 records were reviewed. Ultimately, 43 RCTs were included in our systematic review and network meta-analysis (**S5 Table**). Study selection is reported in **Fig 1**. One of the papers incorporated 2 distinct studies conducted in separate phases. However, as the results of the first phase of the study had already been published previously, only the latter phase was included in the network analysis [26]. Three studies could not be found, so their data were derived from 2 previous systematic reviews and meta-analyses [21,27–30]. The characteristics of retained studies are provided in **Tables 1** and **S6,** and **S7 Table** presents more details on the excluded studies.

**Table 1:** Characteristics of included studies.

Trial	Interventions	Country	Duration	Patients	Mean age	Man%	Follow-up time (months)
Acocella 1989[w1]	D 1×200mg for 45 days + R 1×900mg for 45 days vs D 1×200mg for 45 days + S 1×1g for 21 days vs TC 4×0.5g for 21 days + S 1×1g for 14 days	France, Greece, Spain	May 1981 to July 1984	146	41 (13-70)	65.7	12
Agalar 1999[w2]	D 2×100mg for 45 days + R 1×600mg for 45 days vs R 1×600mg for 30 days + CIP 2×500mg for 30 days	Turkey	October 1995 to January 1998	40	37.5 ± 14.7	52.5	12
Akova 1993[w3]	D 1×200mg for 6 weeks + R 1×600mg for 6 weeks vs O 1×400mg for 6 weeks + R 1×600mg for 6 weeks	Turkey	March 1989 to March 1992	61 (6)^†^	36.4 ± 14.7	49.2	14.7 ± 5.7
Alavi 2007[w4]	D 2×100mg for 8 weeks + R 3×300mg for 8 weeks vs D 2×100mg for 8 weeks + TMP/SMX 2×960mg for 8 weeks	Iran	April 2004 to January 2006	105	30.6 ± 10.5	NR	6
Ariza 1992[w5]	D 2×100mg for 45 days + R 15mg/kg/day for 45 days vs D 2×100mg for 45 days + S 1×1g for 15 days	Spain	1986–9	95 (7)^†^	39.1 ± 16.5	71.6	15.7 ± 6.3
Buzon 1982[w6]	TC 4×0.5g for 4 weeks + R 1200mg/day for 1 week, followed by 600mg/day for 3 weeks vs C 480/2400mg/day for 10 days and 320/1600mg/day for 20 days, followed by 160/400mg/day for 6 months	Spain	NR	84	NR	NR	6
Chai 2018[w7]	D 2×100mg for 6 weeks + L 1×500mg for 6 weeks vs L 1×500mg for 6 weeks	China	March 2014 to March 2017	78	40.1 ± 9.0	70.5	NR
Chen 2016[w8]	D 2×100mg for 6 weeks + L 1×500mg for 6 weeks vs CIP 2×250mg for 6 weeks	China	January 2014 to October 2015	65	42 (23–68)	63.1	NR
Colmenero 1989[w9]	D 2×100mg for 45 days (60 days) + R 15mg/kg/day for 45 days (60 days) vs D 2×100mg for 30 days (60 days) + S 1×1g for 21 days	Spain	1985–6	111	33.1 ± 4.1	69.4	NR
Colmenero 1994[w10]	D 2×100mg for 6 weeks (12 weeks) + R 10–15mg/kg/day for 6 weeks (12 weeks) vs D 2×100mg for 6 weeks (12weeks) + S 1×1g for 3 weeks	Spain	NR	19 (2)^†^	33.3 ± 15.6	65	NR
Deng 2016[w11]	D + R 3–4 tablets/day vs D 1–2 tablet/day (doses not available)	Chian	September 2013 to September 2015	64	52.7 ± 5.9	68.8	NR
Ersoy 2005[w12]	D 200mg/day for 6 weeks + R 600mg/day for 6 weeks vs D 100mg/day for 6 weeks + S 1g/day for 3 weeks vs O 400mg/day for 6 weeks	Turkey	May 1997 to December 2002	118	36.4 ± 2.4	52.5	At least 6
Guo 2023[w13]	D 2×100mg for 6 weeks + L 1×500mg for 6 weeks vs L 1×500mg for 6 weeks	China	March 2021 to March 2022	84	41.0 ± 5.1	100	NR
Hasanain 2016[w14]	D 1×200mg for 6 weeks + R 1×900mg for 6 weeks vs D 1×200mg for 6 weeks + R 1×900mg for 6 weeks + L 1×500mg for 6 weeks	Egypt	May 2011 to November 2014	120	34.5 ± 16.3	57.9	6
Hasanjani Roushan 2006[w15]	D 2×100mg for 45 days + S 1×1g for 14 days vs D 2×100mg for 45 days + G 5mg/kg/day for 7 days	Iran	October 2003 to June 2005	200	35.0 ± 15.4	57.1	12
Hashemi 2011[w16]	D 1×200mg for 6 weeks + R 15mg/kg/day for 6 weeks vs D 1×200mg for 6 weeks + S 1×1g for 3 weeks vs O 1×800mg for 6 weeks	Iran	April 2008 to March 2010	219	39.8 ± 15.4	55.5	6
Hassan 2022[w17]	D 2×100mg for 6 weeks + R 600mg/day for 6 weeks vs D 2×100mg for 6 weeks + TMP/SMX 2×160/800mg for 6 weeks	Egypt	June 2020 to January 2022	100	38.6 ± 12	71	6
Hassanjani Roushan 2010[w18]	D 2×100mg for 45 days + S 1×1g for 2 weeks vs D 2×100mg for 8 weeks + G 5mg/kg/day for 5 days	Iran	April 2005 to September 2008	164 (3)^†^	36.2±14.6	65.9	12
Jiang 2020[w19]	D 2×100mg for 6 weeks + R 1×600mg for 6 weeks + L 2×200mg for 7 days vs D 2×100mg for 6 weeks + R 1×600mg for 6 weeks	China	May 2016 to January 2018	74	39.1 ± 16.6	56.8	6
Ju 2022[w20]	R 1×600mg for 20 days + TC 2×100mg 20 days vs R 1×600mg for 20 days	China	January 2021 to November 2021	116	NR	NR	NR
Kalo 1996[w21]	D 200mg/day for 6 weeks + R 900mg/day for 6 weeks vs D 200mg/day for 6 weeks + CIP 1g/day for 6 weeks	Albania	January 1992 to December 1994	24	31.8 ± 13.5 (18–56)	58.3	6
Karabay 2004[w22]	D 1×200mg for 45 days + R 1×600mg for 45 days vs R 1×600mg for 30 days + O 1×400mg for 30 days	Turkey	December 1999 and December 2001	34	32.1 (18–61)	82.8	5.1 ± 0.6
Keramat 2009[w23]	D 1×200mg for 8 weeks (12weeks) + R 15mg/kg/day (600-900mg) for 8 weeks (12weeks) vs R 15mg/kg/day for 8 weeks (12weeks) + CIP 2×15mg/kg/day (500-750mg) for 8 weeks (12weeks) vs D 1×200mg for 8 weeks (12weeks) + CIP 2×15mg/kg/day (500-750mg) for 8 weeks (12weeks)	Iran	April 2002 to December 2006	178	40.7	58.9	6
Lang 1990[w24]	D 2×100mg for 6 weeks + R 2×300mg for 6 weeks vs CIP 2×1g for 6 weeks vs CIP 2×750mg for 6 weeks	Israel	NR	10	38.4 ± 12.2	50	NR
Lang 1992[w25]	D 1×100mg for 4 weeks + S 20mg/kg/day for 14 days vs CEF 75mg/kg/day for at least 2 weeks	Israel	1989	18	28.4 ± 13.5	27.8	6
Liu 2018[w26]	D 2×100mg for 12 weeks + R 2×450mg for 12 weeks vs D 2×100mg for 12 weeks	China	June 2015 to July 2017	72	46.8 ± 6.8	62.5	NR
Liu 2019[w27]	D 2×100mg for 12 weeks + R 1×600-900mg for 6 weeks + TMP/SMX 1×500mg for 12 weeks vs D 2×100mg for 12 weeks + R 1×600-900mg for 6 weeks	China	February 2016 to May 2018	114	33.7 ± 2.1	52.3	NR
Montejo 1993b[w28]	D 1×200mg for 6 weeks + S 1×1g for 3 weeks vs D 1×200mg for 6 weeks + R 1×900mg for 6weeks vs S 1×1g for 2 weeks	Spain	1984–7	130	46 (14–82)	74	NR
Qian 2008[w29]	D 1×200mg for 6 weeks + L 1×500mg for 6 weeks vs S 1×750mg for 3 weeks + TMP/SMX 2×2 tablets for 6 weeks	China	NR	105	(18-68)	72.4	NR
Qian 2009[w30]	D 1×200mg for 6 weeks + L 1×500mg for 6 weeks vs S 1×750mg for 3 weeks + TET 4×500mg for 6 weeks	China	July 2004 to July 2007	84	39.5 ± 3	69	NR
Ranjbar 2007[w31]	D 2×100mg for 8 weeks + R 10mg/kg/day for 8 weeks vs D 2×100mg for 8 weeks + R 10mg/kg/day for 8 weeks + A 2×7.5mg/kg for 7 days	Iran	1999-2001	228	36.4±17.7	48.6	6
Roushan 2004[w32]	D 2×100mg for 8 weeks + TMP/SMX 8 mg/kg/day for 8 weeks vs R 15mg/kg/day for 8 weeks + TMP/SMX 8mg/kg/day for 8 weeks	Iran	April 1999 to January 2002	280	33.5 ± 17.2	53.6	NR
Sarmadian 2009[w33]	D 2×100mg for 8 weeks + R 10mg/kg/day for 8 weeks vs D 2×100mg for 8 weeks + CIP 2×500mg for 8 weeks	Iran	2006-8	80	NR	NR	NR
Sha 2017[w34]	D 2×100mg for 6 weeks + R 1×600mg for 6 weeks + L 1×500mg for 6 weeks vs D 2×100mg for 6 weeks + R 1×600mg for 6 weeks	China	November 2015 to March 2017	112	46 ± 7.1	77.7	6 weeks
Sun 2015[w35]	R 1×750mg vs D 1×200mg + R 1×750mg + L 1×300mg for 7 days	China	2013-5	48	52.7 ± 6.0	73.8	NR
Sun 2020[w36]	D 2×100mg for 6 weeks + R 1×600mg for 6 weeks + L 1×500mg for 6 weeks vs D 2×100mg for 6 weeks + R 1×600mg for 6 weeks	China	January 2018 to June 2020	60	45.7 ± 3.0	53.3	3
Sun 2023[w37]	D 2×100mg for 6 weeks + R 1×600mg for 6 weeks vs R 1×600mg for 6 weeks + M 2×100mg for 6 weeks	China	January 2018 to December to 2021	60	42.2 ± 10.9	68.3	6 weeks
Wang 2020[w38]	D 2×100mg for 6 weeks + L 2×500mg for 6 weeks vs L 2×500mg	China	NR	50	44.7 ± 5.3	58	NR
Wang 2022[w39]	D 2×100mg for 6 weeks + L 1×500mg for 6 weeks vs L 1×500mg for 6 weeks	China	July 2020 to July 2021	100	44.6 ± 0.7	45	NR
Yin 2015[w40]	D 2×100mg for 6 weeks + R 1×600mg for 6 weeks + L 1×400mg for 7 days vs D 2×100mg for 6 weeks + R 1×600mg for 6 weeks	China	January 2010 to December 2012	64	42 ± 18.6	68.8	6
Zhang 2022[w41]	D 2×100mg for 6 weeks + R 1×900mg for 6 weeks + L 1×500mg for 6 weeks vs D 2×100mg for 6 weeks + R 1×900mg for 6 weeks	China	March 2020 to March 2021	76	49.5 ± 3.1	51.3	2
Zhao 2023[w42]	R 1×600mg for 4 weeks + TC 2×100mg for 4 weeks + L 1×500mg for 4 weeks vs R 1×600mg for 4 weeks + TC 2×100mg	China	December 2020 to October 2022	127	41.9 ± 6.3	66.1	2
Zhou 2016[w43]	D 2×100mg for 6 weeks + R 1×600mg for 6 weeks + L 1×500mg for 6 weeks vs D 2×100mg for 6 weeks + R 1×600mg for 6 weeks	China	November 2014 to June 2015	120	47.8 ± 11.5	78.3	6

D = doxycycline, R = rifampicin, S = streptomycin, O = ofloxacin, TMP/SMX = cotrimoxazole, G = gentamicin, CEF = ceftriaxone, CIP = ciprofloxacin, TET = tetracycline, L = levofloxacin, A = amikacin, TC = tetracycline hydrochloride, OTC = oxytetracycline, M = minocycline. NR = not report.

^†^Number of spondylitis brucellosis in parentheses.

A detailed description of the methods to assess the risk of bias and certainty of evidence is provided in **S8 Table**, and **S9 Table** presents our risk of bias assessments for the studies. For overall failure outcome, 18 studies were rated as having a high risk of bias, 13 had a moderate risk, and 13 had a low risk; for side effects outcome, 8 studies were rated as having a high risk of bias, 10 had a moderate risk, and 19 had a low risk. Domain-level risk of bias assessments for each drug comparison are provided in **S10 Table**.

### Study characteristics

Of the 43 studies included in our research, 4,283 participants were randomly assigned to different pharmacological interventions. Age ranged from 13 to 70 years for all patients with the proportion of male is 68.5%. All studies were conducted in developing countries. Approximately 58.1% of studies reported follow-up times, with the longest reaching 24 months and the shortest 2 weeks. Fifteen medications were classified into 12 combinations: Doxycycline + Rifampicin (28 trials), Doxycycline + Streptomycin (10 trials), Doxycycline + Gentamicin (2 trials), Doxycycline + Quinolones (including ofloxacin, levofloxacin, and ciprofloxacin; 10 trials), Rifampicin + Quinolones (6 trials), Rifampicin + Tetracyclines (including tetracycline, tetracycline hydrochloride, oxytetracycline, minocycline; 4 trials), Streptomycin + Tetracyclines (2 trials), Doxycycline + TMP/SMX (cotrimoxazole; 3 trials), Rifampicin + TMP/SMX (1 trial), S+TMP/SMX (1 trial), single (any single drug treatment; 12 trials), and triple (3 antibiotics were used in combination; 11 trials, 8 of which were Doxycycline + Rifampicin + Levofloxacin).

### Pairwise meta-analyses results

**Fig 2:**
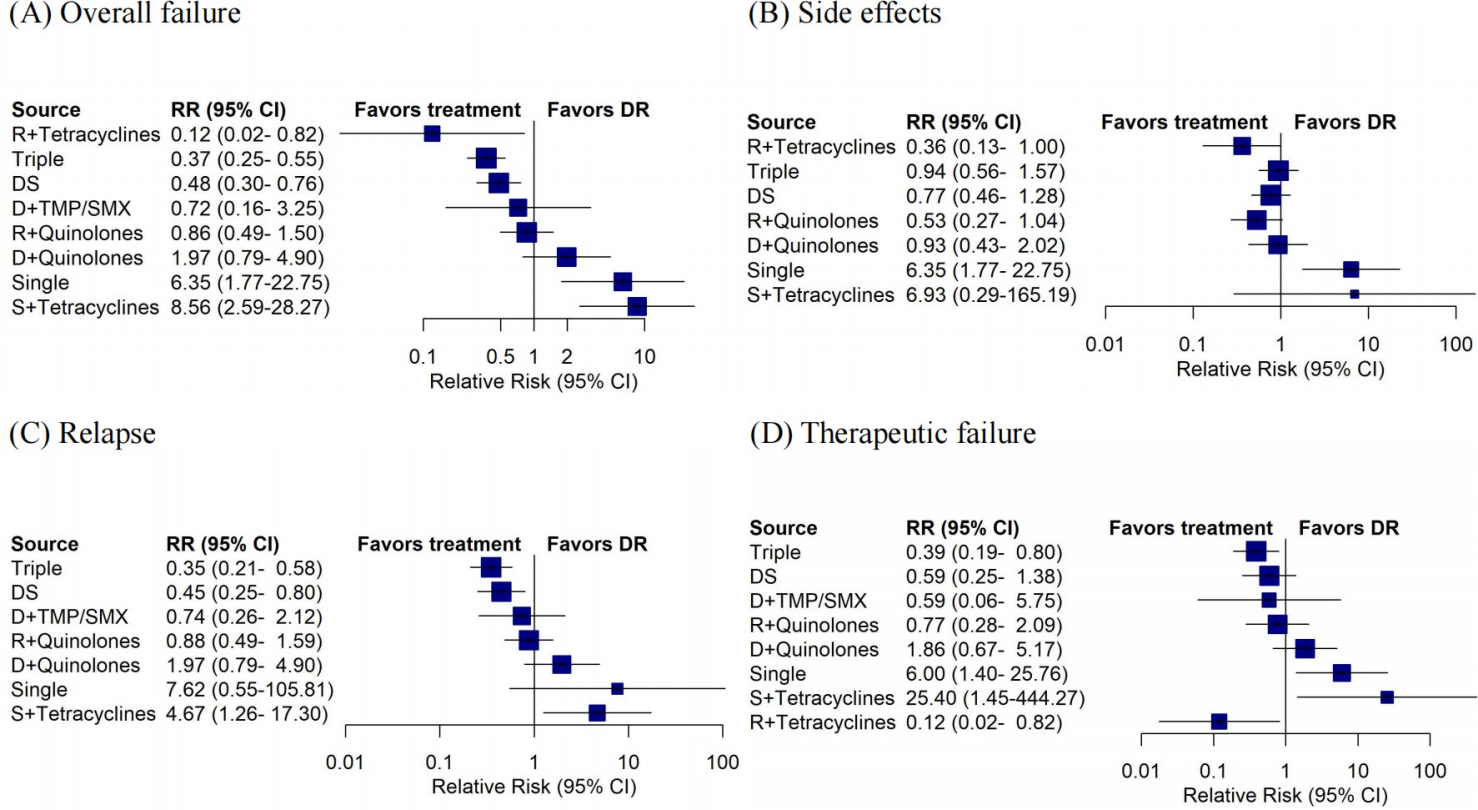
Forest plot for pairwise comparison of other treatments against DR. (**A**) The forest plot of overall failure outcome. (**B**) The forest plot of side effects outcome. (**C**) The forest plot of relapse outcome. (**D**) The forest plot of therapeutic failure outcome.

**Fig 2** displays a forest plot comparing various drug combinations to the standard therapy (doxycycline plus rifampicin), as recommended by WHO. For overall failure outcome, Triple (RR 0.37; 95% CI 0.25 to 0.55) and Doxycycline + Streptomycin (RR 0.48; 95% CI 0.30 to 0.76) were more efficacious than Doxycycline + Rifampicin; Doxycycline + Rifampicin was better than Single and Streptomycin + Tetracyclines, with wider confidence intervals (**Fig 2A**). However, there were no statistically significant differences in the safety of any of other drug therapies compared to Doxycycline + Rifampicin, except for Single treatment (**Fig 2B**). For secondary outcome indicators, the risk of relapse was lower for Triple (RR 0.35; 95% CI 0.21 to 0.58) and Doxycycline + Streptomycin (RR 0.45; 95% CI 0.25 to 0.80) than for standard therapies (**Fig 2C**); only Triple (RR 0.39; 95% CI 0.19 to 0.80) and Rifampicin + Tetracyclines (RR 0.12; 95% CI 0.02 to 0.82) had lower treatment failure than the Doxycycline + Rifampicin (**Fig 2D**). The sources of the studies and the sample sizes for each therapy comparison are shown in **S1 Fig**.

### Network meta-analyses results

**Fig 3:**
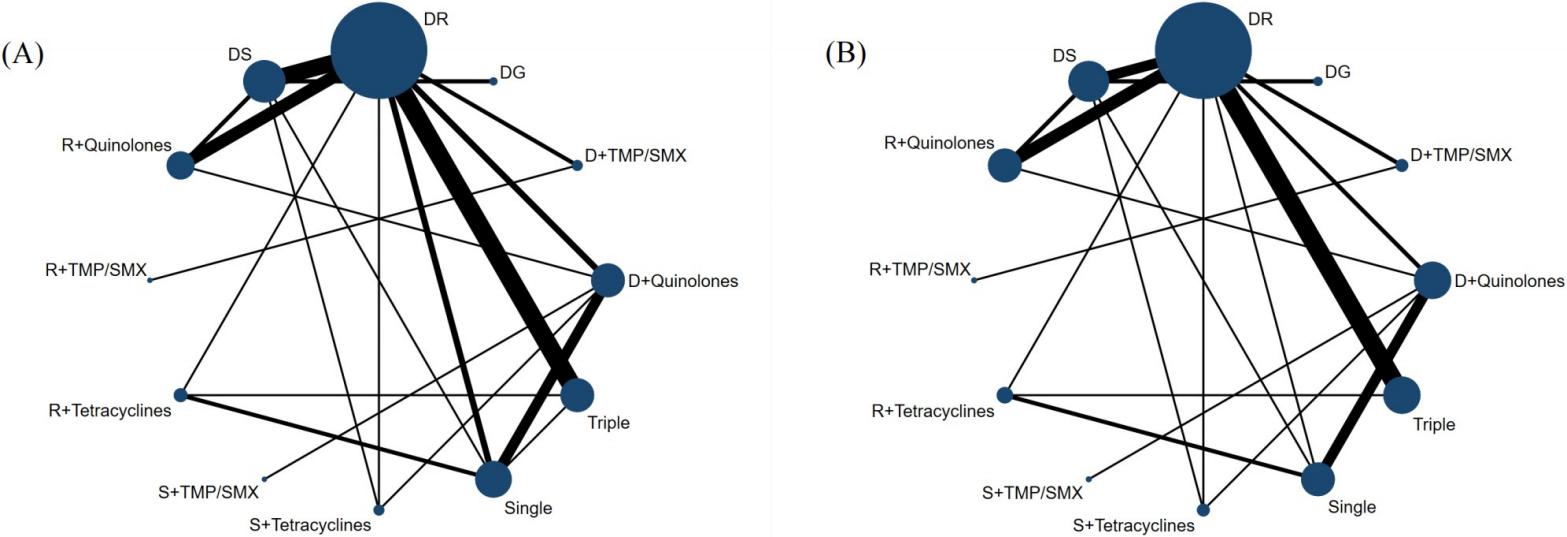
Network plots for primary outcomes. (**A**) The network plot of overall failure. (**B**) The network plot of side effects. The size of the sphere is proportional to the number of patients that have received that drug treatment, and the thickness of the connecting line is proportional to the number of trials.

**Fig 3** shows the network geometry for each primary outcome. There were lots of head-to-head comparisons, and the networks were well connected. It can be seen from the thickness of the line that most comparisons were based on Doxycycline + Rifampicin. **S2 Fig** shows the network plots for secondary outcomes.

**Fig 4:**
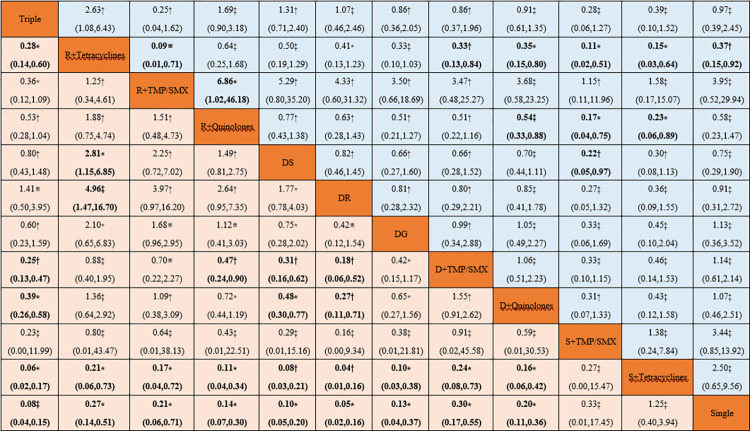
League table of efficacy and safety of pharmacological interventions in network meta-analysis. Efficacy (pale orange boxes) and safety (pale blue boxes) are reported as relative risk with 95% CIs. For efficacy and safety, relative risk below 1 favor the column-defining treatment. ※High certainty of evidence. *Moderate certainty of evidence. †Low certainty of evidence. ‡Very low certainty of evidence.

Most drug therapies were superior in efficacy compared to Single treatment and did not increase the incidence of side effects. Rifampicin + Tetracyclines had both better efficacy (RR 0.27; 95% CI 0.14 to 0.51; moderate certainty of evidence) and safety (RR 0.37; 95% CI 0.15 to 0.92; low certainty of evidence) than monotherapy. Compared with standard therapy, Rifampicin + Tetracyclines (RR 4.96; 95% CI 1.47 to 16.70; very low certainty of evidence), Doxycycline + TMP/SMX (RR 0.18; 95% CI 0.06 to 0.52; low certainty of evidence), Doxycycline + Quinolones (RR 0.27; 95% CI 0.11 to 0.71; low certainty of evidence), Streptomycin + Tetracyclines (RR 0.04; 95% CI 0.01 to 0.16; low certainty of evidence), and Single (RR 0.05; 95% CI 0.02 to 0.16; moderate certainty of evidence) were less efficacious. When considering medication categories, both combining rifampicin with tetracyclines (RR 0.09; 95% CI 0.01 to 0.71; high certainty of evidence) and pairing it with quinolones (RR 0.15; 95% CI 0.02 to 0.98; moderate certainty of evidence) offer a safer profile compared to rifampicin combined with TMP/SMX. For tetracyclines combined with rifampicin (RR 0.21; 95% CI 0.06 to 0.73; moderate certainty of evidence) or combined with streptomycin (RR 0.15; 95% CI 0.03 to 0.64; moderate certainty of evidence), the former is superior in terms of efficacy and safety. Aminoglycosides, in combination with doxycycline (Doxycycline + Streptomycin and Doxycycline + Gentamicin), has better efficacy than most therapies (**Fig 4**). The league table of relapse and therapeutic failure is shown in **S11 Table**.

**Fig 5:**
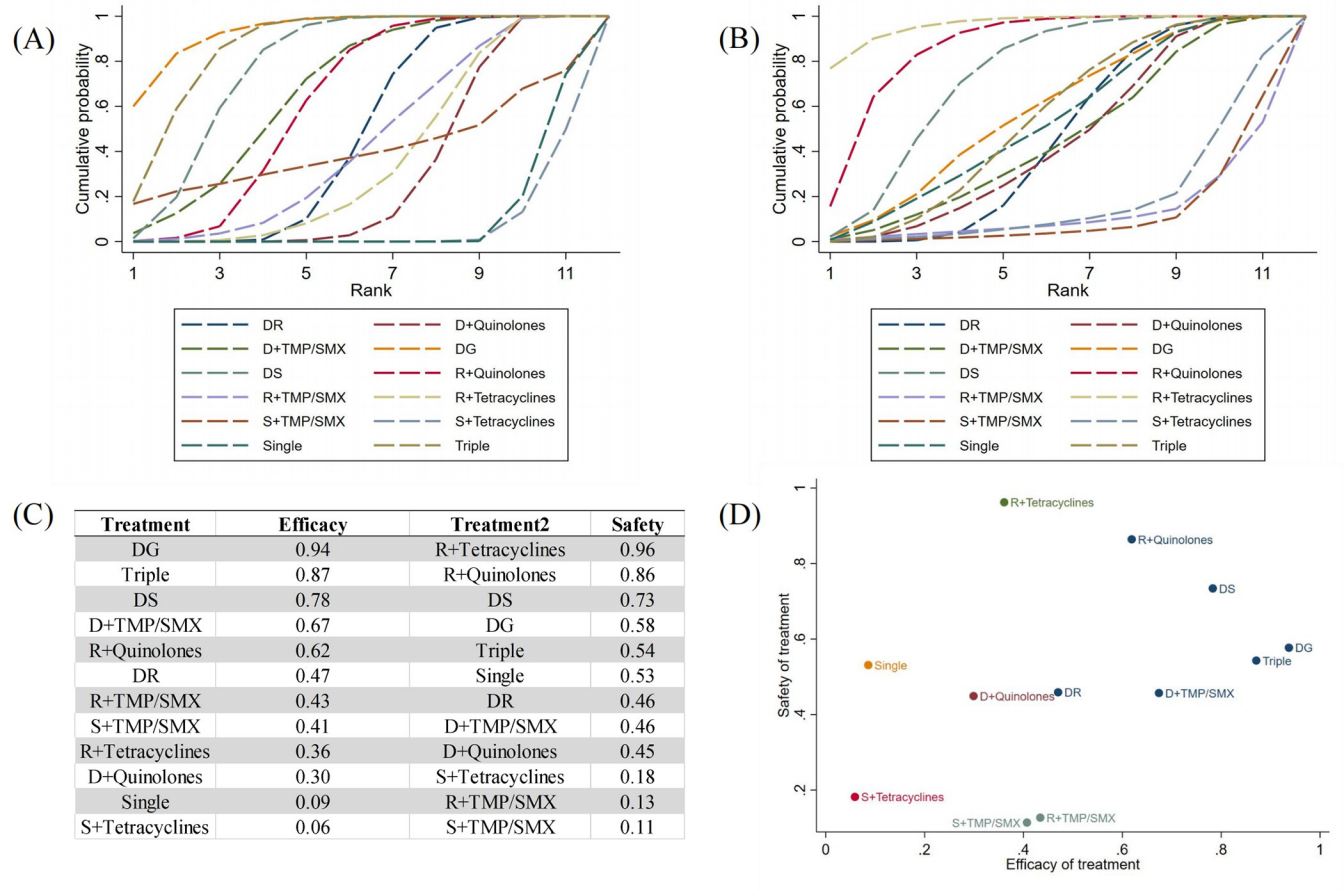
Plot of efficacy and safety of therapies based on SUCRA values. (**A**) Cumulative probability plots of drug efficacy rankings. (**B**) Cumulative probability plots of drug safety rankings. (**C**) SUCRA values of each drug treatment. (**D**) Cluster plot of efficacy and safety of all drug treatments. The higher the value, the better the efficacy or safety.

The ranking probability was further assessed based on SUCRA. The graphical presentation and SUCRA values of efficacy and safety outcomes of 12 different drug treatments are shown in **Fig 5A–5C.** From top to bottom, Doxycycline + Gentamicin demonstrates the best efficacy (0.94), second for Triple (0.87), and third for DS (0.78). Rifampicin + Tetracyclines was highest for safety (0.96), followed by Rifampicin + Quinolones (0.86). To more visually present the combined effects of efficacy and safety of drug therapy, we integrated drug efficacy and safety (**Fig 5D**). It could be seen that compared to the other therapies, Streptomycin + Tetracyclines and Single were at the far left end of the x-axis, where the values were lowest, indicating that their efficacy was the worst. Streptomycin + TMP/SMX and Rifampicin + TMP/SMX were at the minimum value of the y-axis, indicating that they had the lowest security.

There was no evidence of heterogeneity in any of the networks (**S12 Table**). There was evidence of some inconsistency in the network meta-analysis for overall failure. Although the global inconsistency test was nonsignificant (*p* = 0.1066), 2 of 13 loops showed inconsistency. With the Node-splitting approach, we detected these 2 comparisons as Doxycycline + Rifampicin versus Rifampicin + Tetracyclines (*p* = 0.012) and Triple versus Single (*p* = 0.037), respectively. There was no evidence of inconsistency for the remaining network meta-analyses (**S13 Table**). The findings did not change considerably in sensitivity analyses (**S3 Fig**). Subgroup analyses found that the significance of better efficacy of Doxycycline + Gentamicin and Doxycycline + Streptomycin than Doxycycline + Rifampicin came mainly from studies in non-Chinese regions, whereas the finding that triple therapy possessed better efficacy came mainly from the Chinese region (**S14 Table**). Finally, there was no evidence of publication bias for any of the network meta-analyses (**S4 Fig**).

## Discussion

The evidence to date has demonstrated that: The overall treatment failure and relapse rates of doxycycline plus rifampicin are higher than those of doxycycline plus streptomycin; gentamicin and streptomycin have comparable efficacy; monotherapy carries a higher risk of failure compared to combination therapy when administered for the same duration; treatment for 6 weeks or longer is superior to shorter treatment durations; triple therapy generally outperforms dual therapy [21,22,29,31,32]. Our systematic review and network meta-analysis not only updates the relevant evidence but also has broader conclusions.

We found that Doxycycline + Gentamicin is the best in terms of efficacy, which was also recommended in a previous systematic review [15,21]. The second-ranked treatment is triple therapy. Most of the comparisons for this drug come from the same area. A previous review, which included RCTs and cohort studies, confirmed that triple therapy is more efficacious than dual therapy and does not increase the incidence of side effects [22]. It is the same as ours. As for tetracycline, our results showed that Rifampicin + Tetracyclines have the highest safety profile. The quality assessment for studies that include Tetracyclines regimes was rated as “some concerns, 3 trials” and “low risk, 1 trial,” respectively. Limited by the quality of the literature and the likelihood of tetracyclines causing tetracycline pigmentation of teeth, and some studies did not perform any explicit assessment of minor adverse reactions, we believe that this conclusion needs to be interpreted with caution. Our research shows that Rifampicin + Quinolones is also highly safe. The study suggested that the combination of rifampicin and quinolones may be an alternative to doxycycline plus rifampicin [31]. However, Skalsky and colleagues [21] have recommended against the use of quinolones-containing regimens. Nonetheless, the results of this meta-analysis were influenced by an original study conducted only in patients with osteoarticular brucellosis [33]. We believe that this may underestimate the treatment effect, both in terms of safety and effectiveness. By extrapolating the results of this study to nonfocal brucellosis seems premature. Quinolones demonstrate robust effectiveness in vitro against *Brucella* species. The minimal inhibitory concentration for 90% growth (MIC90) of ofloxacin against *Brucella melitensis* is reported to be as low as 0.02 mg/L [34], while another study indicates that ciprofloxacin was effective at only 0.5 mg/L [35]. Perhaps the Rifampicin + Quinolones regimen could be considered an option for the treatment of human brucellosis.

It is worth noting that the need for parenteral administration of aminoglycosides and the increased financial burden of triple therapy may complicate the use of these regimens. A study showed that even though doctors knew that doxycycline plus streptomycin was more effective than doxycycline plus rifampicin, 64.6% of them still tend to prescribe convenient but less effective medications for patients [36]. Furthermore, additional studies indicate that despite being informed of the disadvantages of rifampicin compared to aminoglycoside drugs, most patients still prefer the convenience of oral medications [37]. As brucellosis occurs mostly in developing countries, and in remote areas where nomadic populations at high risk of exposure live without access to medical facilities, such as safe injections, and specialist health workers, it is particularly important to find treatment strategies that maximize cost-effectiveness and are easy to implement [15,38]. This may include measures such as the use of affordable antibiotics and simplification of treatment regimens to reduce treatment time.

We did not consider the impact of dosage and duration of therapy on treatment outcomes due to the length of the article. The dosage of most drugs used in the included studies was fairly uniform, especially doxycycline and streptomycin. A meta-analysis by Petros and colleagues [39] examined the relationship between duration of antimicrobial therapy and outcome and showed that brucellosis had better clinical and bacteriologic cure rates with longer antibiotic regimens. Nonetheless, longer treatment times mean higher costs, so it comes back to the above question of finding a balance between efficacy, safety, and cost of treatment to choose the best treatment option for patients. The use of risk-based categorization model and microbial load quantification to optimize personalized treatments may address this issue, especially in resource-limited settings [31,40].

Brucellosis patients with severe endocarditis and neurological disease are more difficult to treat and have a worse prognosis. There are no specific guidelines on the choice and duration of treatment for neuro-brucellosis; the commonly used treatment is a combination of 3 drugs that cross the blood–brain barrier, such as ceftriaxone, doxycycline, rifampicin, or TMP/SMX, and the duration of treatment depends on the patient’s response [3]. The treatment of brucellosis patients with concomitant endocarditis usually involves surgical intervention in addition to drug therapy. Research indicates that the surgical rate is 86.5% [41]. Furthermore, research has shown that combining surgery with drug therapy significantly reduces the mortality rate from 32.7% to 6.7% as compared to medication treatment alone [42]. Given the specificity of the treatment, we did not include the abovementioned types of cases in the scope of the meta-analysis. In addition, cases with severe complications may have interactions with the study results, leading to an overestimation or underestimation of their impact on the final outcomes when included. However, we did not exclude studies that included only a small percentage of patients with spondylitis brucellosis. This is because most studies did not report individual data on such patients, and we assumed that the grouping of such patients in an RCT would be randomized and balanced. Concerning meningitis, endophthalmitis, and other *Brucella*-related clinical syndromes, our inclusion criteria did not explicitly exclude them. While we did not exclude studies based on the presence of a small percentage of *Brucella*-related clinical syndromes, we did not actively seek out studies that focus on these conditions. Our primary focus is on the overall population of patients included in the studies, not necessarily on patients with specific *Brucella*-related syndromes.

Our study has limitations. Although we made every effort to include all available RCTs, we cannot rule out the possibility of missing information. Time to defervescence is a crucial indicator for assessing the speed of symptom improvement in brucellosis. Despite summarizing the reported time to defervescence from the included studies (**S6 Table**), only 16 out of 43 (37.2%) articles reported this indicator. Therefore, we did not consider it in the result analysis. In addition, certain nodes in our network consisted of only a few studies, resulting in wider confidence intervals for certain drug comparisons. Therefore, caution should be exercised when interpreting the results of these findings. Well-designed head-to-head pairwise studies with broader population coverage are needed in future research.

### Recommendations

**Table 2** summarizes the recommendations from our systematic review compared with previous recommendations. As we did not investigate dosage and treatment duration, we relied on the dosages commonly used in the included studies and those recommended in previous reviews. Based on the evidence in the current review, we recommend 6 weeks of doxycycline plus 1 to 2 weeks of gentamicin or plus 2 to 3 weeks of streptomycin treatment. As Rifampicin + Quinolones has a high safety profile and good efficacy, we recommend 6 weeks of treatment as an alternative therapy. If finances allow, consider triple therapy. Only one study explicitly reported adverse reactions caused by tetracycline (one lupus-like rash on the face and erythema related to tetracycline). Furthermore, in the league table (Fig 4), we found that the majority of drug treatment comparisons based on tetracycline drugs had a low or very low certainty of evidence. The SUCRA results in Fig 5 show that the effectiveness of Rifampicin + Tetracyclines or Streptomycin + Tetracyclines is very low. Although the safety of Streptomycin + Tetracyclines is the highest (only involving 3 trials), the safety of Rifampicin + Tetracyclines is very low. Therefore, we only consider Rifampicin + Tetracyclines as an alternative second-line therapy and Streptomycin + Tetracyclines as a medication we do not recommend. Monotherapy, Streptomycin + TMP/SMX, and Rifampicin + TMP/SMX also cannot currently be recommended.

**Table 2:** Recommendations for the treatment of human brucellosis.

	WHO/FAO 1986[11]	Ioannina 2007[15]	Skalsky 2008[21]	Yousefi-Nooraie 2012[29]	Solís García del Pozo 2012[31]	Current review*
First line regimen	D+R for 6 weeks	D for 6 weeks + S for 2-3 weeks	D+R for 6 weeks + G for 2 weeks OR D for 6 weeks + G for 2 weeks	D for 6 weeks + S for 2-3 weeks	D for 45 days + S for 14 days / G for 7 days	D for 6 weeks + G for 1-2 weeks OR D for 6 weeks + S for 2-3 weeks
Alternative	TET for 6 weeks + S for 2-3 weeks	D+R for 6 weeks	D for 6 weeks + S for 2 weeks	D+R for 6 weeks	D+R OR O+R	R+Quinolones for 6 weeks OR Triple therapy
Second line regimen	—	D for 6 weeks + G for 1 week	D+R for 6 weeks OR G/S for 2 weeks + TET for 6 weeks	—	D+TMP/SMX	D+R for 6 weeks OR D+TMP/SMX for 6-8 weeks
Optional, poor evidence	TMP/SMX	D+TMP/SMX+other for 6 weeks OR D+O/CIP +/− other for 6 weeks	D/R+TMP/SMX for 6 weeks	R+Q for 6 weeks	D+R+Aminoglycosides (Triple therapy)	R+Tetracyclines
Not recommended	—	Azithromycin OR Meropenem	Monotherapy OR <30 days of treatment OR D/R +/− Q	Monotherapy	—	Monotherapy OR S+Tetracyclines OR R/S+TMP/SMX

D = doxycycline, R = rifampicin, S = streptomycin, O = ofloxacin, TMP/SMX = cotrimoxazole, G = gentamicin, CEF = ceftriaxone, CIP = ciprofloxacin, TET = tetracycline.

Quinolones include ofloxacin, levofloxa, and ciprofloxacin; Tetracyclines include tetracycline, tetracycline hydrochloride, and Oxytetracycline; Aminoglycosides include streptomycin, gentamicin, and amikacin.

*Therapy is not suitable for children, pregnant women, and patients with focal lesions such as osteoarthritic brucellosis, endocarditis brucellosis, etc. Recommended duration of treatment (in table) and doses are based on those commonly used in previous systematic reviews and included studies: doxycycline 100mg twice daily, rifampicin 900mg once daily, streptomycin 1g once daily, gentamicin 5mg/kg/day, tetracycline hydrochloride and tetracycline 500mg four times daily, ofloxacin 400mg once daily, ceftriaxone 75mg/kg/day, ciprofloxacin 500-750mg twice daily.

Our recommendations are not suitable for children, pregnant women, and patients with focal lesions such as osteoarthritic brucellosis, endocarditis brucellosis, etc. In children younger than 8 years, the preferred regimen is rifampicin with TMP/SMX for 6 weeks. An alternative regimen involves a combination of rifampicin or TMP/SMX for 6 weeks, alongside gentamicin for the initial 5 to 10 days [3]. There are no RCTs on the treatment of brucellosis in pregnant women, so current recommendations are based primarily on observational studies. For more information, see related research reports.

## Supporting information

S1 Table PRISMA checklist. (DOCX)

S2 Table Revisions to the predetermined protocol, accompanied by justifications. (DOCX)

S3 Table Search strategy. (DOCX)

S4 Table Statistical methods in detail. (DOCX)

S5 Table The list of included studies. (DOCX)

S6 Table Additional details on included studies. (DOCX)

S7 Table List of excluded studies. (DOCX)

S8 Table Details on risk of bias and certainty of evidence assessments. (DOCX)

S9 Table Risk of bias of included studies. (DOCX)

S10 Table Assessments of certainty of evidence of primary outcomes using CINeMA. (DOCX)

S11 Table League table of secondary outcomes in network meta-analysis. (DOCX)

S12 Table Heterogeneity estimates. (DOCX)

S13 Table Inconsistency estimates. (DOCX)

S14 Table Subgroup analyses for primary outcomes. (DOCX)

S1 Fig Pairwise meta-analyses results. (DOCX)

S2 Fig The network plots for secondary outcome. (DOCX)

S3 Fig Sensitivity analyses. (DOCX)

S4 Fig Publication bias assessment. (DOCX)
