# Supplementary material for: Updated therapeutic options for human brucellosis: A systematic review and network meta-analysis of randomized controlled trials
Source: PLoS Negl Trop Dis. 2024 Aug 22;18(8):e0012405. doi: 10.1371/journal.pntd.0012405 (PMC11340890; doi:10.1371/journal.pntd.0012405)
Supplement: S1 Table — (DOCX) [file pntd.0012405.s001.docx]

**S1 Table**. PRISMA checklist

|  | Item # | Checklist Item | Reported on Page # |
| --- | --- | --- | --- |
| TITLE |  |  |  |
| Title | 1 | Identify the report as a systematic review incorporating a network  meta-analysis (or related form of meta-analysis). | #1 |
| ABSTRACT |  |  |  |
| Structured  summary | 2 | Provide a structured summary including, as applicable:  **Background:** main objectives  **Methods:** data sources; study eligibility criteria, participants,  and interventions; study appraisal; and synthesis methods, such  as network meta-analysis.  **Results:** number of studies and participants identified;  summary estimates with corresponding confidence/credible  intervals; treatment rankings may also be discussed. Authors  may choose to summarize pairwise comparisons against a  chosen treatment included in their analyses for brevity.  **Discussion/Conclusions:** limitations; conclusions and  implications of findings.  **Other:** primary source of funding; systematic review  registration number with registry name. | #2-3 |
| INTRODUCTION |  |  |  |
| Rationale | 3 | Describe the rationale for the review in the context of what is  already known, including mention of why a network meta-  analysis has been conducted. | #4-5 |
| Objectives | 4 | Provide an explicit statement of questions being addressed, with  reference to participants, interventions, comparisons, outcomes,  and study design (PICOS). | #6 |
| METHODS |  |  |  |
| Protocol and  registration | 5 | Indicate whether a review protocol exists and if and where it can  be accessed (e.g., Web address); and, if available, provide  registration information, including registration number. | #6 |
| Eligibility criteria | 6 | Specify study characteristics (e.g., PICOS, length of follow-up)  and report characteristics (e.g., years considered, language,  publication status) used as criteria for eligibility, giving rationale.  Clearly describe eligible treatments included in the treatment  network, and note whether any have been clustered or merged  into the same node (with justification). | #7 |
| Information  sources | 7 | Describe all information sources (e.g., databases with dates of  coverage, contact with study authors to identify additional  studies) in the search and date last searched. | #8 |
| Search | 8 | Present full electronic search strategy for at least one database,  including any limits used, such that it could be repeated. | #7 |
| Study selection | 9 | State the process for selecting studies (i.e., screening, eligibility,  included in systematic review, and, if applicable, included in the  meta-analysis). | #7-9 |
| Data collection  process | 10 | Describe method of data extraction from reports (e.g., piloted  forms, independently, in duplicate) and any processes for  obtaining and confirming data from investigators. | #8-9 |
| Data items | 11 | List and define all variables for which data were sought (e.g.,  PICOS, funding sources) and any assumptions and simplifications  made. | #8 |
| Geometry of the  network | **S1** | Describe methods used to explore the geometry of the treatment  network under study and potential biases related to it. This should  include how the evidence base has been graphically summarized  for presentation, and what characteristics were compiled and used  to describe the evidence base to readers. | _ |
| Risk of bias Within  individual studies | 12 | Describe methods used for assessing risk of bias of individual  studies (including specification of whether this was done at the  study or outcome level), and how this information is to be used in  any data synthesis. | #9  S4 Table |
| Summary measures | 13 | State the principal summary measures (e.g., risk ratio, difference  in means). Also describe the use of additional summary measures  assessed, such as treatment rankings and surface under the  cumulative ranking curve (SUCRA) values, as well as modified  approaches used to present summary findings from meta-  analyses. | #9  S4 Table |
| Planned methods  of analysis | 14 | Describe the methods of handling data and combining results of  studies for each network meta-analysis. This should include, but  not be limited to:  • Handling of multi-arm trials;  • Selection of variance structure;  • Selection of prior distributions in Bayesian analyses;  and  • Assessment of model fit. | #9  S4 Table |
| Assessment of  Inconsistency | **S2** | Describe the statistical methods used to evaluate the agreement of  direct and indirect evidence in the treatment network(s) studied.  Describe efforts taken to address its presence when found. | #9  S4 Table |
| Risk of bias across  studies | 15 | Specify any assessment of risk of bias that may affect the  cumulative evidence (e.g., publication bias, selective reporting  within studies). | #9  S4 Table |
| Additional analyses | 16 | Describe methods of additional analyses if done, indicating which  were pre-specified. This may include, but not be limited to, the  following:  • Sensitivity or subgroup analyses;  • Meta-regression analyses;  • Alternative formulations of the treatment network; and  • Use of alternative prior distributions for Bayesian  analyses (if applicable). | #9  S4 Table |
| RESULTS† |  |  |  |
| Study selection | 17 | Give numbers of studies screened, assessed for eligibility, and  included in the review, with reasons for exclusions at each stage,  ideally with a flow diagram. | #10  Fig 1 |
| Presentation of  network structure | **S3** | Provide a network graph of the included studies to enable  visualization of the geometry of the treatment network. | Fig 3  S2 Fig |
| Summary of  Network geometry | **S4** | Provide a brief overview of characteristics of the treatment  network. This may include commentary on the abundance of  trials and randomized patients for the different interventions and  pairwise comparisons in the network, gaps of evidence in the  treatment network, and potential biases reflected by the network  structure. | #10 |
| Study  characteristics | 18 | For each study, present characteristics for which data were  extracted (e.g., study size, PICOS, follow-up period) and provide  the citations. | #10  Table 1  S6 Table |
| Risk of bias within  studies | 19 | Present data on risk of bias of each study and, if available, any  outcome level assessment. | #11-12  S8 Table  S9 Table  S10 Table |
| Results of  individual studies | 20 | For all outcomes considered (benefits or harms), present, for each  study: 1) simple summary data for each intervention group, and 2)  effect estimates and confidence intervals. Modified approaches  may be needed to deal with information from larger networks. | #11-12  Fig 2  S1 Fig |
| Synthesis of results | 21 | Present results of each meta-analysis done, including  confidence/credible intervals. In larger networks, authors may  focus on comparisons versus a particular comparator (e.g.  placebo or standard care), with full findings presented in an  appendix. League tables and forest plots may be considered to  summarize pairwise comparisons. If additional summary  measures were explored (such as treatment rankings), these  should also be presented. | #13-14  Fig 4  Fig 5  S11 Table |
| Exploration for  inconsistency | **S5** | Describe results from investigations of inconsistency. This may  include such information as measures of model fit to compare  consistency and inconsistency models, P values from statistical  tests, or summary of inconsistency estimates from different parts  of the treatment network. | #13-14  S12 Table  S13 Table |
| Risk of bias across  studies | 22 | Present results of any assessment of risk of bias across studies for  the evidence base being studied. | S4 Fig |
| Results of  additional analyses | 23 | Give results of additional analyses, if done (e.g., sensitivity or  subgroup analyses, meta-regression analyses, alternative network  geometries studied, alternative choice of prior distributions for  Bayesian analyses, and so forth). | #14  S3 Fig  S14 Table |
| DISCUSSION |  |  |  |
| Summary of  evidence | 24 | Summarize the main findings, including the strength of evidence  for each main outcome; consider their relevance to key groups  (e.g., healthcare providers, users, and policy-makers). | #14-19 |
| Limitations | 25 | Discuss limitations at study and outcome level (e.g., risk of bias),  and at review level (e.g., incomplete retrieval of identified  research, reporting bias). Comment on the validity of the  assumptions, such as transitivity and consistency. Comment on  any concerns regarding network geometry (e.g., avoidance of  certain comparisons). | #19 |
| Conclusions | 26 | Provide a general interpretation of the results in the context of  other evidence, and implications for future research. | #19-21 |
| FUNDING |  |  |  |
| Funding | 27 | Describe sources of funding for the systematic review and other  support (e.g., supply of data); role of funders for the systematic  review. This should also include information regarding whether  funding has been received from manufacturers of treatments in  the network and/or whether some of the authors are content  experts with professional conflicts of interest that could affect use  of treatments in the network. | _ |
