# Supplementary material for: Updated therapeutic options for human brucellosis: A systematic review and network meta-analysis of randomized controlled trials
Source: PLoS Negl Trop Dis. 2024 Aug 22;18(8):e0012405. doi: 10.1371/journal.pntd.0012405 (PMC11340890; doi:10.1371/journal.pntd.0012405)
Supplement: S2 Table — (DOCX) [file pntd.0012405.s002.docx]

**S2 Table**. Revisions to the predetermined protocol, accompanied by justifications

**1.1 Types of study to be included**

The original, pre-specified protocol stated: “The studies included in this study are randomized controlled trials and quasi-randomized controlled trials”. The quasi-randomized trial did not adhere to randomization principles in grouping. Therefore, to ensure the credibility of the evidence, only randomized controlled trials were ultimately included.

**1.2 Main outcomes**

The original, pre-specified protocol stated: “Our two predefined primary outcomes were “relapse”, defined as the re-appearance of relevant clinical symptoms, rise in antibody titers or positive results on cultures after the end of treatment, during the follow-up period; and “overall failure”, defined as the sum of relapse and therapeutic failure”. To reflect the efficacy and safety of the drug, we later established two primary outcome measures: overall failure and adverse reactions. Overall failure was defined as the sum of relapse and therapeutic failure; and side effects, defined as uncomfortable symptoms that occur during the administration of the drug, such as nausea and vomiting, abdominal pain and diarrhea, etc., serious side effects such as ototoxicity, hepatotoxicity, nephrotoxicity, and skin reactions.

**1.3 Additional outcomes**

The original, pre-specified protocol stated: “Additional outcome indicators include therapeutic failure and side effects. Therapeutic failure is defined as a patient's symptoms not improving by the end of treatment; Side effects are defined as uncomfortable symptoms that occur during the administration of the drug, such as nausea and vomiting, abdominal pain and diarrhea, etc. Serious side effects such as ototoxicity, hepatotoxicity, nephrotoxicity and skin reactions”. Modified expression: Secondary outcomes included relapse, defined as the reappearance of relevant clinical symptoms, rise in antibody titers or positive results on cultures after the end of treatment, during the follow-up period; and therapeutic failure, defined as patient’s symptoms not improving by the end of treatment.
