# Supplementary material for: Updated therapeutic options for human brucellosis: A systematic review and network meta-analysis of randomized controlled trials
Source: PLoS Negl Trop Dis. 2024 Aug 22;18(8):e0012405. doi: 10.1371/journal.pntd.0012405 (PMC11340890; doi:10.1371/journal.pntd.0012405)
Supplement: S3 Table — (DOCX) [file pntd.0012405.s003.docx]

**S3 Table**. Search strategy

**PubMed (13/12/2023)**

#1 Brucellosis[MeSH Terms]

#2 Brucella[MeSH Terms]

#3 (((((((((((((((((((Brucellosis[Title/Abstract]) OR (Brucella[Title/Abstract])) OR (Brucelloses[Title/Abstract])) OR (Malta Fever[Title/Abstract])) OR (Fever, Malta[Title/Abstract])) OR (Gibraltar Fever[Title/Abstract])) OR (Fever, Gibraltar[Title/Abstract])) OR (Rock Fever[Title/Abstract])) OR (Fever, Rock[Title/Abstract])) OR (Cyprus Fever[Title/Abstract])) OR (Fever, Cyprus[Title/Abstract])) OR (Brucella Infection[Title/Abstract])) OR (Brucella Infections[Title/Abstract])) OR (Infection, Brucella[Title/Abstract])) OR (Undulant Fever[Title/Abstract])) OR (Fever, Undulant[Title/Abstract])) OR (Brucellosis, Pulmonary[Title/Abstract])) OR (Brucelloses, Pulmonary[Title/Abstract])) OR (Pulmonary Brucelloses[Title/Abstract])) OR (Pulmonary Brucellosis[Title/Abstract])

#4 #1 OR #2 OR #3

#5 ((((((((randomized controlled trial[Publication Type]) OR (controlled clinical trial[Publication Type])) OR (randomized[Title/Abstract])) OR (placebo[Title/Abstract]))) OR (drug therapy[MeSH Subheading])) OR (randomly[Title/Abstract])) OR (trial[Title/Abstract])) OR (groups[Title/Abstract])

#6 (animals[MeSH Terms]) NOT (humans[MeSH Terms])

#7 #5 NOT #6

#8 #4 AND #7

**Embase (13/12/2023)**

('Brucellosis'/exp OR 'Brucella'/exp OR Brucelloses:ab,ti OR 'Malta Fever':ab,ti OR 'Malta Fever':ab,ti OR 'Fever, Malta':ab,ti OR 'Gibraltar Fever':ab,ti OR 'Fever, Gibraltar':ab,ti OR 'Rock Fever':ab,ti OR 'Fever, Rock':ab,ti OR 'Cyprus Fever':ab,ti OR 'Fever, Cyprus':ab,ti OR 'Brucella Infection':ab,ti OR 'Brucella Infections':ab,ti OR 'Infection, Brucella':ab,ti OR 'Undulant Fever':ab,ti OR 'Fever, Undulant':ab,ti OR 'Brucellosis, Pulmonary':ab,ti OR 'Brucelloses, Pulmonary':ab,ti OR 'Pulmonary Brucelloses':ab,ti OR 'Pulmonary Brucellosis':ab,ti) AND ('crossover procedure':de OR 'double-blind procedure':de OR 'randomized controlled trial':de OR 'single-blind procedure':de OR (random* OR factorial* OR crossover* OR cross NEXT/1 over* OR placebo* OR doubl* NEAR/1 blind* OR singl* NEAR/1 blind* OR assign* OR allocat* OR volunteer*):de,ab,ti)

**Web of Science (13/12/2023)**

TS=(Brucellosis OR Brucella OR Brucelloses OR Malta Fever OR Fever, Malta OR Gibraltar Fever OR Fever, Gibraltar OR Rock Fever OR Fever, Rock OR Cyprus Fever OR Fever, Cyprus OR Brucella Infection OR Brucella Infections OR Infection, Brucella OR Undulant Fever OR Fever, Undulant OR Brucellosis, Pulmonary OR Brucelloses, Pulmonary OR Pulmonary Brucelloses OR Pulmonary Brucellosis) AND TS=(clinical trial* OR research design OR comparative stud* OR evaluation stud* OR controlled trial* OR follow-up stud* OR prospective stud* OR random* OR placebo* OR single blind* OR double blind*)

**Cochrane Library (13/12/2023)**

#1 MeSH descriptor: [Brucellosis] explode all trees

#2 MeSH descriptor: [Brucella] explode all trees

#3 (Brucellosis):ti,ab,kw OR (Brucella):ti,ab,kw OR (Brucelloses):ti,ab,kw OR (Malta Fever):ti,ab,kw OR (Fever, Malta):ti,ab,kw

#4 (Gibraltar Fever):ti,ab,kw OR (Fever, Gibraltar):ti,ab,kw OR (Rock Fever):ti,ab,kw OR (Fever, Rock):ti,ab,kw OR (Cyprus Fever):ti,ab,kw

#5 (Fever, Cyprus):ti,ab,kw OR (Brucella Infection):ti,ab,kw OR (Brucella Infections):ti,ab,kw OR (Infection, Brucella):ti,ab,kw OR (Undulant Fever):ti,ab,kw

#6 (Fever, Undulant):ti,ab,kw OR (Brucellosis, Pulmonary):ti,ab,kw OR (Brucelloses, Pulmonary):ti,ab,kw OR (Pulmonary Brucelloses):ti,ab,kw OR (Pulmonary Brucellosis):ti,ab,kw

#7 #1 OR #2 OR #3 OR #4 OR #5 OR #
