# Supplementary material for: Updated therapeutic options for human brucellosis: A systematic review and network meta-analysis of randomized controlled trials
Source: PLoS Negl Trop Dis. 2024 Aug 22;18(8):e0012405. doi: 10.1371/journal.pntd.0012405 (PMC11340890; doi:10.1371/journal.pntd.0012405)
Supplement: S4 Table — (DOCX) [file pntd.0012405.s004.docx]

**S4 Table**. Statistical methods in detail

We chose the standard treatment recommended by the WHO as the control treatment. This is because it is the intervention most closely linked to other interventions in the network [1]. Most pharmacological interventions were evaluated in single or a few small-scale randomized controlled trials. Therefore, we mainly conducted analyses for medication categories when relevant groupings were considered. Firstly, we did a pairwise meta-analysis with random effects model and assessed statistical heterogeneity. By calculating the I² statistic and its 95% confidence intervals (CIs), we assessed statistical heterogeneity within each pairwise comparison. We considered heterogeneity ranging from 0%-40% as potentially unimportant, 30%-60% as moderate, 50%-90% as substantial, and 75%-100% as critical [2]. Secondly, we did the network meta-analysis with class-level and individual data using random effects model in a frequentist framework. The binomial likelihood was used for dichotomous outcomes. The network meta-analysis employs relative risk (RR) with 95% CIs as the measure of treatment effect. We utilized the total number of patients in each arm and the events for each outcome to compute the RR and its associated 95% CIs using the pairwise function. In instances where both arms reported 0 events, we applied a continuity correction by adding 0.5 to both the event and total numbers [3]. A network meta-analysis yields network estimates from the aggregated results of both direct (pairwise, conventional meta-analysis) and indirect evidence (drug treatments with common comparators). For continuous data, we calculated missing SDs from p values, t values, and standard errors, or imputed them with a validated method [4]. We assumed a common heterogeneity variance τ² across the various treatment comparisons and accounted for correlations induced by multi-arm studies. We based the assessment of statistical heterogeneity in the entire network on the magnitude of the common τ² estimated from the network meta-analysis models and compared the magnitude of the heterogeneity variance with the empirical distribution. We conducted a statistical assessment of both local and global inconsistency, representing the agreement between direct and indirect evidence. This evaluation was carried out using the loop-specific approach and the design-by-treatment test, focusing on direct and indirect evidence separately. And the node-splitting approach to calculate inconsistency for each comparison [5, 6]. To describe and present the geometry of the intervention network, a network plot was created. The surface under the cumulative ranking curve (SUCRA) value was used to reflect the possibility of the intervention measures. Regardless of the heterogeneity of the results, we performed subgroup and sensitivity analyses to assess the effect of subgroup factors on the results and the stability of the results. Our predetermined subgroup comparisons were geographical region (other regions versus China) and follow-up time (more than six months versus less than or equal to six months). The stability of our findings was examined in analyses done by excluding sets of studies with the following characteristics: The number of study participants was less than 50; lack of follow-up time; with high risk of bias. We assessed the risk of bias of randomized controlled trials with the Cochrane tool [7]. We evaluated the confidence in estimates of the primary outcomes in the findings from the network meta-analysis with the Confidence In Network Meta-Analysis (CINeMA) framework implemented in semi-automated method through the web application: Https://cinema.ispm.unibe.ch/. The comparison-adjusted funnel plot and Egger’s test will be used to evaluate small-study effects at the network level [8]. Statistical evaluation of inconsistency and production of network graphs and result figures were done using the network and network graphs packages in Stata (version 17.0). Pairwise meta-analyses and statistical heterogeneity in the entire network was assessed using meta and netmeta package in R (version 4.3.1), respectively.

**References**

1. Brignardello-Petersen R, Izcovich A, Rochwerg B, et al. GRADE approach to drawing conclusions from a network meta-analysis using a partially contextualised framework. BMJ. 2020;371:m3907. Published 2020 Nov 10. doi:10.1136/bmj.m3907

2. Cumpston M, Li T, Page MJ, et al. Updated guidance for trusted systematic reviews: a new edition of the Cochrane Handbook for Systematic Reviews of Interventions. Cochrane Database Syst Rev. 2019;10(10):ED000142. doi:10.1002/14651858.ED000142

3. Weber F, Knapp G, Ickstadt K, Kundt G, Glass Ä. Zero-cell corrections in random-effects meta-analyses. Res Synth Methods. 2020;11(6):913-919. doi:10.1002/jrsm.1460

4. Furukawa TA, Barbui C, Cipriani A, Brambilla P, Watanabe N. Imputing missing standard deviations in meta-analyses can provide accurate results. J Clin Epidemiol. 2006;59(1):7-10. doi:10.1016/j.jclinepi.2005.06.006

5. Higgins JP, Jackson D, Barrett JK, Lu G, Ades AE, White IR. Consistency and inconsistency in network meta-analysis: concepts and models for multi-arm studies. Res Synth Methods. 2012;3(2):98-110. doi:10.1002/jrsm.1044

6. Dias S, Welton NJ, Caldwell DM, Ades AE. Checking consistency in mixed treatment comparison meta-analysis. Stat Med. 2010;29(7-8):932-944. doi:10.1002/sim.3767

7. Sterne JAC, Savović J, Page MJ, et al. RoB 2: a revised tool for assessing risk of bias in randomised trials. BMJ. 2019;366:l4898. Published 2019 Aug 28. doi:10.1136/bmj.l4898

8. Chaimani A, Higgins JP, Mavridis D, Spyridonos P, Salanti G. Graphical tools for network meta-analysis in STATA. PLoS One. 2013;8(10):e76654. Published 2013 Oct 3. doi:10.1371/journal.pone.0076654
