# Supplementary material for: Updated therapeutic options for human brucellosis: A systematic review and network meta-analysis of randomized controlled trials
Source: PLoS Negl Trop Dis. 2024 Aug 22;18(8):e0012405. doi: 10.1371/journal.pntd.0012405 (PMC11340890; doi:10.1371/journal.pntd.0012405)
Supplement: S5 Table — (DOCX) [file pntd.0012405.s005.docx]

**S5 Table**. The list of included studies

w1. Acocella G, Bertrand A, Beytout J, et al. Comparison of three different regimens in the treatment of acute brucellosis: a multicenter multinational study. J Antimicrob Chemother 1989;23(3):433-9. doi: 10.1093/jac/23.3.433

w2. Agalar C, Usubutun S, Turkyilmaz R. Ciprofloxacin and rifampicin versus doxycycline and rifampicin in the treatment of brucellosis. Eur J Clin Microbiol Infect Dis 1999;18(8):535-8. doi: 10.1007/s100960050344

w3. Akova M, Uzun O, Akalin HE, et al. Quinolones in treatment of human brucellosis: comparative trial of ofloxacin-rifampin versus doxycycline-rifampin. Antimicrob Agents Chemother 1993;37(9):1831-4. doi: 10.1128/aac.37.9.1831

w4. Alavi S, Rajabzadeh A. Comparison of two chemotherapy regimen: Doxycycline-rifampicin and doxycycline cotrimoxazol in the brucellosis patients Ahwaz, Iran, 2004-2006. Pakistan Journal of Medical Sciences 2007;23(6):889.

w5. Ariza J, Gudiol F, Pallares R, et al. Treatment of human brucellosis with doxycycline plus rifampin or doxycycline plus streptomycin. A randomized, double-blind study. Ann Intern Med 1992;117(1):25-30. doi: 10.7326/0003-4819-117-1-25

w6. Buzon L, Bouza E, Rodriguez M. Treatment of brucellosis with rifampicin+tetracycline vs TMP/SMZ. A prospective and randomized study. Chemioterapia 1982;1(4 Suppl.):No. 221.

w7. Baoshan C, Dongmei X. Efficacy and safety of doxycycline combined with levofloxacin in the treatment of brucellosis. IMHGN 2018;24(1):94-96,132. (in Chinese). doi: 10.3760/cma.j.issn.1007-1245.2018.01.030

w8. Ruolei C. Clinical observation of doxycycline combined with levofloxacin in the treatment of brucellosis. Guide China Med 2016;14(26):68. (in Chinese). doi: 10.15912/j.cnki.gocm.2016.26.060

w9. Colmenero Castillo JD, Hernandez Marquez S, Reguera Iglesias JM, et al. Comparative trial of doxycycline plus streptomycin versus doxycycline plus rifampin for the therapy of human brucellosis. Chemotherapy 1989;35(2):146-52. doi: 10.1159/000238662

w10. Colmenero JD, Fernández-Gallardo LC, Agúndez JA, et al. Possible implications of doxycycline-rifampin interaction for treatment of brucellosis. Antimicrob Agents Chemother 1994;38(12):2798-802. doi: 10.1128/aac.38.12.2798

w11. Lili D, Junyan Z. Clinical efficacy of rifampicin combined with doxycycline in the treatment of brucellosis. Women's Health Res 2016(9):222,21. (in Chinese).

w12. Ersoy Y, Sonmez E, Tevfik MR, et al. Comparison of three different combination therapies in the treatment of human brucellosis. Trop Doct 2005;35(4):210-2. doi: 10.1258/004947505774938765

w13. Lifu G, Xiumei W. Clinical effects, complications, and quality-of-life scores of doxycycline and levofloxacin combination in the treatment of brucellosis. Women's Health Res 2023(14):56-58. (in Chinese).

w14. Hasanain A, Mahdy R, Mohamed A, et al. A randomized, comparative study of dual therapy (doxycycline-rifampin) versus triple therapy (doxycycline-rifampin-levofloxacin) for treating acute/subacute brucellosis. Braz J Infect Dis 2016;20(3):250-4. doi: 10.1016/j.bjid.2016.02.004

w15. Hasanjani Roushan MR, Mohraz M, Hajiahmadi M, et al. Efficacy of gentamicin plus doxycycline versus streptomycin plus doxycycline in the treatment of brucellosis in humans. Clin Infect Dis 2006;42(8):1075-80. doi: 10.1086/501359

w16. Hashemi SH, Gachkar L, Keramat F, et al. Comparison of doxycycline-streptomycin, doxycycline-rifampin, and ofloxacin-rifampin in the treatment of brucellosis: a randomized clinical trial. Int J Infect Dis 2012;16(4):e247-51. doi: 10.1016/j.ijid.2011.12.003

w17. Hassan WA, Abdel-Gawad M, Abdelmohsen AS. Doxycycline Plus Trimethoprim-Sulfamethoxazole versus Doxycycline plus Rifampicin in Treatment of Brucellosis: A Randomized Controlled Trial. Al-Azhar Assiut Med J 2022;20:333–37.

w18. Roushan MR, Amiri MJ, Janmohammadi N, et al. Comparison of the efficacy of gentamicin for 5 days plus doxycycline for 8 weeks versus streptomycin for 2 weeks plus doxycycline for 45 days in the treatment of human brucellosis: a randomized clinical trial. J Antimicrob Chemother 2010;65(5):1028-35. doi: 10.1093/jac/dkq064

w19. Li J. Comparative efficacy of different antimicrobial drug combination regimens in the treatment of brucellosis. Chin J Clinical Ration Drug Use 2020;13(10):53-54. (in Chinese). doi: 10.15887/j.cnki.13-1389/r.2020.10.030.

w20. Lili J, Bing Z, Wei L. Clinical efficacy of doxycycline hydrochloride combined with rifampicin in the treatment of brucellosis. Chin J Ctrl Endem Dis 2022;37(05):439+41. (in Chinese).

w21. Kalo T, Novi S, Nushi A, et al. Ciprofloxacin plus doxycycline versus rifampicin plus doxycycline in the treatment of acute brucellosis. Médecine et Maladies Infectieuses 1996;26:587-89. doi: https://doi.org/10.1016/S0399-077X(96)80077-1

w22. Karabay O, Sencan I, Kayas D, et al. Ofloxacin plus rifampicin versus doxycycline plus rifampicin in the treatment of brucellosis: a randomized clinical trial [ISRCTN11871179]. BMC Infect Dis 2004;4:18. doi:10.1186/1471-2334-4-18

w23. Keramat F, Ranjbar M, Mamani M, Hashemi SH, Zeraati F. A comparative trial of three therapeutic regimens: ciprofloxacin-rifampin, ciprofloxacin-doxycycline and doxycycline-rifampin in the treatment of brucellosis. Trop Doct 2009;39(4):207-210. doi:10.1258/td.2009.090030

w24. Lang R, Raz R, Sacks T, Shapiro M. Failure of prolonged treatment with ciprofloxacin in acute infections due to Brucella melitensis. J Antimicrob Chemother 1990;26(6):841-846. doi:10.1093/jac/26.6.841

w25. Lang R, Dagan R, Potasman I, Einhorn M, Raz R. Failure of ceftriaxone in the treatment of acute brucellosis. Clin Infect Dis 1992;14(2):506-509. doi:10.1093/clinids/14.2.506

w26. Dan L. Observation on efficacy of rifampin combined with doxycycline in the treatment of acute brucellosis. China Prac Med 2018;13(04):77-79. (in Chinese). doi: 10.14163/j.cnki.11-5547/r.2018.04.047

w27. Jin L, Aijun L, Weidong M. Efficacy of doxycycline combined with compound sulfamethoxazole in the treatment of acute-phase brucellosis. Lab Med Clin 2019;16(22):3376-78. (in Chinese).

w28. Montejo JM, Alberola I, Glez-Zarate P, et al. Open, randomized therapeutic trial of six antimicrobial regimens in the treatment of human brucellosis. Clin Infect Dis 1993;16(5):671-6. doi: 10.1093/clind/16.5.671

w29. Longjiang Q, Jianjun G, Junfeng Z, et al. Observation on the effect of levofloxacin on acute brucellosis cases. China Tropical Medicine 2008(12):2135+63. (in Chinese).

w30. Longjiang Q, Jianjun G, Yihua Z. Efficacy of levofloxacin combined with doxycycline in the treatment of acute brucellosis in 42 cases. Med Her 2009;28(03):331-32. (in Chinese).

w31. Ranjbar M, Keramat F, Mamani M, et al. Comparison between doxycycline-rifampin-amikacin and doxycycline-rifampin regimens in the treatment of brucellosis. Int J Infect Dis 2007;11(2):152-6. doi: 10.1016/j.ijid.2005.11.007

w32. Roushan MR, Gangi SM, Ahmadi SA. Comparison of the efficacy of two months of treatment with co-trimoxazole plus doxycycline vs. co-trimoxazole plus rifampin in brucellosis. Swiss Med Wkly 2004;134(37-38):564-8. doi: 10.4414/smw.2004.10665

w33. Sarmadian H, Didgar F, Sufian M, et al. Comparison Between Efficacy of Cipofoxacin Doxycycline and Rifampin - Doxycycline Regimens in Treatment and Relapse of Brucellosis. Tropical Medicine & International Health 2009;14:209-09.

w34. Rina S. Comparison of different combination therapy regimens of antimicrobial drugs for brucellosis. World Latest Medicine Inf 2017;17(58):127-28. (in Chinese). doi: 10.19613/j.cnki.1671-3141.2017.58.067

w35. Hongtao S. Clinical Observation of Rifampicin Doxycycline and Levofloxacin in the Treatment of Brucellosis. China Health Stand Management 2015;6(22):123-24. (in Chinese).

w36. Lihui S. Effects of triple therapy of Ofloxacin, Rifampicin and Doxycycline in treatment of patients with brucellosis. Med J Chin People's Health 2020;32(24):20-21. (in Chinese).

w37. Qian S, Lei F, Xiaowei Z. Comparison of the therapeutic effects of different antibacterial drugs combined in the treatment of adult brucellosis patients. Chin J Endemiol 2023;42(6):498-501. (in Chinese). doi: 10.3760/cma.j.cn231583-20220411-00115

w38. Fenglan W. Analysis of the effect of doxycycline combined with levofloxacin in the treatment of brucellosis. China Health Care & Nutrition 2020;30(1):279-80. (in Chinese).

w39. Na W. Evaluation of the effect and total effective rate of doxycycline combined with levofloxacin in the treatment of patients with brucellosis. Fertility & Health 2022;28(19):149-51. (in Chinese).

w40. Meng Y, Yanhong W, Yu S, et al. Comparison of different combination therapy for acute brucellosis. Chin J Exp Clin Infect Dis 2015;9(06):81-83. (in Chinese).

w41. Cui Z, Zhe W, Yuejie Y, et al. Effect of Rifampicin Combined with Doxycycline and Levofloxacin in the Treatment of Brucellosis. J Med Inf 2022;35(03):112-14. (in Chinese). doi: 10.3969/j.issn.1006-1959.2022.03.027

w42. Xiyan Z. Efficacy and Safety of Levofloxacin based Triple Antibiotic Regimen in the Treatment of Brucellosis. China J Pharmaceutical Economics 2023;18(09):32-35. (in Chinese). doi: 10.12010/j.issn.1673-5846.2023.09.006

w43. Yan Z. Comparison of the efficacy of two treatment options for brucellosis. Shihezi University, 2017. (in Chinese).
