# Supplementary material for: Updated therapeutic options for human brucellosis: A systematic review and network meta-analysis of randomized controlled trials
Source: PLoS Negl Trop Dis. 2024 Aug 22;18(8):e0012405. doi: 10.1371/journal.pntd.0012405 (PMC11340890; doi:10.1371/journal.pntd.0012405)
Supplement: S6 Table — (DOCX) [file pntd.0012405.s006.docx]

**S6 Table**. Additional details on included studies

| **Study** | **Intervention** | **Inclusion criteria** | **Exclusion criteria** | **Time to defervescence (mean ± sd, days)** | **Number of patients** | | | |
| --- | --- | --- | --- | --- | --- | --- | --- | --- |
|  |  |  |  |  | **Relapse** | **Therapeutic failure** | **Overall failure** | **Side effects** |
| Acocella 1989[w1] | DR | (i) Clinical picture compatible with acute brucellosis and (ii) Standard Tube agglutination Test above 125 IU, or complement fixation positive at a dilution of 1/8 or more, or positive blood culture for brucella | Severe concomitant diseases, need for corticosteroids, barbiturates, or other antibiotics, pregnancy, age <10 or >70, and allergy to any of the four antibiotics to be used | NR | 3 | 0 | 3 | 0 |
|  | DS |  |  |  | 0 | 2 | 2 | 8 |
|  | S+TC |  |  |  | 6 | 5 | 11 | 1 |
| Agalar 1999[w2] | DR | Patients suspected to have Brucella infection based on clinical and laboratory findings | Age under 15 years, history of seizures, recent antibiotic use, allergy to the study antibiotics, and pregnancy and with blood cultures negative for Brucella melitensis | 3.85 ± 1.98 | 2 | 0 | 2 | 0 |
|  | R+CIP |  |  | 2.78 ± 1.03 | 3 | 0 | 3 | 0 |
| Akova 1993[w3] | DR | A standard tube agglutination titer of 1/160 or more for anti-Brucella antibodies in the presence of compatible clinical findings (fever, night sweats, arthralgia, hepatomegaly, splenomegaly, and lymphadenopathy) and isolation of a Brucella sp. from blood or bone marrow cultures | Patients with endocarditis or neurobrucellosis. Individuals who received antimicrobial therapy prior to the study, pregnant women, and patients allergic to any of the drugs employed in the regimens | 5.1 (2-10) | 1 | 0 | 1 | 1 |
|  | RO |  |  | 6.3 (2-18) | 1 | 1 | 2 | 2 |
| Alavi 2007[w4] | DR | The diagnostic criteria were the finding of >1/ 80 standard tube agglutination titer (STAT) of antibodies to brucella (Wright) with a 2 mercaptoethanol (2 ME) >1/40, in association with compatible clinical findings | Age less than 15 years, pregnancy, spondylitis, endocarditis, meningoencephalitis, previous history of brucellosis, and antimicrobial therapy for more than seven days before enrollment | NR | 6 | 5 | 11 | 0 |
|  | D+TMP/SMX |  |  |  | 3 | 1 | 4 | 2 |
| Ariza 1992[w5] | DR | A standard tube agglutination titer of 1/160 or more for anti-Brucella or had characteristic clinical findings (fever, sweats, arthralgias, hepatomegaly, splenomegaly, and lymphadenopathy) | Endocarditis, neurobrucellosis | 4.2 (1-45) | 3 | 2 | 5 | 25 |
|  | DS |  |  | 3.2 (1-14) | 2 | 1 | 3 | 15 |
| Buzon 1982[w6] | R+TC | Patients with acute brucellosis diagnosed through isolation of Brucella and/or seroconversion with a compatible clinical setting | NR | NR | NR | 8 | 8 | 0 |
|  | TMP/SMX |  |  |  |  | 17 | 17 | 8 |
| Chai 2018[w7] | DL | Presence of a history of epidemiologic exposure with significant joint pain and malaise, clinical symptoms such as fever and a positive brucellosis agglutination test | Combination of liver and kidney function abnormalities, mental system diseases and malignant tumors | NR | NR | 2 | 2 | 6 |
|  | L |  |  |  |  | 11 | 11 | 4 |
| Chen 2016[w8] | DL | History of epidemiologic exposure, clinical symptoms such as prolonged fever, excessive sweating and joint pain, exclusion of other diseases, and positive brucellosis agglutination test | NR | NR | 1 | NR | 1 | 3 |
|  | CIP |  |  |  | 4 |  | 4 | 2 |
| Colmenero 1989[w9] | DR | (1) Isolation of Brucella from blood or from any other body fluid and/or (2) clinical picture compatible with the disease together with (i) Wright’s séroagglutination at titres equal to or higher than 1/160; or (ii) indirect immunofluorescence with titres equal to or higher than 1/100 for the IgS or IgG conjugates and equal to or higher than 1/50 for IgM or IgA conjugates, or (iii) seroconversion of four or more times the initial titres in two separate serum samples taken with in a minimum interval of 3 weeks between them | Patients with neuromeningeal complications or those treated within the preceding 96 h with either tetracycline, streptomycin, rifampin, or co-trimoxazole | 3.5 | 7 | 0 | 7 | 6 |
|  | DS |  |  | 3.5 | 3 | 2 | 5 | 7 |
| Colmenero 1994[w10] | DR | A diagnosis of brucellosis was made by the isolation of the organism or the presence of compatible clinical features together with the demonstration of specific antibodies at significant titers. Significant titers were considered to be >= 1/160 for seroagglutination and >= 1/320 by the anti-Brucella Coombs test | Abnormal hepatic and renal functions, and received any drugs or took any alcohol during the treatment period | NR | 1 | 1 | 2 | NR |
|  | DS |  |  |  | 0 | 0 | 0 |  |
| Deng 2016[w11] | DR | Meets diagnostic criteria for brucellosis | NR | 6 ± 3 | NR | 1 | 1 | NR |
|  | D |  |  | 9 ± 5 |  | 6 | 6 |  |
| Ersoy 2005[w12] | DR | (i) Isolation of Brucella spp. from blood or other ﬂuids, or (ii) the ﬁnding of X1/160 titre or four-fold rise over 2–3 weeks in titre of antibodies to Brucella by a standard-tube agglutination test | Patients were pregnancy or nursing, known or suspected hypersensitivity or any contraindication to rifampicines, tetracyclines or aminoglycosides, severe concomitant disease and effective antimicrobial therapy within 10 days before starting the study | NR | 6 | 1 | 7 | 21 |
|  | DS |  |  |  | 3 | 1 | 4 | 8 |
|  | RO |  |  |  | 5 | 1 | 6 | 8 |
| Guo 2023[w13] | DL | Meets diagnostic criteria for Brucella abortus; positive results on clinical Brucella agglutination test; at least 2 typical symptoms of Brucella abortus; age >18 years; good tolerance to the drugs used in this study; complete clinical profile | Combination of malignant tumors; combination of organic pathologies such as liver and kidney; pregnant or breastfeeding women; participating in other studies at our institution | NR | NR | 3 | 3 | 7 |
|  | L |  |  |  |  | 9 | 9 | 4 |
| Hasanain 2016[w14] | DRL | (1) contact with animals or fresh animal products, (2) suggestive clinical manifestations of less than one-year duration (fever, chills, sweats, fatigue, arthralgia, myalgia, relative bradycardia, splenomegaly, lymphadenopathy, and hepatomegaly), and (3) positive antibody titer (1:160) by standard tube agglutination test (against Brucella abortus, Brucella melitensis, and Brucella suis) | Pregnant and pediatric patients | NR | 5 | 6 | 11 | 11 |
|  | DR |  |  |  | 12 | 7 | 19 | 6 |
| Hasanjani Roushan 2006[w15] | DS | Patients with a Standard Tube Agglutination Test (STAT) titer >=1:320 and 2-Mercaptoethanol (2ME) titer >= 1:80 who had clinical findings compatible with this diagnosis | Age of <10 years, spondylitis, neurobrucellosis, pregnancy, and receipt of 11 week of antibiotic treatment before enrollment | NR | 3 | 4 | 7 | 23 |
|  | DG |  |  |  | 3 | 2 | 5 | 27 |
| Hashemi 2011[w16] | DR | Clinical presentation compatible with brucellosis in the presence of significant titers of specific antibodies (standard tube agglutination 1/160, Coombs test 1/160, 2-mercaptoetanol (2-ME) 1-80, or Brucella IgG-ELISA >12) and/or a positive blood culture | Age under 17 years, endocarditis, neurobrucellosis, spondylitis, renal failure, hepatic failure, or a history of treatment for brucellosis in the last 6 months | NR | 9 | 10 | 19 | 8 |
|  | DS |  |  |  | 3 | 3 | 6 | 12 |
|  | RO |  |  |  | 5 | 4 | 9 | 12 |
| Hassan 2022[w17] | DR | A positive STAT result more than or equal to 1/160 association with compatible clinical findings | Complicated localized brucellosis (spondylitis, endocarditis, and meningoencephalitis), age less than 18 years, pregnancy, receiving antibiotics for more than 1 week before enrollment, and having COVID-19 | NR | 2 | 1 | 3 | 21 |
|  | D+TMP/SMX |  |  |  | 3 | 2 | 5 | 20 |
| Hassanjani Roushan 2010[w18] | DS | Patients aged >= 10 years and were diagnosed of brucellosis using standard tube agglutination (STA) titre >= 1:320 and 2-mercaptoethanol (2ME) titre >= 1:160, together with compatible clinical findings (fever, sweating, arthralgias, peripheral arthritis, sacroiliitis and epididymo-orchitis) | Spondylitis, endocarditis, neurobrucellosis and pregnant women | NR | 5 | 4 | 9 | 18 |
|  | DG |  |  |  | 2 | 2 | 4 | 23 |
| Jiang 2020[w19] | DRL | Brucellosis diagnosis and treatment guidelines are met, with varying degrees of malaise, excessive sweating, fever, joint pain, etc. occurring within 6 months. Symptoms of brucellosis, Brucella complement binding test titer >= 1:10, no previous treatment for brucellosis | Suffer from diseases that can cause fever, hepatosplenomegaly, and joint pain. Exclude patients who are allergic to the study drug and those who are pregnant or breastfeeding | 4.42 ± 1.05 | 3 | NR | 3 | 5 |
|  | DR |  |  | 5.74 ± 1.63 | 10 |  | 10 | 13 |
| Ju 2022[w20] | R+TC | Patients who meet diagnostic criteria for brucellosis and are under 64 years of age | Allergy to the drugs used in this study; treatment within 7 days; psychological or systemic illness; poor compliance | 1.79 ± 0.39 | NR | 2 | 2 | 4 |
|  | R |  |  | 2.84 ± 0.55 |  | 16 | 16 | 9 |
| Kalo 1996[w21] | DR | Positive serology and/or isolation of a Brucella sp. from blood in the presence of compatible epidemiological and clinical findings with wright seroagglutination assay titers equal to or higher than 1/160 and/or indirect immunofluorescence assay titers higher than 1/100 | Received antimicrobial therapy prior to the study, patients allergic to the drugs employed, children, under 15 years of age and pregnant women | 5 | 1 | NR | 1 | NR |
|  | D+CIP |  |  | 4 | 1 |  | 1 |  |
| Karabay 2004[w22] | DR | The presence of signs and symptoms compatible with brucellosis including a positive agglutination titre (>=1/160) and/or a positive culture | History of seizure, pregnancy and age under 15 years | 4.4 ± 1.1 (range 2-10.9) | 2 | NR | 2 | 4 |
|  | RO |  |  | 3.1 ± 1.3 (range 2-9) | 2 |  | 2 | 3 |
| Keramat 2009[w23] | DR | Clinical signs and symptoms compatible with acute brucellosis (duration<3 months) together with a standard tube agglutination test (STAT) titer of antibodies to brucella >= 1/160 and 2-mercaptoethanol (2-ME) >= 1/80 and/or a positive blood culture | Patients who were pregnant, aged under 17 years, had meningitis, neurobrucellosis, endocarditis, renal failure or hepatic failure | NR | 2 | 2 | 4 | 10 |
|  | R+CIP |  |  |  | 3 | 3 | 6 | 4 |
|  | D+CIP |  |  |  | 7 | 7 | 14 | 9 |
| Lang 1990[w24] | DR | Patients over 18 years old who presented with a four-fold rise in quantitative haemagglutination titres, with or without positive cultures for B. melitensis from blood, bone marrow or other body fluids | NR | 3.8 (3-4) | 0 | 0 | 0 | 0 |
|  | CIP |  |  | 5 (2-7) | 5 | 0 | 5 | 0 |
| Lang 1992[w25] | DS | Patients 6 years of age and older who presented with clinical signs suggestive of brucellosis (a temperature >38°C for at least 72 hours, arthralgia, back pain, rigors, and night sweats); a fourfold increase in titers of brucella antibody between collection of acute-phase and convalescent-phase sera and/or positive cul- tures ofblood, bone marrow, or other sterile fluids | NR | NR | 0 | 0 | 0 | 0 |
|  | CEF |  |  |  | 1 | 6 | 7 | 0 |
| Liu 2018[w26] | DR | Positive agglutination test results, a clear history of epidemiologic exposure, duration of illness <3 months, and voluntary informed consent | Those with liver and kidney impairment, malaria, cholera, tuberculosis, autoimmune diseases, combined viral hepatitis, Alcoholic hepatitis and drug-type liver injury and other diseases | 6.12 ± 0.64 | NR | 1 | 1 | NR |
|  | D |  |  | 9.21 ± 3.62 |  | 6 | 6 |  |
| Liu 2019[w27] | D+R+TMP/SMX | Positive Brucella agglutination test, two or more symptoms such as joint pain, weakness, sweating; age 16-70 years; no history of drug allergies | Patients with fever and hepatosplenomegaly; pregnant and breastfeeding women; patients with rheumatic fever and rheumatoid arthritis; patients with poor medical records; patients with tuberculosis, sepsis, and osteofluorosis | NR | NR | 0 | 0 | 10 |
|  | DR |  |  |  |  | 7 | 7 | 10 |
| Montejo 1993b[w28] | DR | >= 14 years of age, a clinical picture consistent with a diagnosis of brucellosis, isolation of germs from clinical specimens, and/ or titers of the standard tube agglutination test (STAT) of 1/160 | Antecedents of brucellosis in the previous year, the presence of serious associated illness, pregnancy, a reported allergy to one or more of the antimicrobial agents used in this study, and a diagnosis of endocarditis, spondylitis, or affection of the CNS by Brucella | NR | 5 | 1 | 6 | NR |
|  | DS |  |  |  | 1 | 0 | 1 |  |
|  | DS |  |  |  | 3 | 0 | 3 |  |
| Qian 2008[w29] | DL | Meets the diagnostic criteria for brucellosis, with typical symptoms such as fever, malaise, excessive sweating, joint pain, and a history of epidemiologic exposure | NR | NR | 0 | 0 | 0 | 4 |
|  | S+TMP/SMX |  |  |  | 0 | 0 | 0 | 11 |
| Qian 2009[w30] | DL | Fulfills the diagnostic criteria for brucellosis, no liver or kidney impairment | NR | NR | 0 | NR | 0 | 3 |
|  | S+TET |  |  |  | 0 |  | 0 | 7 |
| Ranjbar 2007[w31] | DRA | (1) Brucellosis clinical features including fever, sweats, arthralgia, hepatomegaly, splenomegaly, and/or signs of focal disease with a >= 1/160 standard tube agglutination titer of antibodies to Brucella; or (2) a tissue sample or blood culture positive for Brucella bacteria; or (3) a four-fold increase in Wright titer in a two-week interval with compatible clinical findings | Pregnant women, children under eight years of age, and patients with endocarditis and neurobrucellosis | NR | 6 | NR | 6 | 6 |
|  | DR |  |  |  | 9 |  | 9 | 4 |
| Roushan 2004[w32] | D+TMP/SMX | The finding of >=1/320 standard tube agglutination titer (STAT) of antibodies to brucella with a 2 mercaptoethanol (2 ME) >=1/160, in association with compatible clinical findings | Age less than 10 years, pregnancy, spondylitis, endocarditis, meningoencephalitis, previous history of brucellosis, and antimicrobial therapy for more than 7 days before enrollment | NR | 12 | 10 | 22 | 2 |
|  | R+TMP/SMX |  |  |  | 14 | 23 | 37 | 7 |
| Sarmadian 2009[w33] | D+R | Patient over 13yrs age, with brucellosis | NR | NR | NR | 7 | 7 | 2 |
|  | D+CIP |  |  |  |  | 9 | 9 | 1 |
| Sha 2017[w34] | DRL | Meets diagnostic criteria for brucellosis | Diseases that can cause fever and hepatosplenomegaly, such as typhoid fever, paratyphoid fever, etc. and diseases that can cause fever and wandering arthralgia | NR | 1 | 1 | 2 | 5 |
|  | DR |  |  |  | 7 | 7 | 14 | 19 |
| Sun 2015[w35] | DRL | NR | NR | NR | NR | 2 | 2 | NR |
|  | R |  |  |  |  | 6 | 6 |  |
| Sun 2020[w36] | DRL | Recent onset of symptoms such as malaise, excessive sweating, and fever that meet the diagnostic criteria for brucellosis; clear consciousness and normal cognition | Combination of malignant tumors; combination of psychiatric disorders; severe organ dysfunction; combination of immune and hematologic disorders | NR | 1 | 2 | 3 | 8 |
|  | DR |  |  |  | 3 | 10 | 13 | 4 |
| Sun 2023[w37] | DR | Signed informed consent patients aged 18 years or older who meet the diagnostic criteria for brucellosis and who present with symptoms within 6 months and have not been previously treated for brucellosis | With cardiopulmonary, hepatic and renal function serious abnormalities, allergies, serious mental illness, pregnant or lactating women | 4.89 ± 1.54 | NR | 8 | 8 | 11 |
|  | R+M |  |  | 5.74 ± 1.72 |  | 1 | 1 | 4 |
| Wang 2020[w38] | DL | NR | NR | NR | NR | 1 | 1 | 4 |
|  | L |  |  |  |  | 6 | 6 | 4 |
| Wang 2022[w39] | DL | Meets diagnostic criteria for brucellosis disease; clinically complete patient; history of epidemiologic exposure present | Comorbidity with severe mental disorders, oncologic diseases, organic diseases, speech and communication disorders, incomplete clinical data, and patients who withdrew from the study | NR | NR | 1 | 1 | 1 |
|  | L |  |  |  |  | 9 | 9 | 4 |
| Yin 2015[w40] | DRL | Patients presenting with fever, excessive sweating, and malaise within 6 months without serious complications such as meningitis, myocarditis, or osteoarthritis, with a Brucellosis complement binding test titer of >=1:10, and not receiving any prior related therapy | Pregnant women or patients younger than 16 | 4.5 ± 1.9 | 0 | NR | 0 | 9 |
|  | DR |  |  | 5.7 ± 2.0 | 4 |  | 4 | 7 |
| Zhang 2022[w41] | DRL | Meet the clinical diagnostic criteria of brucellosis; Have positive culture of Brucella; with different degrees of temperature rise symptoms such as high body temperature, fatigue, excessive sweating, joint swelling, and so on | Combined hepatic, renal, cardiac, cerebrovascular and other serious systemic diseases; allergic to the study drug; poor compliance, can’t cooperate; follow-up information is not complete | 1.83 ± 0.35 | 1 | 5 | 6 | 2 |
|  | DR |  |  | 2.89 ± 0.50 | 5 | 10 | 15 | 3 |
| Zhao 2023[w42] | R+TC+L | Laboratory tests confirmed the diagnosis of brucellosis; all were accompanied by varying degrees of fever, dizziness, headache, limb weakness and joint swelling; good cognitive status and compliance; informed and signed consent form for the study | Combined liver, kidney and other organ dysfunction or systemic infectious disease; 2) Fever and other symptoms due to other pathological factors; 3) Mental illness (3) Mental illness or inability to cooperate with the study; (4) Allergy to the drugs used in the study; (5) Withdrawal from the study or termination of the study due to unavoidable factors | 2.11 ± 0.41 | 1 | 2 | 3 | 4 |
|  | R+TC |  |  | 2.54 ± 0.50 | 7 | 11 | 18 | 2 |
| Zhou 2016[w43] | DRL | Meets diagnostic criteria for brucellosis, including epidemiologic history, clinical presentation, and laboratory tests | Diseases that can cause fever and hepatosplenomegaly, such as typhoid fever, paratyphoid fever, etc. and diseases that can cause fever and wandering arthralgia | 2.63 ± 0.74 | 2 | 0 | 2 | 4 |
|  | DR |  |  | 3.50 ± 0.97 | 10 | 0 | 10 | 3 |
