# Supplementary material for: Updated therapeutic options for human brucellosis: A systematic review and network meta-analysis of randomized controlled trials
Source: PLoS Negl Trop Dis. 2024 Aug 22;18(8):e0012405. doi: 10.1371/journal.pntd.0012405 (PMC11340890; doi:10.1371/journal.pntd.0012405)
Supplement: S7 Table — (DOCX) [file pntd.0012405.s007.docx]

**S7 Table**. List of excluded studies

1. Alp 2006

Alp E, Koc RK, Durak AC, et al. Doxycycline plus streptomycin versus ciprofloxacin plus rifampicin in spinal brucellosis [ISRCTN31053647]. BMC Infect Dis. 2006;6:72. Published 2006 Apr 11. doi:10.1186/1471-2334-6-72

Reason for exclusion: No outcomes of interested

2. Ariza 1985a

Ariza J, Gudiol F, Pallarés R, Rufí G, Fernández-Viladrich P. Comparative trial of co-trimoxazole versus tetracycline-streptomycin in treating human brucellosis. J Infect Dis. 1985;152(6):1358-1359. doi:10.1093/infdis/152.6.1358

Reason for exclusion: Unclear treatment options

3. Ariza 1985b

Ariza J, Gudiol F, Pallarés R, Rufí G, Fernández-Viladrich P. Comparative trial of rifampin-doxycycline versus tetracycline-streptomycin in the therapy of human brucellosis. Antimicrob Agents Chemother. 1985;28(4):548-551. doi:10.1128/AAC.28.4.548

Reason for exclusion: Quazi-randomized

4. Feiz 1973

Feiz JM, Sabbaghian H, Sohrabi F. A comparative study of therapeutic agents used for treatment of acute brucellosis. Br J Clin Pract. 1973;27(11):410-413.

Reason for exclusion: Quazi-randomized

5. Irmak 2003

Irmak H, Buzgan T, Karahocagil MK, Evirgen O, Akdeniz H, Demiröz AP. The effect of levamisole combined with the classical treatment in chronic brucellosis. Tohoku J Exp Med. 2003;201(4):221-228. doi:10.1620/tjem.201.221

Reason for exclusion: Interventions include non-antibiotic drugs

6. Karami 2020

Karami, Afsaneh, et al. "Effect of 8-week and 12-week triple therapy (doxycycline, rifampicin, and gentamicin) on brucellosis: A comparative study." Journal of Acute Disease 9.4 (2020): 161-165.

Reason for exclusion: Interventions are same combination of drugs

7. Khuri-Bulos 1993

Khuri-Bulos NA, Daoud AH, Azab SM. Treatment of childhood brucellosis: results of a prospective trial on 113 children. Pediatr Infect Dis J. 1993;12(5):377-381. doi:10.1097/00006454-199305000-00005

Reason for exclusion: Non-randomized

8. Liang 2018

Chao L, Jing L. Effect of different antibacterial drug combination regimens on the treatment effect, recurrence rate and occurrence of adverse effects in brucellosis. Strait Pharm J. 2018;30(11):221–2. (in Chinese).

Reason for exclusion: Quazi-randomized

9. Lubani 1989

Lubani MM, Dudin KI, Sharda DC, et al. A multicenter therapeutic study of 1100 children with brucellosis. Pediatr Infect Dis J. 1989;8(2):75-78.

Reason for exclusion: Non-randomized

10. Majzoobi 2018

Majzoobi MM, Hashemi SH, Mamani M, Keramat F, Poorolajal J, Ghasemi Basir HR. Effect of hydroxychloroquine on treatment and recurrence of acute brucellosis: a single-blind, randomized clinical trial. Int J Antimicrob Agents. 2018;51(3):365-369. doi:10.1016/j.ijantimicag.2017.08.009

Reason for exclusion: Interventions include non-antibiotic drugs

11. Majzoobi 2022

Majzoobi MM, Hashmi SH, Emami K, Soltanian AR. Combination of doxycycline, streptomycin and hydroxychloroquine for short-course treatment of brucellosis: a single-blind randomized clinical trial. Infection. 2022;50(5):1267-1271. doi:10.1007/s15010-022-01806-x

Reason for exclusion: Interventions include non-antibiotic drugs

12. Mile 2012

Mile B, Valerija K, Krsto G, Ivan V, Ilir D, Nikola L. Doxycycline-rifampin versus doxycycline-rifampin-gentamicin in treatment of human brucellosis. Trop Doct. 2012;42(1):13-17. doi:10.1258/td.2011.110284

Reason for exclusion: Non-randomized

13. Montejo 1993a

Montejo JM, Alberola I, Glez-Zarate P, et al. Open, randomized therapeutic trial of six antimicrobial regimens in the treatment of human brucellosis. Clin Infect Dis. 1993;16(5):671-676. doi:10.1093/clind/16.5.671

Reason for exclusion: Interventions are same combination of drugs

14. Rodriguez Zapata 1987

Rodriguez Zapata M, Gamo Herranz A, De La Morena Fernández J. Comparative study of two regimens in the treatment of brucellosis. Chemioterapia. 1987;6(2 Suppl):360-362.

Reason for exclusion: No full text

15. Salehi 2023

Salehi M, Farbod F, Khalili H, Rahmani H, Jafari S, Abbasi A. Comparing efficacy and safety of high-dose and standard-dose rifampicin in the treatment of brucellosis: a randomized clinical trial. J Antimicrob Chemother. 2023;78(4):1084-1091. doi:10.1093/jac/dkad051

Reason for exclusion: Interventions are same combination of drugs

16. Shen 2021

Licheng S. Analysis of the efficacy of drug therapy alone for patients with atypical Brucella osteoarthritis. China Prac Med. 2021;16(31):128–130. (in Chinese). doi: 10.14163/j.cnki.11-5547/r.2021.31.047

Reason for exclusion: Uncontrolled patient treatment

17. Sofian 2014

Sofian M, Velayati AA, Aghakhani A, et al. Comparison of two durations of triple-drug therapy in patients with uncomplicated brucellosis: A randomized controlled trial. Scand J Infect Dis. 2014;46(8):573-577. doi:10.3109/00365548.2014.918275

Reason for exclusion: Interventions are same combination of drugs

18. Solera 1995

Solera J, Rodríguez-Zapata M, Geijo P, et al. Doxycycline-rifampin versus doxycycline-streptomycin in treatment of human brucellosis due to Brucella melitensis. The GECMEI Group. Grupo de Estudio de Castilla-la Mancha de Enfermedades Infecciosas. Antimicrob Agents Chemother. 1995;39(9):2061-2067. doi:10.1128/AAC.39.9.2061

Reason for exclusion: Quazi-randomized

19. Solera 2004

Solera J, Geijo P, Largo J, et al. A randomized, double-blind study to assess the optimal duration of doxycycline treatment for human brucellosis. Clin Infect Dis. 2004;39(12):1776-1782. doi:10.1086/426024

Reason for exclusion: Interventions are same combination of drugs

20. Taghvaee 2011

Taghvaee MR, Nozadi MS, Hassani M. A comparison between doxycycline-rifampin and ciprofloxacin-rifampin regimens in the treatment of acute Brucellosis. Indian J Med Sci. 2011;65(10):436-443.

Reason for exclusion: Non-randomized

21. Yang 2021

Yang XM, Jia YL, Zhang Y, et al. Clinical Effect of Doxycycline Combined with Compound Sulfamethoxazole and Rifampicin in the Treatment of Brucellosis Spondylitis. Drug Des Devel Ther. 2021;15:4733-4740. Published 2021 Nov 23. doi:10.2147/DDDT.S341242

Reason for exclusion: A retrospective study
