# Supplementary material for: Updated therapeutic options for human brucellosis: A systematic review and network meta-analysis of randomized controlled trials
Source: PLoS Negl Trop Dis. 2024 Aug 22;18(8):e0012405. doi: 10.1371/journal.pntd.0012405 (PMC11340890; doi:10.1371/journal.pntd.0012405)
Supplement: S8 Table — (DOCX) [file pntd.0012405.s008.docx]

**S8 Table**. Details on risk of bias and certainty of evidence assessments

**1.1 Assessment of risk of bias**

We assessed risk of bias for the primary outcome, including overall failure and side effects. Ratings assessing the risk of bias were conducted following the guidelines outlined in the Cochrane Risk of Bias tool 2 [1]. The guidelines cover the following five areas: randomisation process, deviations from intended interventions, missing outcome data, measurement of the outcome, and selection of reported results. In order to ensure the accuracy of double-checking, we have pre-established the following evaluation benchmarks.

| Domain 1: Randomisation process  Points: random sequence generation, allocation concealment, and baseline characteristics |
| --- |
| In order to be categorized as having a low risk of bias, studies needed to (1) adopt a random component to generate sequence of randomisation, e.g., coin-tossing, throwing dice,  or by using randomization table; (2) offer a precise explanation of allocation sequence concealment, such as central randomization conducted by a third party or the utilization of sealed, opaque envelopes; (3) there was no difference in baseline characteristics, specifically expressed in the text as P < 0.05. Studies which only provided vague descriptions, e.g., ‘randomised’, ‘blind method was uesd’ were rated at some concerns for risk of bias. If there was a partial difference in baseline characteristics, we also evaluated it as some concerns for risk of bias. In other cases we rated the risk of bias as high. |

| Domain 2: Deviations from intended interventions  Points: blinding of participants and carers and impact of deviations on outcome |
| --- |
| In order to be categorized as having a low risk of bias, studies needed to (1) participants and carers were unaware of treatment measures or therapy trials that were described as double or triple blind; (2) data analysis adhering to intention-to-treat (ITT) principles. We used the threshold of 15% of excluded participants for inappropriate reasons to determine if the study was at high (≥ 15%) or some concerns (< 15%) of risk of bias. This study was a parallel comparison between drugs and most of the studies used a combination of different classes of drugs with oral or injectable routes of administration, so we considered blinding to have less impact on the study, and therefore we used a larger tolerance for this part of the evaluation. |

| Domain 3: Missing outcome data  Points: missing outcome measures and loss to follow-up |
| --- |
| In order to be categorized as having a low risk of bias, studies needed to (1) trials in which missing outcome data (including outcome data that has been imputed) < 15%; (2) Despite the presence of missing data, the authors report specific causes of shedding and individual data related to the outcome data. If the missing data were between 15% to 25% and the article did not report data related to treatment failure and side effects, such a risk of bias was evaluated as some concerns. Trials with missing outcome data, including imputed data, greater than 25%, where the missing data is likely associated with the true outcome or there are discrepancies in the quantity or reasons for missing data across intervention groups. |

| Domain 4: Measurement of outcome  Points: blinding of outcome adjudicators and objectivity of outcome |
| --- |
| In order to be categorized as having a low risk of bias, studies needed to (1) trials where patients are unaware of the intervention and outcomes are reported by the patients themselves; (2) Trials that are described as double or triple blind. When studies only provided ambiguous descriptions of blinding, they were assessed with some concerns regarding the risk of bias. Trials in which outcome adjudicators are not blind and the outcomes are not objective would be assessed with high risk of bias. |

| Domain 5: Selection of reported results  Points: selective reporting of outcome measures |
| --- |
| We looked for pre-registered reports (e.g., protocols in journals, clinical trial registries) which could corroborate that relapse, therapeutic failure or sides effects was originally planned as the primary outcome for brucellosis. Since the overall failure of our primary outcome was defined as the sum of relapse and therapeutic failure, if a study was unregistered, we rated it as some concerns of risk as long as it reported one of the two indicators above. When results for outcomes that were analyzed and reported, but were not originally specified in a statistical analysis plan or protocol, and where the timing of reporting does not align with other outcomes in the trial report, or there are other indications suggesting selective reporting, we assessed it as high risk of bias. |

**1.2 Assessment of certainty of evidence**

We evaluated the confidence in estimates of the primary outcomes in the findings from the network meta-analysis with the Confidence In Network Meta-Analysis (CINeMA) framework implemented in semi-automated method through the web application: https://cinema.ispm.unibe.ch/, as well as the Grading of Recommendations Assessment, Development and Evaluation (GRADE) framework. The following six domains were assessed: within-study bias, reporting bias, indirectness, imprecision, heterogeneity, and incoherence [2].

| Domain 1: Within-study bias |
| --- |
| The overall risk of bias ratings from the Cochrane Risk of Bias tool, as described previously, were employed. In all network meta-analyses, the weighted average of within-study bias was utilized. |

| Domain 2: Reporting bias |
| --- |
| We selected all low risk. We plotted comparison-adjusted funnel plots (provided below), and we were unable to detect asymmetry at visual inspection. |

| Domain 3: Indirectness |
| --- |
| We used eligibility criteria to ensure that the selected studies were all directly relevant to the research questions. As described in the main text, although there was a small proportion of brucellosis of spondylitis in our study, which may be considered somewhat indirect; however, we assumed that the grouping of such patients in a randomized controlled trial would be randomized and balanced. For all network meta-analyses, the weighted average of indirectness bias was used. |

| Domain 4: Imprecision |
| --- |
| For binary data, we defined the clinically important size of effect as a Relative risk (RR) of 1, indicating that relative effect estimates below and above 1.000 are considered clinically important. In clinical research, the clinical thresholds for RR are typically 0.8 or 1.25 because these values reflect clinically significant differences. When the RR is less than 0.8, it indicates a 20% or greater reduction in risk in the treatment group compared to the control group, which is considered clinically important. Conversely, when the RR is greater than 1.25, it suggests a 25% or greater increase in risk in the treatment group compared to the control group, which is also deemed clinically significant. These thresholds aid in assessing the clinical relevance and significance of study findings. |

| Domain 5: Heterogeneity |
| --- |
| The assessment of heterogeneity relied on the associated p-value, confidence interval, range of equivalence, and prediction interval. We judged an effect size of 0.8 or 1.25 as clinically relevant for dichotomous outcomes. |

| Domain 6: Incoherence |
| --- |
| See above as heterogeneity |

**References**

1. Sterne JAC, Savović J, Page MJ, et al. RoB 2: a revised tool for assessing risk of bias in randomised trials. BMJ. 2019;366:l4898. Published 2019 Aug 28. doi:10.1136/bmj.l4898

2. Cumpston M, Li T, Page MJ, et al. Updated guidance for trusted systematic reviews: a new edition of the Cochrane Handbook for Systematic Reviews of Interventions. Cochrane Database Syst Rev. 2019;10(10):ED000142. doi:10.1002/14651858.ED000142
