# Supplementary material for: Updated therapeutic options for human brucellosis: A systematic review and network meta-analysis of randomized controlled trials
Source: PLoS Negl Trop Dis. 2024 Aug 22;18(8):e0012405. doi: 10.1371/journal.pntd.0012405 (PMC11340890; doi:10.1371/journal.pntd.0012405)
Supplement: S9 Table — (DOCX) [file pntd.0012405.s009.docx]

**S9 Table**. Risk of bias of included studies

**1. Overall failure**

| Study | Randomisation process | Deviations from intended interventions | Missing outcome data | Measurement of outcome | Selection of reported results |
| --- | --- | --- | --- | --- | --- |
| Acocella 1989a | High risk | Some concerns | Low risk | Low risk | Low risk |
| Acocella 1989b | High risk | High risk | Low risk | Low risk | Low risk |
| Acocella 1989c | High risk | High risk | Low risk | Low risk | Low risk |
| Agalar 1999 | Low risk | Low risk | Low risk | Low risk | Low risk |
| Akova 1993 | Low risk | Low risk | Low risk | Low risk | Low risk |
| Alavi 2007 | Low risk | Low risk | Low risk | Low risk | Low risk |
| Ariza 1992 | Low risk | Low risk | Low risk | Low risk | Low risk |
| Buzon 1982 | Low risk | Low risk | Low risk | Some concerns | Some concerns |
| Chai 2018 | Low risk | Some concerns | Low risk | Some concerns | Some concerns |
| Chen 2016 | Some concerns | High risk | Low risk | Some concerns | Some concerns |
| Colmenero 1989 | Low risk | Low risk | Low risk | Low risk | Low risk |
| Colmenero 1994 | Low risk | Low risk | Low risk | Some concerns | Low risk |
| Deng 2016 | Some concerns | High risk | Low risk | High risk | Some concerns |
| Ersoy 2005a | Low risk | Some concerns | Low risk | Low risk | Low risk |
| Ersoy 2005b | Low risk | Some concerns | Low risk | Low risk | Low risk |
| Ersoy 2005c | Low risk | Some concerns | Low risk | Low risk | Low risk |
| Guo 2023 | Some concerns | High risk | Low risk | Some concerns | Some concerns |
| Hasanain 2016 | Low risk | Low risk | Low risk | Low risk | Low risk |
| Hasanjani Roushan 2006 | Low risk | Low risk | Low risk | Low risk | Low risk |
| Hashemi 2011a | Low risk | Low risk | Low risk | Low risk | Low risk |
| Hashemi 2011b | Low risk | Low risk | Low risk | Low risk | Low risk |
| Hashemi 2011c | Low risk | Low risk | Low risk | Low risk | Low risk |
| Hassan 2022 | Low risk | Low risk | Low risk | Low risk | Low risk |
| Hassanjani Roushan 2010 | Low risk | Low risk | Low risk | Low risk | Low risk |
| Jiang 2020 | Low risk | Low risk | Low risk | Some concerns | Some concerns |
| Ju 2022 | Some concerns | Low risk | Low risk | Some concerns | Some concerns |
| Kalo 1996 | High risk | Low risk | Low risk | High risk | Some concerns |
| Karabay 2004 | Low risk | Some concerns | Low risk | Some concerns | Some concerns |
| Keramat 2009a | Low risk | Some concerns | High risk | Low risk | Low risk |
| Keramat 2009b | Low risk | Some concerns | High risk | Low risk | Low risk |
| Keramat 2009c | Low risk | Some concerns | High risk | Low risk | Low risk |
| Lang 1990 | Low risk | Low risk | Low risk | Low risk | Low risk |
| Lang 1992 | Low risk | Low risk | High risk | Low risk | Low risk |
| Liu 2018 | Low risk | Low risk | Low risk | High risk | Some concerns |
| Liu 2019 | Low risk | Low risk | Low risk | Some concerns | Some concerns |
| Montejo 1993b | Low risk | Low risk | Low risk | Some concerns | Low risk |
| Montejo 1993c | Low risk | Low risk | Low risk | Some concerns | Low risk |
| Qian 2008 | Low risk | Low risk | High risk | Low risk | Low risk |
| Qian 2009 | Some concerns | High risk | Low risk | Some concerns | Some concerns |
| Ranjbar 2007 | Low risk | Low risk | Low risk | Low risk | Low risk |
| Roushan 2004 | Low risk | Low risk | Low risk | Low risk | Low risk |
| Sarmadian 2009 | Low risk | Low risk | Low risk | Low risk | Low risk |
| Sha 2017 | Low risk | Low risk | Low risk | Low risk | Low risk |
| Sun 2015 | Some concerns | High risk | Low risk | High risk | Some concerns |
| Sun 2020 | Some concerns | Some concerns | Low risk | Low risk | Low risk |
| Sun 2023 | Low risk | Low risk | Low risk | Some concerns | Some concerns |
| Wang 2020 | Some concerns | Some concerns | High risk | Some concerns | Some concerns |
| Wang 2022 | Low risk | Low risk | High risk | Some concerns | Some concerns |
| Yin 2015 | Low risk | Low risk | Low risk | Some concerns | Some concerns |
| Zhang 2022 | Low risk | Low risk | Low risk | Low risk | Low risk |
| Zhao 2023 | Low risk | Low risk | Low risk | Low risk | Low risk |
| Zhou 2016 | Low risk | Low risk | Low risk | Low risk | Low risk |

**2. Side effects**

| Study | Randomisation process | Deviations from intended interventions | Missing outcome data | Measurement of outcome | Selection of reported results |
| --- | --- | --- | --- | --- | --- |
| Acocella 1989a | High risk | Some concerns | Low risk | Low risk | Low risk |
| Acocella 1989b | High risk | High risk | Low risk | Low risk | Low risk |
| Acocella 1989c | High risk | High risk | Low risk | Low risk | Low risk |
| Agalar 1999 | Low risk | Low risk | Low risk | Low risk | Low risk |
| Akova 1993 | Low risk | Low risk | Low risk | Low risk | Low risk |
| Alavi 2007 | Low risk | Low risk | Low risk | Low risk | Low risk |
| Ariza 1992 | Low risk | Low risk | Low risk | Low risk | Low risk |
| Buzon 1982 | Low risk | Low risk | Low risk | Some concerns | Some concerns |
| Chai 2018 | Low risk | Some concerns | Low risk | Some concerns | Some concerns |
| Chen 2016 | Some concerns | High risk | Low risk | Some concerns | Some concerns |
| Colmenero 1989 | Low risk | Low risk | Low risk | Low risk | Low risk |
| Ersoy 2005a | Low risk | Some concerns | Low risk | Low risk | Low risk |
| Ersoy 2005b | Low risk | Some concerns | Low risk | Low risk | Low risk |
| Ersoy 2005c | Low risk | Some concerns | Low risk | Low risk | Low risk |
| Guo 2023 | Some concerns | High risk | Low risk | Some concerns | Some concerns |
| Hasanain 2016 | Low risk | Low risk | Low risk | Low risk | Low risk |
| Hasanjani Roushan 2006 | Low risk | Low risk | Low risk | Low risk | Low risk |
| Hashemi 2011a | Low risk | Low risk | Low risk | Low risk | Low risk |
| Hashemi 2011b | Low risk | Low risk | Low risk | Low risk | Low risk |
| Hashemi 2011c | Low risk | Low risk | Low risk | Low risk | Low risk |
| Hassan 2022 | Low risk | Low risk | Low risk | Low risk | Low risk |
| Hassanjani Roushan 2010 | Low risk | Low risk | Low risk | Low risk | Low risk |
| Jiang 2020 | Low risk | Low risk | Low risk | Some concerns | Some concerns |
| Ju 2022 | Some concerns | Low risk | Low risk | Some concerns | Some concerns |
| Karabay 2004 | Low risk | Some concerns | Low risk | Some concerns | Some concerns |
| Keramat 2009a | Low risk | Some concerns | High risk | Low risk | Low risk |
| Keramat 2009b | Low risk | Some concerns | High risk | Low risk | Low risk |
| Keramat 2009c | Low risk | Some concerns | High risk | Low risk | Low risk |
| Lang 1990 | Low risk | Low risk | Low risk | Low risk | Low risk |
| Lang 1992 | Low risk | Low risk | High risk | Low risk | Low risk |
| Liu 2019 | Low risk | Low risk | Low risk | Some concerns | Some concerns |
| Qian 2008 | Low risk | Low risk | Low risk | Low risk | Low risk |
| Qian 2009 | Some concerns | High risk | Low risk | Some concerns | Some concerns |
| Ranjbar 2007 | Low risk | Low risk | Low risk | Low risk | Low risk |
| Roushan 2004 | Low risk | Low risk | Low risk | Low risk | Low risk |
| Sarmadian 2009 | Low risk | Low risk | Low risk | Low risk | Low risk |
| Sha 2017 | Low risk | Low risk | Low risk | Low risk | Low risk |
| Sun 2020 | Some concerns | Some concerns | Low risk | Low risk | Low risk |
| Sun 2023 | Low risk | Low risk | Low risk | Some concerns | Some concerns |
| Wang 2020 | Some concerns | Some concerns | High risk | Some concerns | Some concerns |
| Wang 2022 | Low risk | Low risk | High risk | Some concerns | Some concerns |
| Yin 2015 | Low risk | Low risk | Low risk | Some concerns | Some concerns |
| Zhang 2022 | Low risk | Low risk | Low risk | Low risk | Low risk |
| Zhao 2023 | Low risk | Low risk | Low risk | Low risk | Low risk |
| Zhou 2016 | Low risk | Low risk | Low risk | Low risk | Low risk |
