# Supplementary material for: Updated therapeutic options for human brucellosis: A systematic review and network meta-analysis of randomized controlled trials
Source: PLoS Negl Trop Dis. 2024 Aug 22;18(8):e0012405. doi: 10.1371/journal.pntd.0012405 (PMC11340890; doi:10.1371/journal.pntd.0012405)
Supplement: S10 Table — (DOCX) [file pntd.0012405.s010.docx]

**S10 Table**. Assessments of certainty of evidence of primary outcomes using CINeMA

**1. Overall failure**

| **Comparison** | **Within-study bias** | **Reporting bias** | **Indirectness** | **Imprecision** | **Heterogeneity** | **Incoherence** | **Confidence rating** |
| --- | --- | --- | --- | --- | --- | --- | --- |
| D+Quinolones:DR | Some concerns | Low risk | No concerns | Some concerns | No concerns | No concerns | Low |
| D+Quinolones:R+Quinolones | Some concerns | Low risk | No concerns | No concerns | No concerns | No concerns | Moderate |
| D+Quinolones:S+TMP/SMX | Major concerns | Low risk | No concerns | Major concerns | No concerns | No concerns | Very low |
| D+Quinolones:S+Tetracyclines | Some concerns | Low risk | No concerns | No concerns | No concerns | No concerns | Moderate |
| D+Quinolones:Single | Some concerns | Low risk | No concerns | No concerns | No concerns | No concerns | Moderate |
| DR:D+TMP/SMX | No concerns | Low risk | No concerns | Major concerns | No concerns | No concerns | Low |
| D+TMP/SMX:R+TMP/SMX | No concerns | Low risk | No concerns | No concerns | No concerns | No concerns | High |
| DG:DS | No concerns | Low risk | No concerns | No concerns | Some concerns | No concerns | Moderate |
| DR:DS | Some concerns | Low risk | No concerns | No concerns | No concerns | No concerns | Moderate |
| DR:R+Quinolones | Some concerns | Low risk | No concerns | Some concerns | No concerns | No concerns | Low |
| DR:R+Tetracyclines | Some concerns | Low risk | No concerns | Major concerns | No concerns | Major concerns | Very low |
| DR:S+Tetracyclines | Major concerns | Low risk | No concerns | No concerns | No concerns | No concerns | Low |
| DR:Single | Some concerns | Low risk | No concerns | No concerns | No concerns | No concerns | Moderate |
| DR:Triple | No concerns | Low risk | No concerns | No concerns | No concerns | No concerns | High |
| DS:R+Quinolones | Some concerns | Low risk | No concerns | Some concerns | No concerns | No concerns | Low |
| DS:S+Tetracyclines | Major concerns | Low risk | No concerns | No concerns | No concerns | No concerns | Low |
| DS:Single | Some concerns | Low risk | No concerns | No concerns | No concerns | No concerns | Moderate |
| R+Tetracyclines:Single | Some concerns | Low risk | No concerns | No concerns | No concerns | No concerns | Moderate |
| R+Tetracyclines:Triple | Some concerns | Low risk | No concerns | No concerns | No concerns | No concerns | Moderate |
| Single:Triple | Some concerns | Low risk | No concerns | No concerns | No concerns | Major concerns | Very low |
| D+Quinolones:D+TMP/SMX | Some concerns | Low risk | No concerns | Some concerns | No concerns | No concerns | Low |
| DG:D+Quinolones | Some concerns | Low risk | No concerns | No concerns | No concerns | No concerns | Moderate |
| D+Quinolones:DS | Some concerns | Low risk | No concerns | No concerns | No concerns | No concerns | Moderate |
| D+Quinolones:R+TMP/SMX | No concerns | Low risk | No concerns | Major concerns | No concerns | No concerns | Low |
| D+Quinolones:R+Tetracyclines | Some concerns | Low risk | No concerns | Major concerns | No concerns | No concerns | Very low |
| D+Quinolones:Triple | Some concerns | Low risk | No concerns | No concerns | No concerns | No concerns | Moderate |
| DG:D+TMP/SMX | No concerns | Low risk | No concerns | Some concerns | No concerns | No concerns | Moderate |
| DS:D+TMP/SMX | No concerns | Low risk | No concerns | Major concerns | No concerns | No concerns | Low |
| D+TMP/SMX:R+Quinolones | No concerns | Low risk | No concerns | Major concerns | No concerns | No concerns | Low |
| D+TMP/SMX:R+Tetracyclines | Some concerns | Low risk | No concerns | Major concerns | No concerns | No concerns | Very low |
| D+TMP/SMX:S+TMP/SMX | Some concerns | Low risk | No concerns | Major concerns | No concerns | No concerns | Very low |
| D+TMP/SMX:S+Tetracyclines | Some concerns | Low risk | No concerns | No concerns | No concerns | No concerns | Moderate |
| D+TMP/SMX:Single | Some concerns | Low risk | No concerns | No concerns | No concerns | No concerns | Moderate |
| D+TMP/SMX:Triple | No concerns | Low risk | No concerns | Major concerns | No concerns | No concerns | Low |
| DG:DR | No concerns | Low risk | No concerns | No concerns | No concerns | No concerns | High |
| DG:R+Quinolones | No concerns | Low risk | No concerns | No concerns | No concerns | No concerns | High |
| DG:R+TMP/SMX | No concerns | Low risk | No concerns | No concerns | No concerns | No concerns | High |
| DG:R+Tetracyclines | Some concerns | Low risk | No concerns | No concerns | No concerns | No concerns | Moderate |
| DG:S+TMP/SMX | Some concerns | Low risk | No concerns | Major concerns | No concerns | No concerns | Very low |
| DG:S+Tetracyclines | Some concerns | Low risk | No concerns | No concerns | No concerns | No concerns | Moderate |
| DG:Single | Some concerns | Low risk | No concerns | No concerns | No concerns | No concerns | Moderate |
| DG:Triple | No concerns | Low risk | No concerns | Major concerns | No concerns | No concerns | Low |
| DR:R+TMP/SMX | No concerns | Low risk | No concerns | Major concerns | No concerns | No concerns | Low |
| DR:S+TMP/SMX | Some concerns | Low risk | No concerns | Major concerns | No concerns | No concerns | Very low |
| DS:R+TMP/SMX | No concerns | Low risk | No concerns | Major concerns | No concerns | No concerns | Low |
| DS:R+Tetracyclines | Some concerns | Low risk | No concerns | No concerns | No concerns | No concerns | Moderate |
| DS:S+TMP/SMX | Some concerns | Low risk | No concerns | Major concerns | No concerns | No concerns | Very low |
| DS:Triple | No concerns | Low risk | No concerns | Major concerns | No concerns | No concerns | Low |
| R+Quinolones:R+TMP/SMX | No concerns | Low risk | No concerns | Major concerns | No concerns | No concerns | Low |
| R+Quinolones:R+Tetracyclines | Some concerns | Low risk | No concerns | Some concerns | No concerns | No concerns | Low |
| R+Quinolones:S+TMP/SMX | Some concerns | Low risk | No concerns | Major concerns | No concerns | No concerns | Very low |
| R+Quinolones:S+Tetracyclines | Some concerns | Low risk | No concerns | No concerns | No concerns | No concerns | Moderate |
| R+Quinolones:Single | Some concerns | Low risk | No concerns | No concerns | No concerns | No concerns | Moderate |
| R+Quinolones:Triple | Some concerns | Low risk | No concerns | No concerns | Some concerns | No concerns | Low |
| R+Tetracyclines:R+TMP/SMX | No concerns | Low risk | No concerns | Major concerns | No concerns | No concerns | Low |
| R+TMP/SMX:S+TMP/SMX | Some concerns | Low risk | No concerns | Major concerns | No concerns | No concerns | Very low |
| R+TMP/SMX:S+Tetracyclines | Some concerns | Low risk | No concerns | No concerns | No concerns | No concerns | Moderate |
| R+TMP/SMX:Single | Some concerns | Low risk | No concerns | No concerns | No concerns | No concerns | Moderate |
| R+TMP/SMX:Triple | No concerns | Low risk | No concerns | Some concerns | No concerns | No concerns | Moderate |
| R+Tetracyclines:S+TMP/SMX | Some concerns | Low risk | No concerns | Major concerns | No concerns | No concerns | Very low |
| R+Tetracyclines:S+Tetracyclines | Some concerns | Low risk | No concerns | No concerns | No concerns | No concerns | Moderate |
| S+Tetracyclines:S+TMP/SMX | Major concerns | Low risk | No concerns | Major concerns | No concerns | No concerns | Very low |
| Single:S+TMP/SMX | Major concerns | Low risk | No concerns | Major concerns | No concerns | No concerns | Very low |
| S+TMP/SMX:Triple | Some concerns | Low risk | No concerns | Major concerns | No concerns | No concerns | Very low |
| Single:S+Tetracyclines | Some concerns | Low risk | No concerns | Major concerns | No concerns | No concerns | Very low |
| S+Tetracyclines:Triple | Some concerns | Low risk | No concerns | No concerns | No concerns | No concerns | Moderate |

**2. Side effects**

| **Comparison** | **Within-study bias** | **Reporting bias** | **Indirectness** | **Imprecision** | **Heterogeneity** | **Incoherence** | **Confidence rating** |
| --- | --- | --- | --- | --- | --- | --- | --- |
| D+Quinolones:DR | Some concerns | Low risk | No concerns | Major concerns | No concerns | No concerns | Very low |
| D+Quinolones:R+Quinolones | Some concerns | Low risk | No concerns | Some concerns | Some concerns | No concerns | Very low |
| D+Quinolones:S+TMP/SMX | No concerns | Low risk | No concerns | Some concerns | Some concerns | No concerns | Low |
| D+Quinolones:S+Tetracyclines | Major concerns | Low risk | No concerns | Major concerns | No concerns | No concerns | Very low |
| D+Quinolones:Single | Major concerns | Low risk | No concerns | Major concerns | No concerns | No concerns | Very low |
| DR:D+TMP/SMX | No concerns | Low risk | No concerns | Major concerns | No concerns | No concerns | Low |
| D+TMP/SMX:R+TMP/SMX | No concerns | Low risk | No concerns | Major concerns | No concerns | No concerns | Low |
| DG:DS | No concerns | Low risk | No concerns | Major concerns | No concerns | No concerns | Low |
| DR:DS | No concerns | Low risk | No concerns | Some concerns | Some concerns | No concerns | Low |
| DR:R+Quinolones | Some concerns | Low risk | No concerns | No concerns | Some concerns | No concerns | Low |
| DR:R+Tetracyclines | Some concerns | Low risk | No concerns | No concerns | No concerns | No concerns | Moderate |
| DR:S+Tetracyclines | Major concerns | Low risk | No concerns | Major concerns | No concerns | No concerns | Very low |
| DR:Single | Some concerns | Low risk | No concerns | Major concerns | No concerns | No concerns | Very low |
| DR:Triple | Some concerns | Low risk | No concerns | Major concerns | No concerns | No concerns | Very low |
| DS:R+Quinolones | No concerns | Low risk | No concerns | Major concerns | No concerns | No concerns | Low |
| DS:S+Tetracyclines | Some concerns | Low risk | No concerns | Some concerns | No concerns | No concerns | Low |
| DS:Single | Some concerns | Low risk | No concerns | Major concerns | No concerns | No concerns | Very low |
| R+Tetracyclines:Single | Some concerns | Low risk | No concerns | No concerns | Some concerns | No concerns | Low |
| R+Tetracyclines:Triple | Some concerns | Low risk | No concerns | No concerns | Some concerns | No concerns | Low |
| D+Quinolones:D+TMP/SMX | Some concerns | Low risk | No concerns | Major concerns | No concerns | No concerns | Very low |
| DG:D+Quinolones | Some concerns | Low risk | No concerns | Major concerns | No concerns | No concerns | Very low |
| D+Quinolones:DS | Some concerns | Low risk | No concerns | Major concerns | No concerns | No concerns | Very low |
| D+Quinolones:R+TMP/SMX | Some concerns | Low risk | No concerns | Major concerns | No concerns | No concerns | Very low |
| D+Quinolones:R+Tetracyclines | Some concerns | Low risk | No concerns | No concerns | No concerns | No concerns | Moderate |
| D+Quinolones:Triple | Some concerns | Low risk | No concerns | Major concerns | No concerns | No concerns | Very low |
| DG:D+TMP/SMX | No concerns | Low risk | No concerns | Major concerns | No concerns | No concerns | Low |
| DS:D+TMP/SMX | No concerns | Low risk | No concerns | Major concerns | No concerns | No concerns | Low |
| D+TMP/SMX:R+Quinolones | No concerns | Low risk | No concerns | Some concerns | Some concerns | No concerns | Low |
| D+TMP/SMX:R+Tetracyclines | Some concerns | Low risk | No concerns | No concerns | Some concerns | No concerns | Low |
| D+TMP/SMX:S+TMP/SMX | Some concerns | Low risk | No concerns | Major concerns | No concerns | No concerns | Very low |
| D+TMP/SMX:S+Tetracyclines | Some concerns | Low risk | No concerns | Major concerns | No concerns | No concerns | Very low |
| D+TMP/SMX:Single | Some concerns | Low risk | No concerns | Major concerns | No concerns | No concerns | Very low |
| D+TMP/SMX:Triple | No concerns | Low risk | No concerns | Major concerns | No concerns | No concerns | Low |
| DG:DR | No concerns | Low risk | No concerns | Major concerns | No concerns | No concerns | Low |
| DG:R+Quinolones | No concerns | Low risk | No concerns | Major concerns | No concerns | No concerns | Low |
| DG:R+TMP/SMX | No concerns | Low risk | No concerns | Major concerns | No concerns | No concerns | Low |
| DG:R+Tetracyclines | Some concerns | Low risk | No concerns | Some concerns | Some concerns | No concerns | Very low |
| DG:S+TMP/SMX | Some concerns | Low risk | No concerns | Some concerns | Some concerns | No concerns | Very low |
| DG:S+Tetracyclines | Some concerns | Low risk | No concerns | Major concerns | No concerns | No concerns | Very low |
| DG:Single | Some concerns | Low risk | No concerns | Major concerns | No concerns | No concerns | Very low |
| DG:Triple | No concerns | Low risk | No concerns | Major concerns | No concerns | No concerns | Low |
| DR:R+TMP/SMX | No concerns | Low risk | No concerns | Major concerns | No concerns | No concerns | Low |
| DR:S+TMP/SMX | Some concerns | Low risk | No concerns | Some concerns | Some concerns | No concerns | Very low |
| DS:R+TMP/SMX | No concerns | Low risk | No concerns | Some concerns | Some concerns | No concerns | Low |
| DS:R+Tetracyclines | Some concerns | Low risk | No concerns | Some concerns | Some concerns | No concerns | Very low |
| DS:S+TMP/SMX | Some concerns | Low risk | No concerns | No concerns | Some concerns | No concerns | Low |
| DS:Triple | No concerns | Low risk | No concerns | Major concerns | No concerns | No concerns | Low |
| R+Quinolones:R+TMP/SMX | No concerns | Low risk | No concerns | No concerns | Some concerns | No concerns | Moderate |
| R+Quinolones:R+Tetracyclines | Some concerns | Low risk | No concerns | Major concerns | No concerns | No concerns | Very low |
| R+Quinolones:S+TMP/SMX | Some concerns | Low risk | No concerns | No concerns | No concerns | No concerns | Moderate |
| R+Quinolones:S+Tetracyclines | Some concerns | Low risk | No concerns | No concerns | No concerns | No concerns | Moderate |
| R+Quinolones:Single | Some concerns | Low risk | No concerns | Major concerns | No concerns | No concerns | Very low |
| R+Quinolones:Triple | Some concerns | Low risk | No concerns | Some concerns | Some concerns | No concerns | Very low |
| R+Tetracyclines:R+TMP/SMX | No concerns | Low risk | No concerns | No concerns | No concerns | No concerns | High |
| R+TMP/SMX:S+TMP/SMX | No concerns | Low risk | No concerns | Major concerns | No concerns | No concerns | Low |
| R+TMP/SMX:S+Tetracyclines | Some concerns | Low risk | No concerns | Major concerns | No concerns | No concerns | Very low |
| R+TMP/SMX:Single | Some concerns | Low risk | No concerns | Major concerns | No concerns | No concerns | Very low |
| R+TMP/SMX:Triple | No concerns | Low risk | No concerns | Major concerns | No concerns | No concerns | Low |
| R+Tetracyclines:S+TMP/SMX | Some concerns | Low risk | No concerns | No concerns | No concerns | No concerns | Moderate |
| R+Tetracyclines:S+Tetracyclines | Some concerns | Low risk | No concerns | No concerns | No concerns | No concerns | Moderate |
| S+Tetracyclines:S+TMP/SMX | Some concerns | Low risk | No concerns | Major concerns | No concerns | No concerns | Very low |
| Single:S+TMP/SMX | Some concerns | Low risk | No concerns | Some concerns | Some concerns | No concerns | Very low |
| S+TMP/SMX:Triple | Some concerns | Low risk | No concerns | Some concerns | Some concerns | No concerns | Very low |
| Single:S+Tetracyclines | Major concerns | Low risk | No concerns | Major concerns | No concerns | No concerns | Very low |
| S+Tetracyclines:Triple | Some concerns | Low risk | No concerns | Major concerns | No concerns | No concerns | Very low |
| Single:Triple | Some concerns | Low risk | No concerns | Major concerns | No concerns | No concerns | Very low |
