# Supplementary material for: Updated therapeutic options for human brucellosis: A systematic review and network meta-analysis of randomized controlled trials
Source: PLoS Negl Trop Dis. 2024 Aug 22;18(8):e0012405. doi: 10.1371/journal.pntd.0012405 (PMC11340890; doi:10.1371/journal.pntd.0012405)
Supplement: S11 Table — (DOCX) [file pntd.0012405.s011.docx]

**S11 Table**. League table of secondary outcomes in network meta-analysis

Relapse (pale yellow boxes) and therapeutic failure (pale blue boxes) are reported as relative risk with 95% CIs. For relapse and therapeutic failure, relative risk below 1 favour the column-defining treatment.

| Triple |  |  |  |  |  |  |  |  |  |  |  |
| --- | --- | --- | --- | --- | --- | --- | --- | --- | --- | --- | --- |
|  |  |  |  |  |  |  |  |  |  |  |  |
| 0.14 (0.02,1.08) | R+Tetracyclines |  |  |  |  |  |  |  |  |  |  |
| **0.40 (0.17,0.96)** |  |  |  |  |  |  |  |  |  |  |  |
| 0.43 (0.11,1.72) | 3.15 (0.26,37.99) | R+TMP/SMX |  |  |  |  |  |  |  |  |  |
| 0.35 (0.05,2.35) | 0.88 (0.12,6.58) |  |  |  |  |  |  |  |  |  |  |
| **0.45 (0.21,0.96)** | 3.27 (0.36,29.64) | 1.04 (0.25,4.25) | R+Quinolones |  |  |  |  |  |  |  |  |
| 0.73 (0.25,2.13) | 1.83 (0.52,6.49) | 2.08 (0.29,15.04) |  |  |  |  |  |  |  |  |  |
| 0.78 (0.36,1.66) | 5.70 (0.63,51.53) | 1.81 (0.44,7.36) | 1.75 (0.85,3.60) | DS |  |  |  |  |  |  |  |
| 0.80 (0.28,2.27) | 1.98 (0.57,6.91) | 2.26 (0.31,16.21) | 1.08 (0.39,3.04) |  |  |  |  |  |  |  |  |
| 1.22 (0.31,4.73) | 8.93 (0.75,105.83) | 2.83 (0.47,17.13) | 2.73 (0.72,10.43) | 1.57 (0.51,4.83) | DR |  |  |  |  |  |  |
| 1.59 (0.32,7.92) | 3.97 (0.70,22.64) | 4.52 (0.45,45.75) | 2.17 (0.44,10.66) | 2.00 (0.59,6.74) |  |  |  |  |  |  |  |
| 0.50 (0.15,1.63) | 3.68 (0.34,39.67) | 1.17 (0.56,2.43) | 1.13 (0.34,3.75) | 0.64 (0.19,2.13) | 0.41 (0.08,2.13) | DG |  |  |  |  |  |
| 0.81 (0.15,4.51) | 2.02 (0.32,12.78) | 2.30 (1.02,5.16) | 1.10 (0.18,6.71) | 1.02 (0.17,6.14) | 0.51 (0.06,4.46) |  |  |  |  |  |  |
| **0.16 (0.05,0.49)** | 1.21 (0.12,12.54) | 0.38 (0.08,1.92) | **0.37 (0.14,0.99)** | **0.21 (0.07,0.62)** | **0.14 (0.03,0.65)** | 0.33 (0.08,1.38) | D+TMP/SMX |  |  |  |  |
| **0.36 (0.16,0.82)** | 0.90 (0.35,2.29) | 1.02 (0.15,6.90) | 0.49 (0.19,1.27) | 0.45 (0.16,1.25) | 0.23 (0.05,1.10) | 0.45 (0.08,2.51) |  |  |  |  |  |
| **0.37 (0.22,0.62)** | 2.72 (0.32,22.89) | 0.86 (0.24,3.13) | 0.83 (0.47,1.48) | **0.48 (0.27,0.83)** | 0.30 (0.09,1.07) | 0.74 (0.26,2.13) | 2.25 (0.85,5.94) | D+Quinolones |  |  |  |
| **0.45 (0.25,0.81)** | 1.11 (0.45,2.73) | 1.27 (0.21,7.63) | 0.61 (0.26,1.41) | 0.56 (0.25,1.28) | 0.28 (0.06,1.22) | 0.55 (0.11,2.74) | 1.24 (0.65,2.36) |  |  |  |  |
| 0.15 (0.00,8.63) | 1.10 (0.01,104.06) | 0.35 (0.01,23.79) | 0.34 (0.01,18.85) | 0.19 (0.00,11.06) | 0.12 (0.00,8.24) | 0.30 (0.00,19.12) | 0.91 (0.02,45.06) | 0.40 (0.01,22.53) | S+TMP/SMX |  |  |
| 0.33 (0.01,18.08) | 0.82 (0.01,46.14) | 0.93 (0.01,73.07) | 0.45 (0.01,25.32) | 0.41 (0.01,23.72) | 0.21 (0.00,14.18) | 0.41 (0.01,29.45) | 0.91 (0.02,45.98) | 0.74 (0.01,39.16) |  |  |  |
| 0.08 (0.02,0.28) | 0.60 (0.05,6.70) | 0.19 (0.03,1.06) | 0.18 (0.05,0.64) | 0.11 (0.03,0.36) | 0.07 (0.01,0.36) | 0.16 (0.03,0.77) | 0.50 (0.12,2.08) | 0.22 (0.07,0.68) | 0.55 (0.01,34.93) | S+Tetracyclines |  |
| 0.13 (0.02,0.70) | 0.32 (0.05,2.00) | 0.36 (0.03,3.87) | 0.17 (0.03,0.93) | 0.16 (0.04,0.64) | 0.08 (0.01,0.51) | 0.16 (0.02,1.46) | 0.35 (0.07,1.88) | 0.28 (0.06,1.36) | 0.38 (0.01,27.44) |  |  |
| 0.06 (0.01,0.31) | 0.45 (0.03,6.24) | 0.14 (0.02,1.06) | 0.14 (0.03,0.68) | 0.08 (0.02,0.38) | 0.05 (0.01,0.35) | 0.12 (0.02,0.79) | 0.37 (0.08,1.76) | 0.17 (0.04,0.77) | 0.41 (0.01,27.26) | 0.75 (0.11,4.91) | Single |
| 0.11 (0.05,0.23) | 0.26 (0.12,0.56) | 0.30 (0.04,2.05) | 0.14 (0.05,0.40) | 0.13 (0.05,0.37) | 0.07 (0.01,0.32) | 0.13 (0.02,0.74) | 0.29 (0.15,0.57) | 0.24 (0.12,0.47) | 0.32 (0.01,17.23) | 0.84 (0.16,4.50) |  |
