# Supplementary material for: Updated therapeutic options for human brucellosis: A systematic review and network meta-analysis of randomized controlled trials
Source: PLoS Negl Trop Dis. 2024 Aug 22;18(8):e0012405. doi: 10.1371/journal.pntd.0012405 (PMC11340890; doi:10.1371/journal.pntd.0012405)
Supplement: S12 Table — (DOCX) [file pntd.0012405.s012.docx]

**S12 Table**. Heterogeneity estimates

**1. Overall failure**

Number of studies: k = 42

Number of pairwise comparisons: m = 50

Number of treatments: n = 11

Number of designs: d = 17

Random effects model

Quantifying heterogeneity / inconsistency:

tau^2 = 0; tau = 0; I^2 = 0% [0.0%; 37.4%]

Tests of heterogeneity (within designs) and inconsistency (between designs):

Q d.f. p-value

Total 34.68 36 0.5311

Within designs 18.19 26 0.8687

Between designs 16.49 10 0.0864

**2. Side effects**

Number of studies: k = 34

Number of pairwise comparisons: m = 42

Number of treatments: n = 12

Number of designs: d = 16

Random effects model

Quantifying heterogeneity / inconsistency:

tau^2 = 0.0929; tau = 0.3048; I^2 = 25.7% [0.0%; 53.5%]

Tests of heterogeneity (within designs) and inconsistency (between designs):

Q d.f. p-value

Total 36.36 27 0.1076

Within designs 32.49 19 0.0275

Between designs 03.87 8 0.8686

**3. Relapse**

Number of studies: k = 29

Number of pairwise comparisons: m = 37

Number of treatments: n = 11

Number of designs: d = 14

Random effects model

Quantifying heterogeneity / inconsistency:

tau^2 = 0; tau = 0; I^2 = 0% [0.0%; 44.6%]

Tests of heterogeneity (within designs) and inconsistency (between designs):

Q d.f. p-value

Total 9.57 23 0.9936

Within designs 6.81 16 0.9767

Between designs 2.76 7 0.9064

**4. Therapeutic failure**

Number of studies: k = 33

Number of pairwise comparisons: m = 41

Number of treatments: n = 11

Number of designs: d = 17

Random effects model

Quantifying heterogeneity / inconsistency:

tau^2 = 0.0475; tau = 0.2179; I^2 = 6.1% [0.0%; 37.4%]

Tests of heterogeneity (within designs) and inconsistency (between designs):

Q d.f. p-value

Total 28.74 27 0.3736

Within designs 13.38 17 0.7104

Between designs 15.36 10 0.1194
