# Supplementary material for: Updated therapeutic options for human brucellosis: A systematic review and network meta-analysis of randomized controlled trials
Source: PLoS Negl Trop Dis. 2024 Aug 22;18(8):e0012405. doi: 10.1371/journal.pntd.0012405 (PMC11340890; doi:10.1371/journal.pntd.0012405)
Supplement: S13 Table — (DOCX) [file pntd.0012405.s013.docx]

**S13 Table**. Inconsistency estimates

**1. Overall failure**

Global inconsistency

chi2( 11) = 17.04

Prob > chi2 = 0.1066

Local inconsistency

-Loop-specific heterogeneity estimates (number of loops: 13, number of consistency loop: 11, proportion of consistency loop: 84.6%)

| Loop | IF | seIF | z_value | p_value | CI_95 | Loop_Heterog_tau2 |
| --- | --- | --- | --- | --- | --- | --- |
| D-H-L | 2.951 | 1.210 | 2.440 | 0.015 | (0.58,5.32) | 0.006 |
| D-H-K | 2.719 | 1.262 | 2.155 | 0.031 | (0.25,5.19) | 0.000 |
| H-K-L | 1.955 | 1.022 | 1.913 | 0.056 | (0.00,3.96) | 0.000 |
| D-K-L | 1.716 | 1.020 | 1.683 | 0.092 | (0.00,3.72) | 0.000 |
| A-D-J | 1.468 | 2.194 | 0.669 | 0.503 | (0.00,5.77) | 0.154 |
| A-E-F-J | 1.108 | 2.200 | 0.504 | 0.614 | (0.00,5.42) | 0.000 |
| A-E-J-K | 1.001 | 2.561 | 0.391 | 0.696 | (0.00,6.02) | 0.000 |
| D-E-J | 0.530 | 0.982 | 0.539 | 0.590 | (0.00,2.46) | 0.000 |
| A-D-K | 0.344 | 0.824 | 0.417 | 0.677 | (0.00,1.96) | 0.000 |
| D-E-K | 0.328 | 1.553 | 0.212 | 0.832 | (0.00,3.37) | 0.000 |
| D-E-F | 0.128 | 0.553 | 0.231 | 0.817 | (0.00,1.21) | 0.000 |
| A-E-F-K | 0.107 | 1.557 | 0.069 | 0.945 | (0.00,3.16) | 0.000 |
| A-D-F | 0.033 | 0.621 | 0.053 | 0.958 | (0.00,1.25) | 0.000 |

**Note: Heterogeneity of loop H-K-L cannot be estimated due to insufficient observations - set equal to 0

**Note: Heterogeneity of loop A-E-F-J cannot be estimated due to insufficient observations - set equal to 0

-Node-splitting approach (number of comparisons: 20, number of consistency comparison: 18,

proportion of consistency comparison: 90%)

| Side | Direct |  | Indirect |  | Difference |  |  |
| --- | --- | --- | --- | --- | --- | --- | --- |
|  | Coef. | Std. Err. | Coef. | Std. Err. | Coef. | Std. Err. | P>\|z\| |
| D E | -.7420815 | .2447288 | -.4314678 | .8172123 | -.3106138 | .8349108 | 0.710 |
| D F * | -.2148069 | .281171 | -1.039115 | .7342886 | .8243081 | .7910204 | 0.297 |
| D H | -2.079442 | 1.028753 | .6709943 | .3793799 | -2.750436 | 1.096477 | 0.012 |
| D J | 2.174284 | .6210511 | .8328846 | 1.261662 | 1.341399 | 1.515982 | 0.376 |
| D K | 1.854666 | .6601378 | 1.56492 | .3498171 | .2897458 | .7471475 | 0.698 |
| D L | -.9966062 | .2196188 | -.5938426 | .6295371 | -.4027636 | .665045 | 0.545 |
| A D | -.6933601 | .3463648 | .0323067 | .4789136 | -.7256667 | .6138066 | 0.237 |
| A F | -1.100268 | .4837739 | -.4476707 | .4582197 | -.6525968 | .6647725 | 0.326 |
| A I * | .0935261 | 1.996511 | -.9380129 | 6651.991 | 1.031539 | 6651.991 | 1.000 |
| A J | -1.11e-10 | 1.994764 | 1.542659 | .5880941 | -1.542659 | 2.079649 | 0.458 |
| A K | 1.535939 | .3850449 | .6141083 | .5022955 | .9218309 | .6341603 | 0.146 |
| B D * | .4340639 | .448858 | .297427 | 622.7351 | .1366369 | 622.7353 | 1.000 |
| B G * | .5198755 | .2858627 | .8273125 | 1314.974 | -.307437 | 1314.974 | 1.000 |
| C E * | .5690469 | .4202243 | -1.270675 | 1676.294 | 1.839722 | 1676.294 | 0.999 |
| E F | .2698595 | .4074329 | .614644 | .5045245 | -.3447845 | .6507027 | 0.596 |
| E J | 2.539758 | .7296336 | 2.660048 | 1.210894 | -.1202907 | 1.601294 | 0.940 |
| E K | 2.908714 | 1.399375 | 2.308034 | .3947222 | .6006794 | 1.453979 | 0.680 |
| H K | 1.247173 | .3717325 | 1.618816 | .6638738 | -.3716437 | .7463291 | 0.619 |
| H L | -1.839012 | .6243525 | -.9081712 | .4888041 | -.9308412 | .7929347 | 0.240 |
| K L | -1.098612 | .7752936 | -2.894959 | .3736544 | 1.796347 | .860638 | 0.037 |

Legend: A: D+Quinolones, B: D+TMP/SMX, C: DG, D: DR, E: DS, F: R+Quinolones, G: R+TMP/SMX, H: R+Tetracyclines, I: S+TMP/SMX, J: S+Tetracyclines, K: Single, L: Triple

**2. Side effects**

Global inconsistency

chi2( 10) = 2.78

Prob > chi2 = 0.9862

Local inconsistency

-Loop-specific heterogeneity estimates (number of loops: 11, number of consistency loop: 11, proportion of consistency loop: 100%)

| Loop | IF | seIF | z_value | p_value | CI_95 | Loop_Heterog_tau2 |
| --- | --- | --- | --- | --- | --- | --- |
| D-E-J | 1.576 | 2.066 | 0.763 | 0.446 | (0.00,5.62) | 0.121 |
| A-D-J | 1.155 | 1.789 | 0.645 | 0.519 | (0.00,4.66) | 0.000 |
| A-E-F-J | 1.018 | 1.455 | 0.700 | 0.484 | (0.00,3.87) | 0.000 |
| A-E-J-K | 0.595 | 2.367 | 0.252 | 0.801 | (0.00,5.24) | 0.000 |
| A-D-F | 0.534 | 1.069 | 0.500 | 0.617 | (0.00,2.63) | 0.305 |
| D-E-F | 0.526 | 0.513 | 1.026 | 0.305 | (0.00,1.53) | 0.032 |
| D-H-K | 0.443 | 2.053 | 0.216 | 0.829 | (0.00,4.47) | 0.000 |
| A-E-F-K | 0.422 | 2.075 | 0.204 | 0.839 | (0.00,4.49) | 0.000 |
| D-H-L | 0.301 | 1.295 | 0.233 | 0.816 | (0.00,2.84) | 0.308 |
| D-E-K | 0.266 | 2.786 | 0.095 | 0.924 | (0.00,5.73) | 0.121 |
| A-D-K | 0.055 | 1.978 | 0.028 | 0.978 | (0.00,3.93) | 0.000 |

**Note: Heterogeneity of loop A-D-J cannot be estimated due to insufficient observations - set equal to 0

**Note: Heterogeneity of loop D-H-K cannot be estimated due to insufficient observations - set equal to 0

**Note: Heterogeneity of loop A-E-F-J cannot be estimated due to insufficient observations - set equal to 0

-Node-splitting approach (number of comparisons: 19, number of consistency comparison: 19,

proportion of consistency comparison: 100%)

| Side | Direct |  | Indirect |  | Difference |  |  |
| --- | --- | --- | --- | --- | --- | --- | --- |
|  | Coef. | Std. Err. | Coef. | Std. Err. | Coef. | Std. Err. | P>\|z\| |
| D E | -.299631 | .2515666 | -1.163707 | .870201 | .8640757 | .9012835 | 0.338 |
| D F * | -.6174885 | .2683741 | -.6691079 | .8320685 | .0516194 | .8625031 | 0.952 |
| D H | -1.011601 | .6235112 | -1.113843 | .6089304 | .1022424 | .8715288 | 0.907 |
| D J | 1.435299 | 1.562584 | .6734426 | .7881615 | .7618561 | 1.831036 | 0.677 |
| D K | -.3364722 | 1.939428 | -.05498 | .4460996 | -.2814922 | 1.990072 | 0.888 |
| D L | -.0776448 | .2091466 | -.5112551 | 1.034265 | .4336102 | 1.055453 | 0.681 |
| A D | .1121773 | .4961922 | -.3158217 | .6037452 | .427999 | .7801249 | 0.583 |
| A F | -.8935481 | .6515106 | -.4975103 | .6054584 | -.3960377 | .9191357 | 0.667 |
| A I * | 1.106911 | .6361974 | -.3757442 | 1670.962 | 1.482655 | 1670.962 | 0.999 |
| A J | .8472978 | .732526 | .6345487 | 1.179154 | .2127491 | 1.388164 | 0.878 |
| A K | -.1798036 | .3533332 | .1606764 | .8194687 | -.34048 | .8911978 | 0.702 |
| B D * | -.0509379 | .3913875 | .8084301 | 1606.579 | -.859368 | 1606.579 | 1.000 |
| B G * | 1.252763 | .8547463 | -1.347714 | 4007.541 | 2.600478 | 4007.541 | 0.999 |
| C E * | -.200967 | .2909526 | -.6644626 | 789.3936 | .4634956 | 789.3937 | 1.000 |
| E F | -.1098338 | .3790448 | -.5562541 | .5186336 | .4464203 | .6414461 | 0.486 |
| E J | .6612436 | 1.176863 | 1.48412 | .8357728 | -.8228765 | 1.444172 | 0.569 |
| E K | .2006707 | 1.975369 | .2984281 | .4916037 | -.0977574 | 2.035622 | 0.962 |
| H K | 1.189876 | .6090855 | .713112 | .7377418 | .4767645 | .9566985 | 0.618 |
| H L | .6458943 | .9105874 | 1.079504 | .5336801 | -.4336097 | 1.055454 | 0.681 |

Legend: A: D+Quinolones, B: D+TMP/SMX, C: DG, D: DR, E: DS, F: R+Quinolones, G: R+TMP/SMX, H: R+Tetracyclines, I: S+TMP/SMX, J: S+Tetracyclines, K: Single, L: Triple

**3. Relapse**

Global inconsistency

chi2( 10) = 2.92

Prob > chi2 = 0.9394

Local inconsistency

-Loop-specific heterogeneity estimates (number of loops: 9, number of consistency loop: 9, proportion of consistency loop: 100%)

| Loop | IF | seIF | z_value | p_value | CI_95 | Loop_Heterog_tau2 |
| --- | --- | --- | --- | --- | --- | --- |
| A-E-J-K | 3.340 | 3.115 | 1.072 | 0.284 | (0.00,9.45) | 0.000 |
| A-E-F-J | 1.871 | 2.596 | 0.721 | 0.471 | (0.00,6.96) | 0.000 |
| D-E-K | 1.570 | 2.095 | 0.749 | 0.454 | (0.00,5.68) | 0.000 |
| A-E-F-K | 1.468 | 2.083 | 0.705 | 0.481 | (0.00,5.55) | 0.000 |
| D-E-J | 1.006 | 1.605 | 0.627 | 0.531 | (0.00,4.15) | 0.000 |
| A-D-J | 0.427 | 2.191 | 0.195 | 0.845 | (0.00,4.72) | 0.000 |
| A-D-K | 0.374 | 1.867 | 0.200 | 0.841 | (0.00,4.03) | 0.000 |
| D-E-F | 0.340 | 0.688 | 0.493 | 0.622 | (0.00,1.69) | 0.000 |
| A-D-F | 0.247 | 1.001 | 0.247 | 0.805 | (0.00,2.21) | 0.000 |

**Note: Heterogeneity of loop A-D-J cannot be estimated due to insufficient observations - set equal to 0

**Note: Heterogeneity of loop A-D-K cannot be estimated due to insufficient observations - set equal to 0

**Note: Heterogeneity of loop A-E-F-J cannot be estimated due to insufficient observations - set equal to 0

**Note: Heterogeneity of loop A-E-F-K cannot be estimated due to insufficient observations - set equal to 0

**Note: Heterogeneity of loop A-E-J-K cannot be estimated due to insufficient observations - set equal to 0

-Node-splitting approach (number of comparisons: 17, number of consistency comparison: 17,

proportion of consistency comparison: 100%)

| Side | Direct |  | Indirect |  | Difference |  |  |
| --- | --- | --- | --- | --- | --- | --- | --- |
|  | Coef. | Std. Err. | Coef. | Std. Err. | Coef. | Std. Err. | P>\|z\| |
| D E | -.8006774 | .2885237 | .3512302 | 1.014758 | -1.151908 | 1.028491 | 0.263 |
| D F * | -.1442358 | .3017299 | -.6780121 | 1.019681 | .5337763 | 1.053274 | 0.612 |
| D J | 1.479178 | .6216266 | 1.694608 | 1.685919 | -.2154296 | 1.824317 | 0.906 |
| D K | 2.061423 | 1.356083 | 1.667316 | .9656631 | .3941075 | 1.664772 | 0.813 |
| D L * | -.9922027 | .2638181 | -1.389621 | 1534.765 | .3974181 | 1534.765 | 1.000 |
| A D | -1.004365 | .6610578 | -.4588288 | .9452391 | -.5455361 | 1.243476 | 0.661 |
| A F | -1.064357 | .6562368 | -.8781872 | .8685517 | -.1861695 | 1.126608 | 0.869 |
| A I * | .0935256 | 1.990612 | -2.034821 | 7264.08 | 2.128346 | 7264.081 | 1.000 |
| A J | -7.59e-13 | 1.988338 | .8032842 | .7836485 | -.8032842 | 2.137193 | 0.707 |
| A K | 1.417066 | 1.090159 | .5053597 | 1.155699 | .9117059 | 1.588737 | 0.566 |
| B D * | .3009186 | .5403062 | .0833883 | 971.7089 | .2175303 | 971.709 | 1.000 |
| B G * | .1541507 | .3748015 | .5943706 | 2044.669 | -.4402199 | 2044.669 | 1.000 |
| C E * | .4481171 | .5748136 | -1.480727 | 2162.539 | 1.928844 | 2162.539 | 0.999 |
| E F | .3493284 | .4905915 | .8436218 | .5780826 | -.4942935 | .7668774 | 0.519 |
| E J | 3.122315 | 1.279271 | 1.253149 | 1.414309 | 1.869165 | 2.385267 | 0.433 |
| E K | 1.299282 | 1.569919 | 2.987214 | .9425475 | -1.687931 | 1.831131 | 0.357 |
| H L * | -1.993163 | 1.054203 | -1.156436 | 2432.938 | -.8367267 | 2432.938 | 1.000 |

Legend: A: D+Quinolones, B: D+TMP/SMX, C: DG, D: DR, E: DS, F: R+Quinolones, G: R+TMP/SMX, H: R+Tetracyclines, I: S+TMP/SMX, J: S+Tetracyclines, K: Single, L: Triple

**4. Therapeutic failure**

Global inconsistency

chi2( 10) = 15.03

Prob > chi2 = 0.1309

Local inconsistency

-Loop-specific heterogeneity estimates (number of loops: 10, number of consistency loop: 9, proportion of consistency loop: 90%)

| Loop | IF | seIF | z_value | p_value | CI_95 | Loop_Heterog_tau2 |
| --- | --- | --- | --- | --- | --- | --- |
| D-H-L | 2.964 | 1.370 | 2.164 | 0.030 | (0.28,5.65) | 0.074 |
| D-E-J | 2.524 | 1.688 | 1.496 | 0.135 | (0.00,5.83) | 0.000 |
| D-H-K | 2.376 | 1.283 | 1.852 | 0.064 | (0.00,4.89) | 0.000 |
| H-K-L | 1.868 | 1.117 | 1.673 | 0.094 | (0.00,4.06) | 0.000 |
| D-K-L | 1.237 | 1.072 | 1.154 | 0.249 | (0.00,3.34) | 0.000 |
| D-E-K | 0.697 | 1.615 | 0.431 | 0.666 | (0.00,3.86) | 0.000 |
| A-D-K | 0.563 | 0.891 | 0.632 | 0.528 | (0.00,2.31) | 0.000 |
| D-E-F | 0.376 | 0.992 | 0.379 | 0.705 | (0.00,2.32) | 0.000 |
| A-D-F | 0.122 | 0.911 | 0.134 | 0.893 | (0.00,1.91) | 0.000 |
| A-E-F-K | 0.084 | 1.728 | 0.048 | 0.961 | (0.00,3.47) | 0.000 |

**Note: Heterogeneity of loop H-K-L cannot be estimated due to insufficient observations - set equal to 0

-Node-splitting approach (number of comparisons: 19, number of consistency comparison: 18,

proportion of consistency comparison: 94.7%)

| Side | Direct |  | Indirect |  | Difference |  |  |
| --- | --- | --- | --- | --- | --- | --- | --- |
|  | Coef. | Std. Err. | Coef. | Std. Err. | Coef. | Std. Err. | P>\|z\| |
| D E * | -.5592422 | .4505717 | -.6079527 | 1.124275 | .0487105 | 1.189076 | 0.967 |
| D F * | -.3715438 | .4827356 | -1.054464 | 1.111794 | .6829198 | 1.211113 | 0.573 |
| D H | -2.079442 | 1.028753 | .5356758 | .4276121 | -2.615117 | 1.114085 | 0.019 |
| D J * | 3.224574 | 1.463653 | -1.864439 | 2.100152 | 5.089012 | 3.198353 | 0.112 |
| DK | 1.502444 | .7118238 | 1.417835 | .4048923 | .084609 | .8195713 | 0.918 |
| D L | -.8696949 | .3503443 | -.6067366 | .7147964 | -.2629583 | .7862002 | 0.738 |
| A D | -.5764915 | .4408409 | .4227579 | .5720337 | -.9992494 | .7406752 | 0.177 |
| A F | -1.109281 | .7217825 | -.3238988 | .6874478 | -.7853825 | .9813754 | 0.424 |
| A I * | .093526 | 2.000879 | -.3177505 | 6322.325 | .4112765 | 6322.325 | 1.000 |
| A K | 1.558688 | .4227998 | .5665166 | .5991783 | .9921711 | .7383747 | 0.179 |
| B D * | .5956487 | .8173779 | .5020787 | 861.8103 | .09357 | 861.8106 | 1.000 |
| B G * | .8329091 | .4125252 | 1.02725 | 1608.774 | -.1943404 | 1608.774 | 1.000 |
| C E * | .6931271 | .6199881 | -1.263364 | 2762.241 | 1.956492 | 2762.241 | 0.999 |
| E F | .0995203 | .686873 | .062011 | .8484948 | .0375092 | 1.09387 | 0.973 |
| E J * | 1.445237 | .7344324 | 6.534243 | 3.036339 | -5.089006 | 3.198359 | 0.112 |
| E K | 2.765615 | 1.418143 | 1.89105 | .5682642 | .8745642 | 1.527761 | 0.567 |
| H K | 1.2769 | .4223506 | 1.683318 | .7782592 | -.4064174 | .8569306 | 0.635 |
| H L | -1.752001 | .7911548 | -.4957806 | .5746426 | -1.25622 | .9778241 | 0.199 |
| K L | -1.098612 | .7914915 | -2.610476 | .4776565 | 1.511864 | .9244537 | 0.102 |

Legend: A: D+Quinolones, B: D+TMP/SMX, C: DG, D: DR, E: DS, F: R+Quinolones, G: R+TMP/SMX, H: R+Tetracyclines, I: S+TMP/SMX, J: S+Tetracyclines, K: Single, L: Triple
