# Supplementary material for: Updated therapeutic options for human brucellosis: A systematic review and network meta-analysis of randomized controlled trials
Source: PLoS Negl Trop Dis. 2024 Aug 22;18(8):e0012405. doi: 10.1371/journal.pntd.0012405 (PMC11340890; doi:10.1371/journal.pntd.0012405)
Supplement: S14 Table — (DOCX) [file pntd.0012405.s014.docx]

**S14 Table**. Subgroup analyses for primary outcomes

**1. Subgroup network meta-analyses for overall failure**

| **Subgroups** | **Number of studies** | **Subgroup network meta-analyses of each therapy for overall failure compared with DR with relative risk (95% CI)** | | | | | | | | | | |
| --- | --- | --- | --- | --- | --- | --- | --- | --- | --- | --- | --- | --- |
|  |  | **DG** | **DS** | **Triple** | **D+TMP/SMX** | **R+Quinolones** | **R+TMP/SMX** | **D+Quinolones** | **R+Tetracyclines** | **S+Tetracyclines** | **Single** | **S+TMP/SMX** |
| **All trials** | 44 | **0.27 (0.11, 0.68)** | **0.48 (0.31, 0.76)** | **0.39 (0.28, 0.56)** | 0.64 (0.27, 1.50) | 0.71 (0.45, 1.10) | 1.08 (0.41, 2.85) | 1.52 (0.91, 2.52) | 1.39 (0.69, 2.80) | **7.03 (2.62, 18.87)** | **5.05 (2.81, 9.07)** | …… |
| **Geographical region** |  |  |  |  |  |  |  |  |  |  |  |  |
| other regions | 24 | **0.28 (0.11, 0.70)** | **0.49 (0.31, 0.78)** | 0.60 (0.35, 1.04) | 0.64 (0.27, 1.50) | 0.74 (0.47, 1.16) | 1.08 (0.41, 2.85) | **1.86 (1.04, 3.32)** | 3.09 (0.40, 23.62) | **7.09 (2.64, 19.03)** | **8.33 (1.24, 55.87)** | …… |
| China | 20 | …… | …… | **0.26 (0.16, 0.41)** | …… | …… | …… | 0.64 (0.20, 2.11) | 0.64 (0.25, 1.64) | …… | **2.96 (1.17, 7.54)** | …… |
| **Follow-up time** | 34 |  |  |  |  |  |  |  |  |  |  |  |
| less than or equal to 6 months | 28 | …… | **0.41 (0.18, 0.96)** | **0.34 (0.22, 0.53)** | 0.67 (0.25, 1.74) | 0.73 (0.36, 1.50) | …… | 0.51 (0.15, 1.76) | 0.82 (0.33, 2.08) | …… | **2.76 (1.08, 7.03)** | …… |
| more than 6 months | 6 | 0.48 (0.14, 1.65) | 0.85 (0.33, 2.18) | …… | …… | 1.13 (0.51, 2.51) | …… | …… | …… | …… | …… | …… |

**2. Subgroup network meta-analyses for side effects**

| **Subgroups** | **Number of studies** | **Subgroup network meta-analyses of each therapy for side effects compared with DR with relative risk (95% CI)** | | | | | | | | | | |
| --- | --- | --- | --- | --- | --- | --- | --- | --- | --- | --- | --- | --- |
|  |  | **DG** | **DS** | **Triple** | **D+TMP/SMX** | **R+Quinolones** | **R+TMP/SMX** | **D+Quinolones** | **R+Tetracyclines** | **S+Tetracyclines** | **Single** | **S+TMP/SMX** |
| **All trials** | 37 | 0.84 (0.41, 1.72) | 0.69 (0.44, 1.08) | 0.91 (0.61, 1.34) | 1.05 (0.50, 2.19) | **0.53 (0.33, 0.87)** | 3.66 (0.59, 22.64) | 1.07 (0.51, 2.27) | **0.35 (0.15, 0.81)** | 2.35 (0.64, 8.60) | 0.95 (0.39, 2.29) | 3.24 (0.77, 13.71) |
| **Geographical region** | 37 |  |  |  |  |  |  |  |  |  |  |  |
| other regions | 20 | …… | …… | …… | …… | …… | …… | …… | …… | …… | …… | …… |
| China | 17 | …… | …… | 0.78 (0.48, 1.27) | …… | …… | …… | 1.01 (0.15, 6.65) | 0.38 (0.13, 1.10) | 2.36 (0.21, 26.31) | 0.85 (0.15, 4.85) | 3.06 (0.30, 30.79) |
| **Follow-up time** | 25 |  |  |  |  |  |  |  |  |  |  |  |
| less than or equal to 6 months | 17 | …… | 1.06 (0.30, 3.79) | 0.93 (0.54, 1.59) | 1.17 (0.37, 3.65) | 0.79 (0.32, 1.94) | …… | 0.50 (0.04, 6.82) | 0.40 (0.12, 1.38) | …… | 8.70 (0.33, 229.75) | …… |
| more than 6 months | 8 | 0.65 (0.38, 1.11) | **0.53 (0.36, 0.79)** | …… | …… | **0.35 (0.19, 0.63)** | …… | 0.71 (0.06, 8.27) | …… | 1.65 (0.20, 13.44) | …… | …… |
