# Supplementary material for: Updated therapeutic options for human brucellosis: A systematic review and network meta-analysis of randomized controlled trials
Source: PLoS Negl Trop Dis. 2024 Aug 22;18(8):e0012405. doi: 10.1371/journal.pntd.0012405 (PMC11340890; doi:10.1371/journal.pntd.0012405)

**S1 Fig**. Pairwise meta-analyses results

**1. Overall failure**

1.1 D+Quinolones vs DR


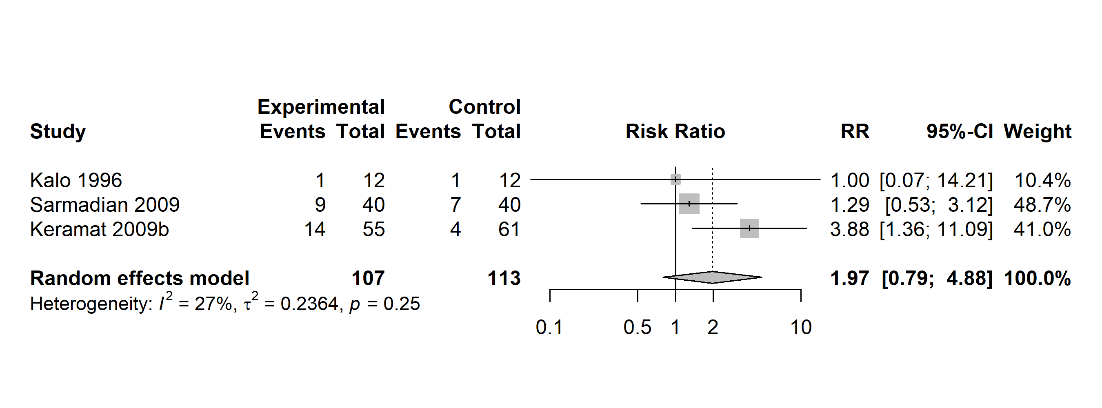


1.2 D+TMP/SMX vs DR


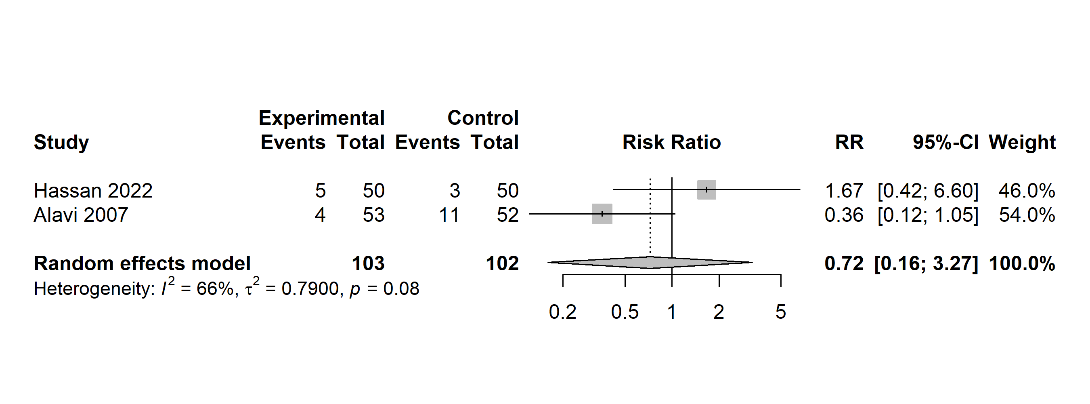


1.3 DS vs DR


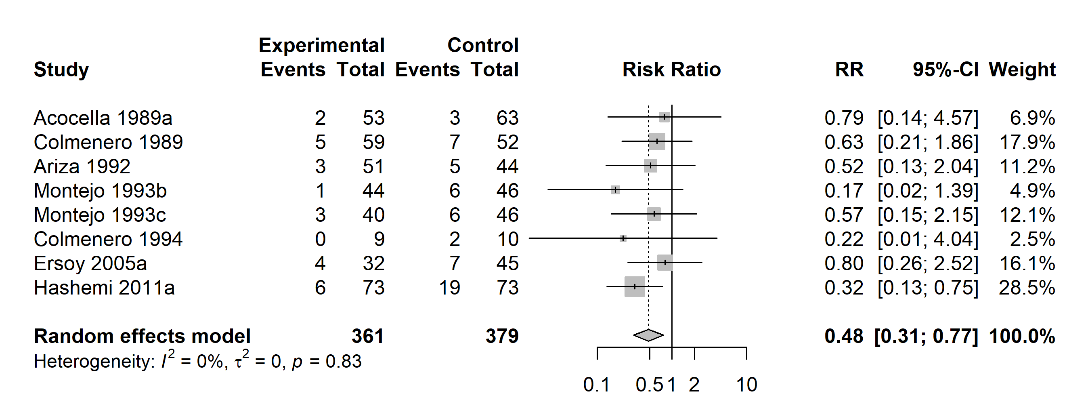


1.4 R+Quinolones vs DR


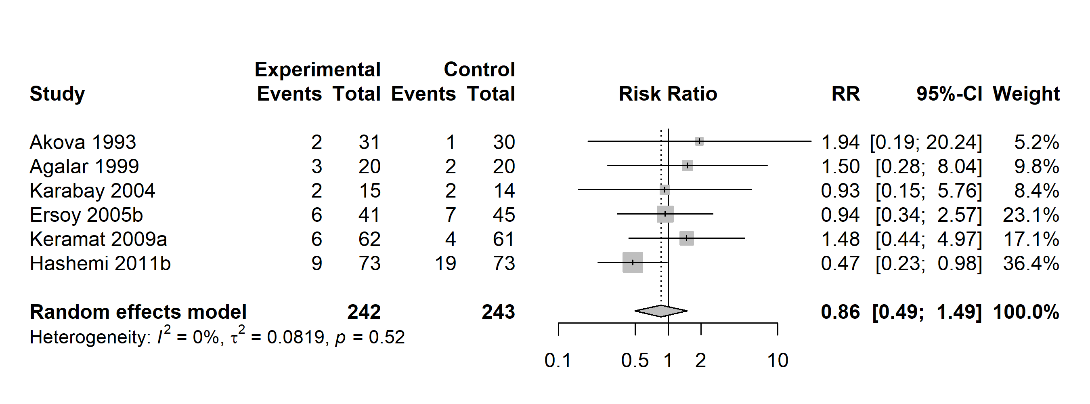


1.5 R+Tetracyclines vs DR


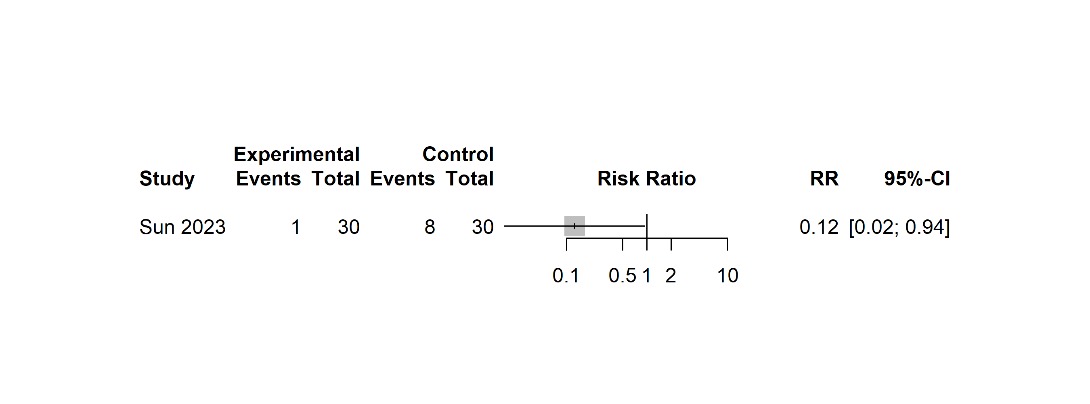


1.6 S+Tetracyclines vs DR


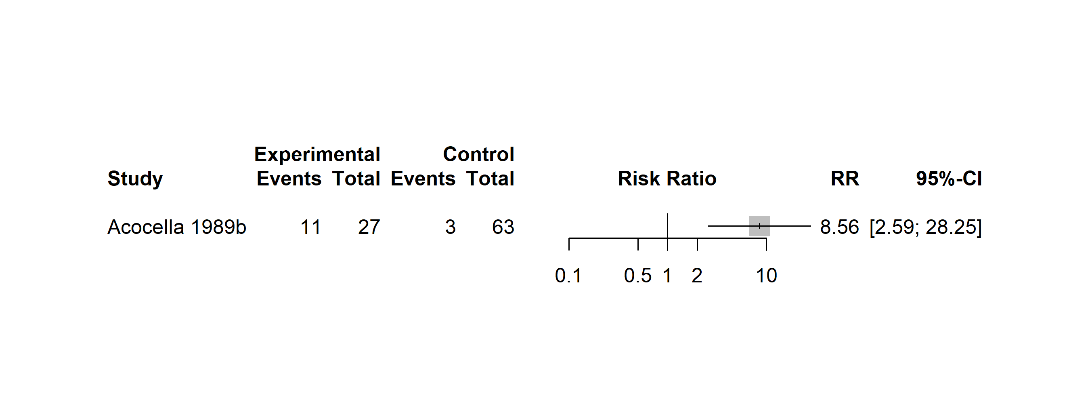


1.7 Single vs DR


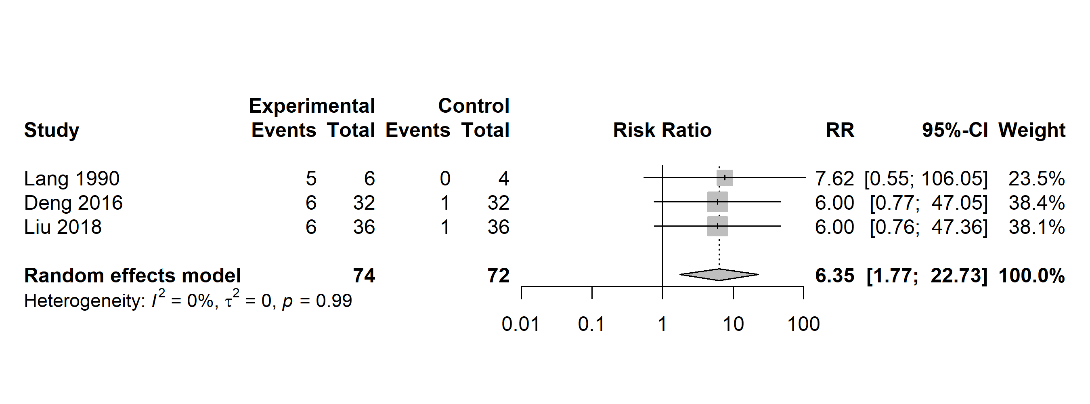


1.8 Triple vs DR


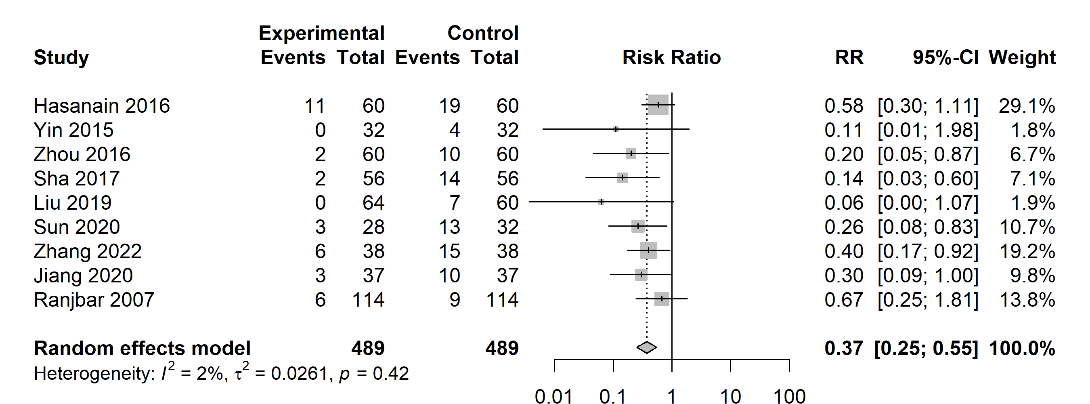


**2. Side effects**

2.1 D+Quinolones vs DR


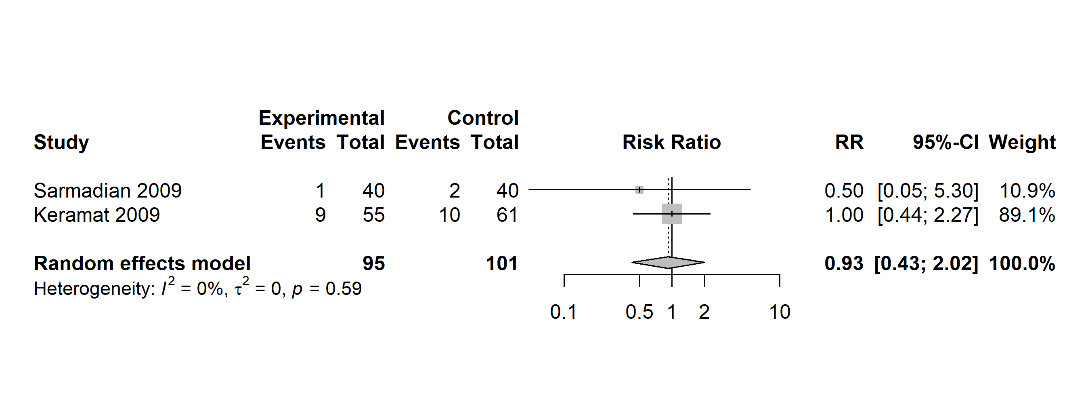


2.2 D+TMP/SMX vs DR


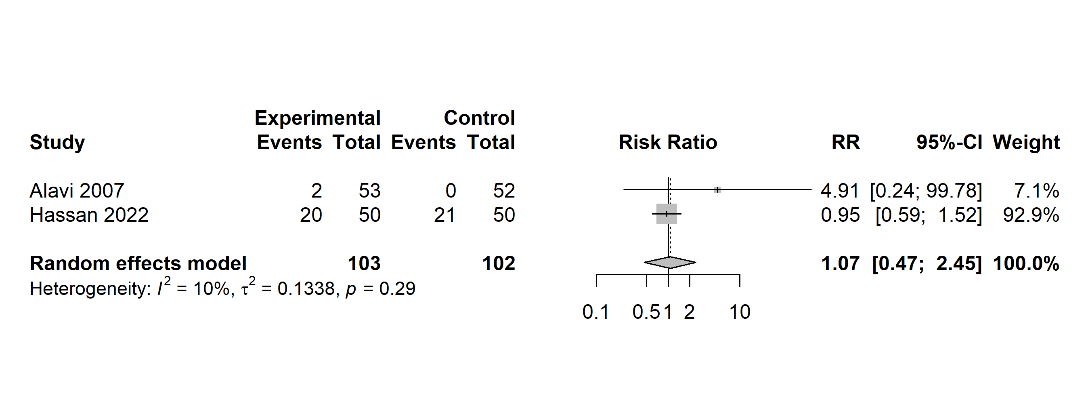


2.3 DS vs DR


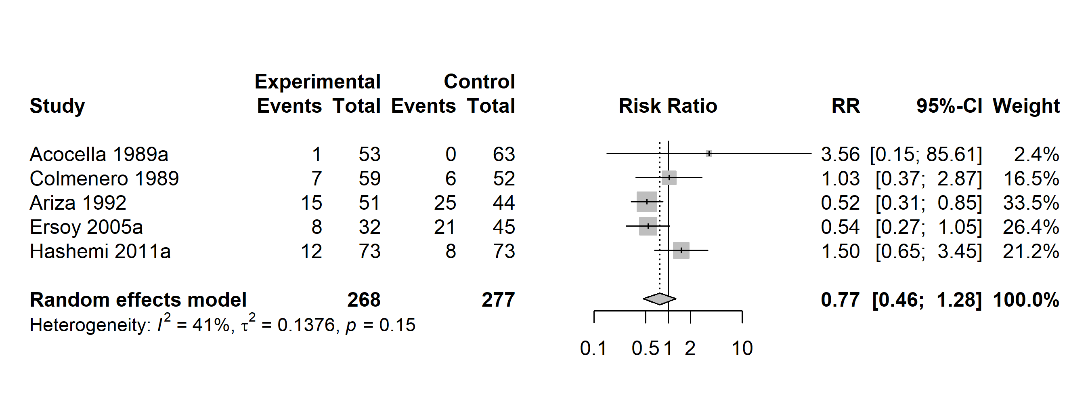


2.4 R+Quinolones vs DR


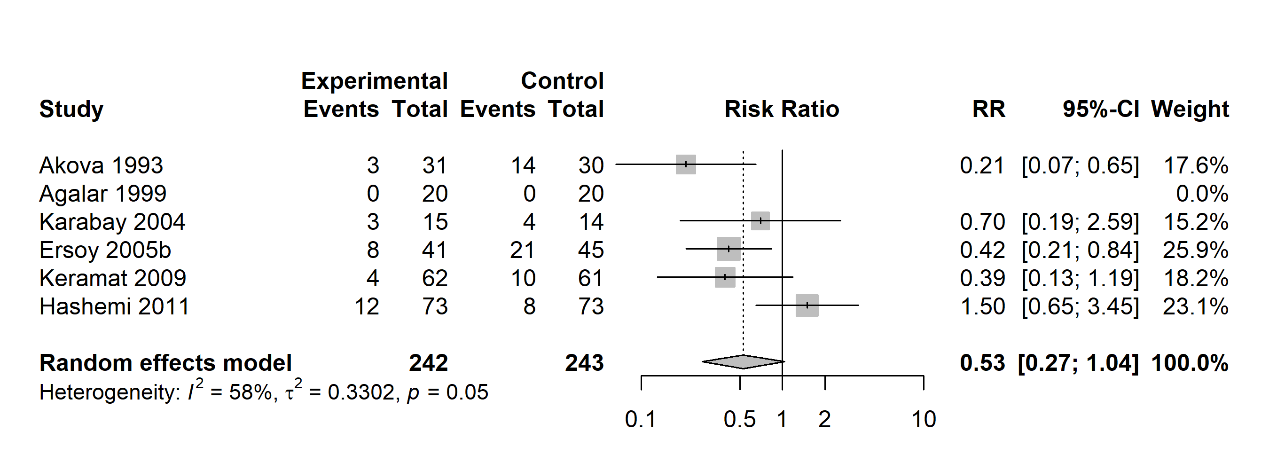


2.5 R+Tetracyclines vs DR


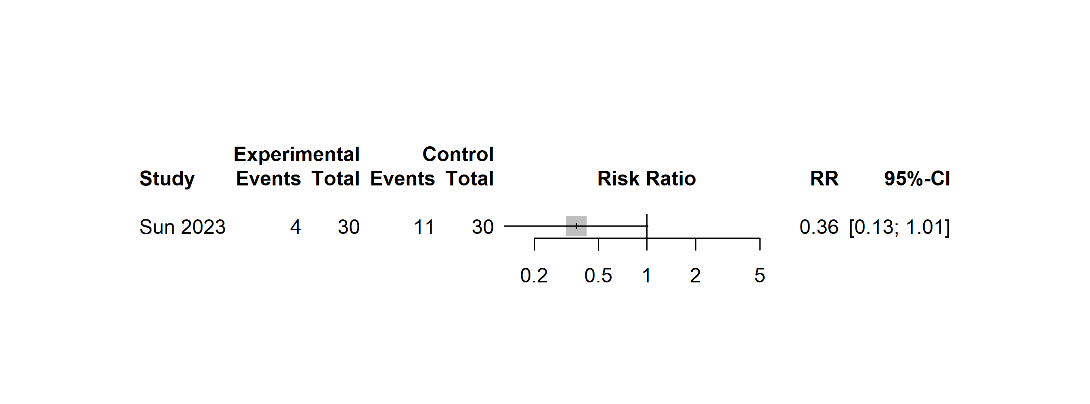


2.6 S+Tetracyclines vs DR


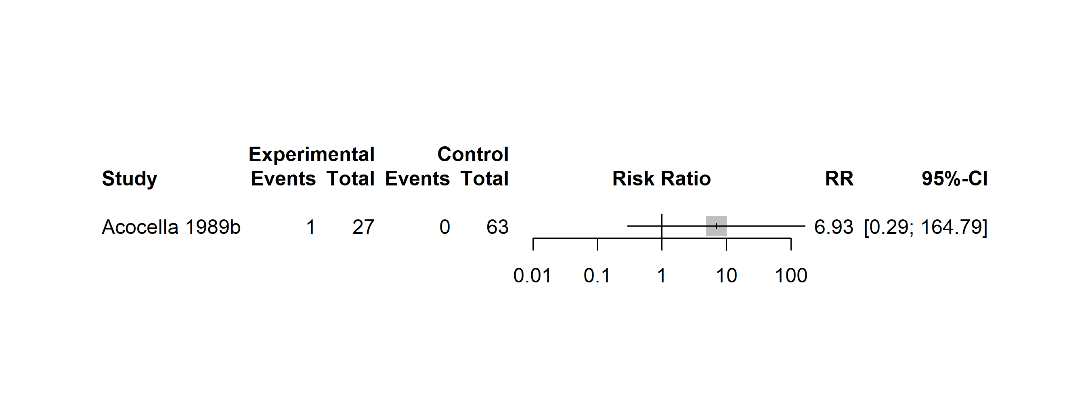


2.7 Single vs DR


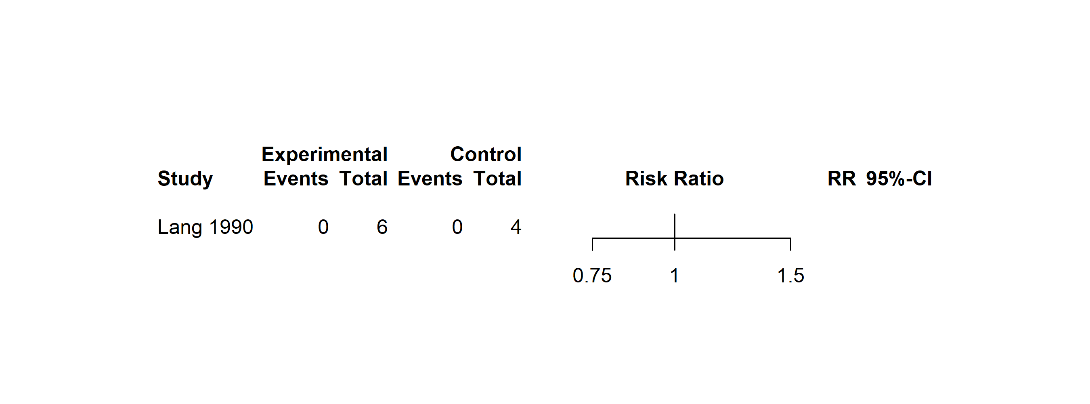


2.8 Triple vs DR


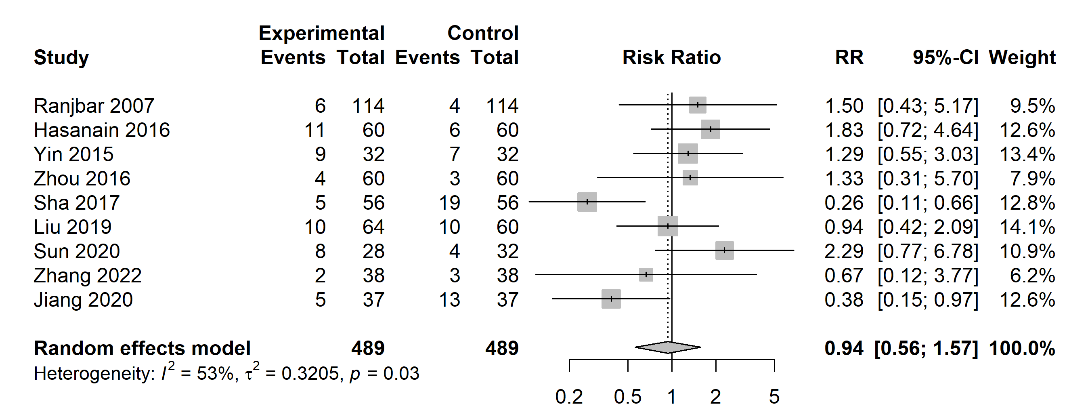


**3. Relapse**

3.1 D+Quinolones vs DR


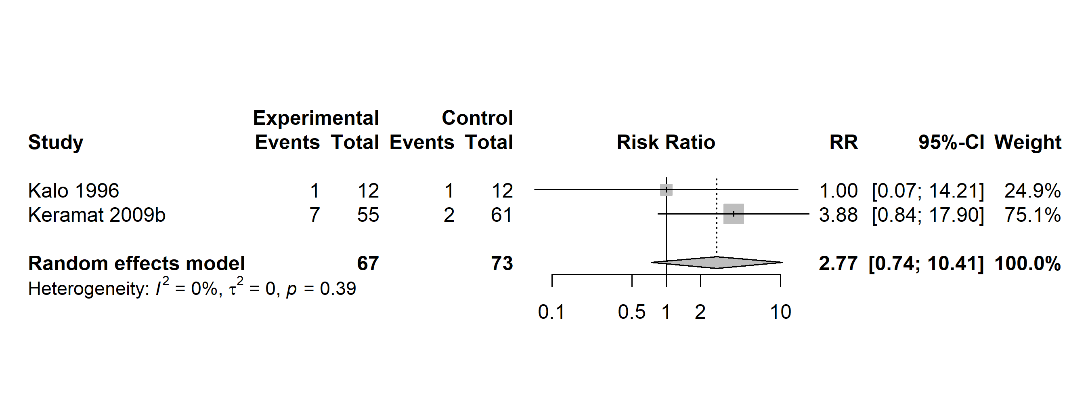


3.2 D+TMP/SMX


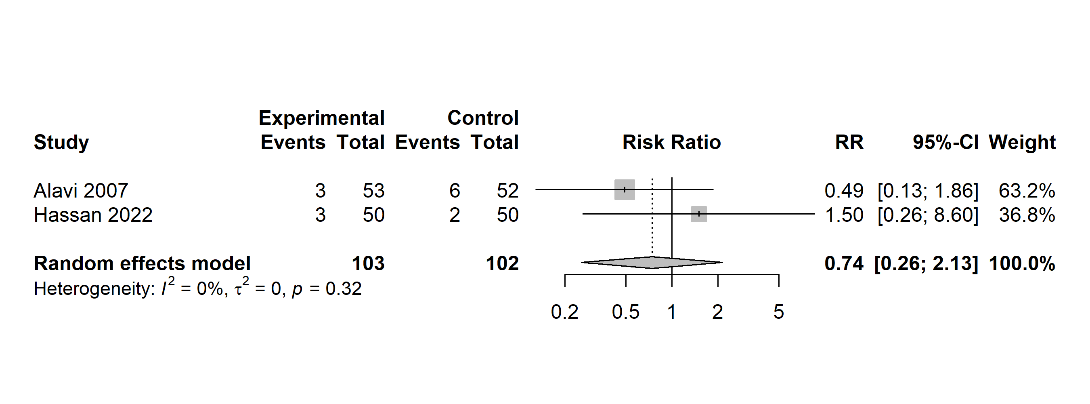


3.3 DS vs DR


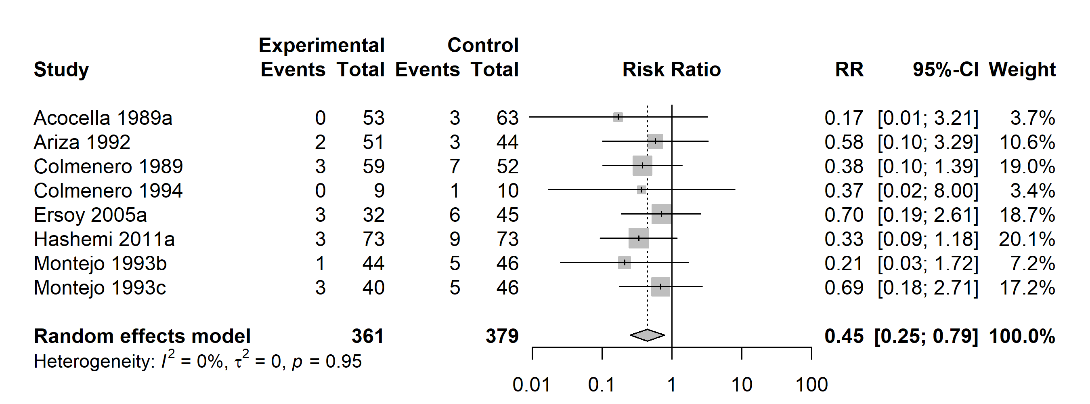


3.4 R+Quinolones vs DR


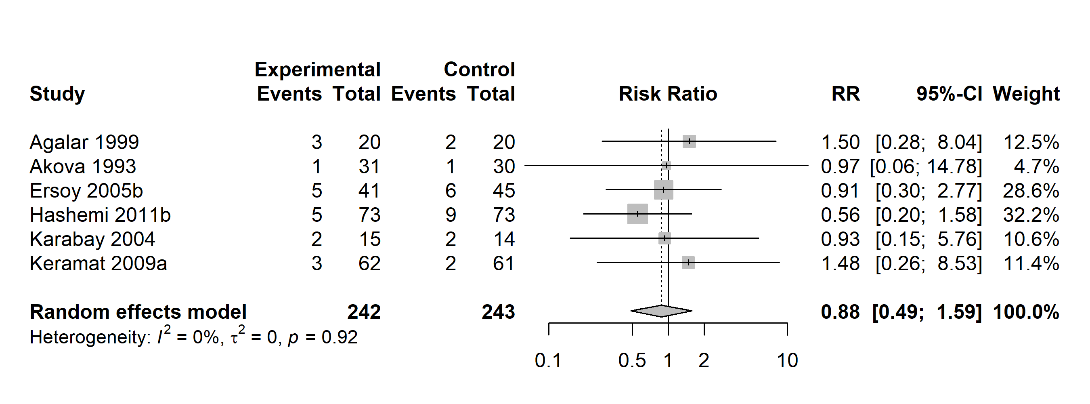


3.5 S+Tetracyclines vs DR


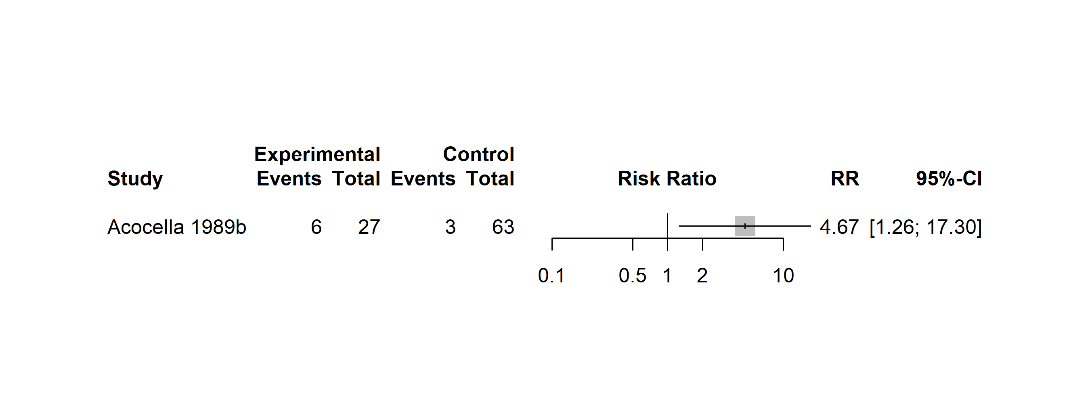


3.6 Single vs DR


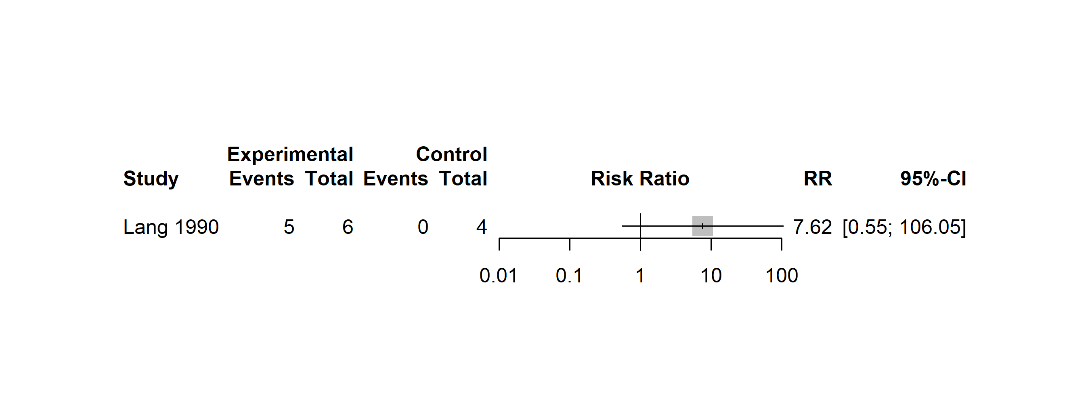


3.7 Triple vs DR


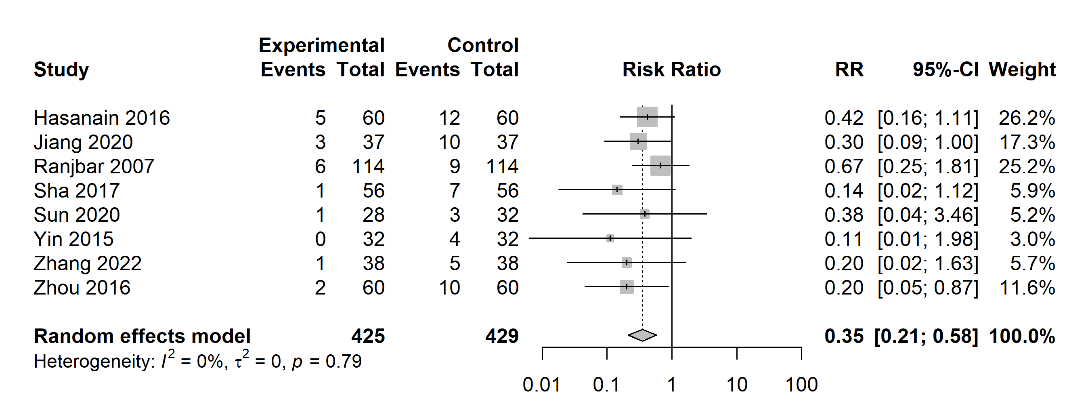


**4.** **Therapeutic failure**

4.1 D+Quinolones vs DR
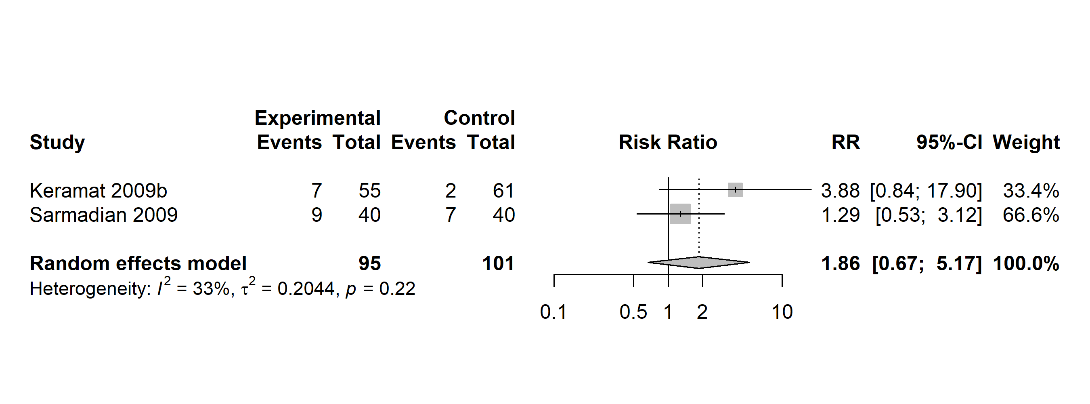


4.2 D+TMP/SMX


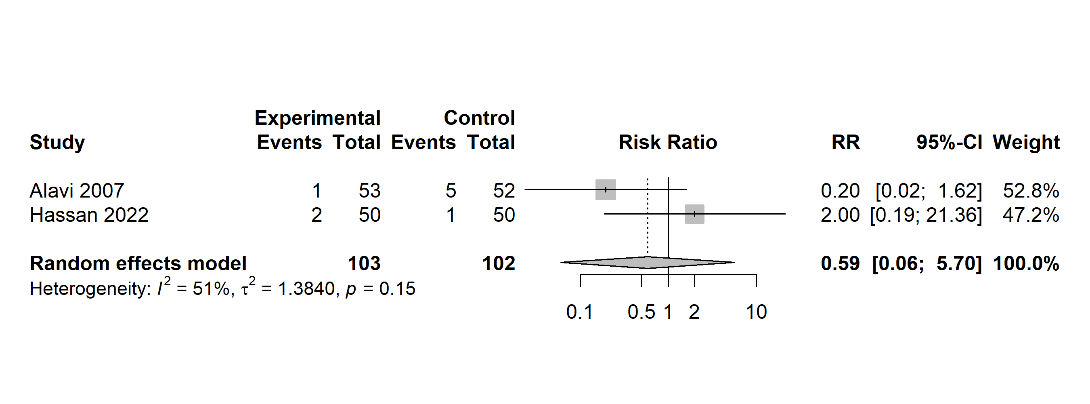


4.3 DS vs DR


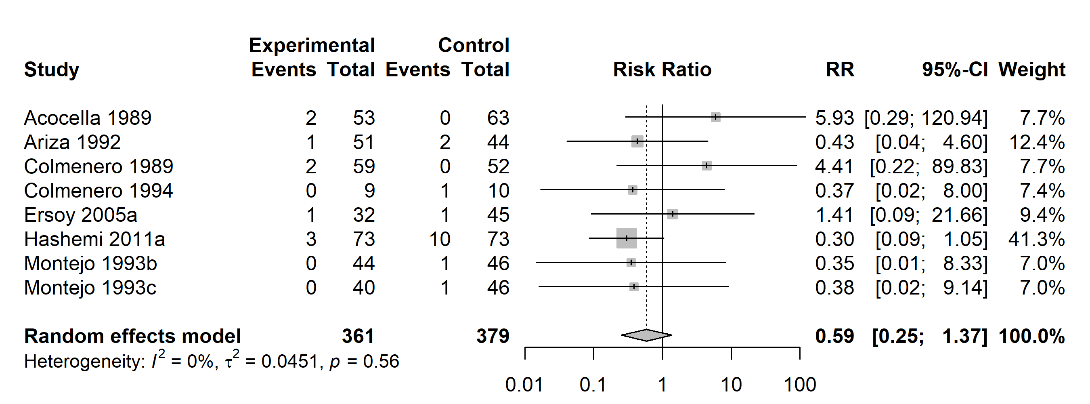


4.4 R+Quinolones vs DR


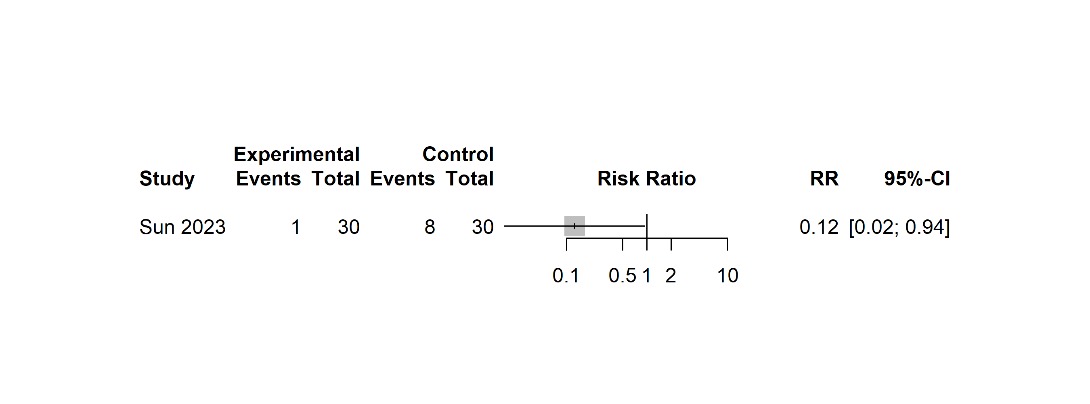


4.5 R+Tetracyclines vs DR


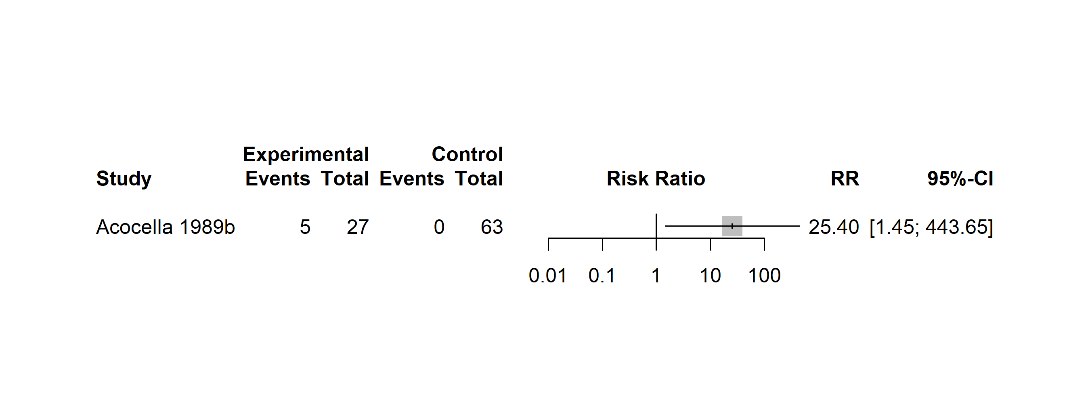


4.6 S+Tetracyclines vs DR


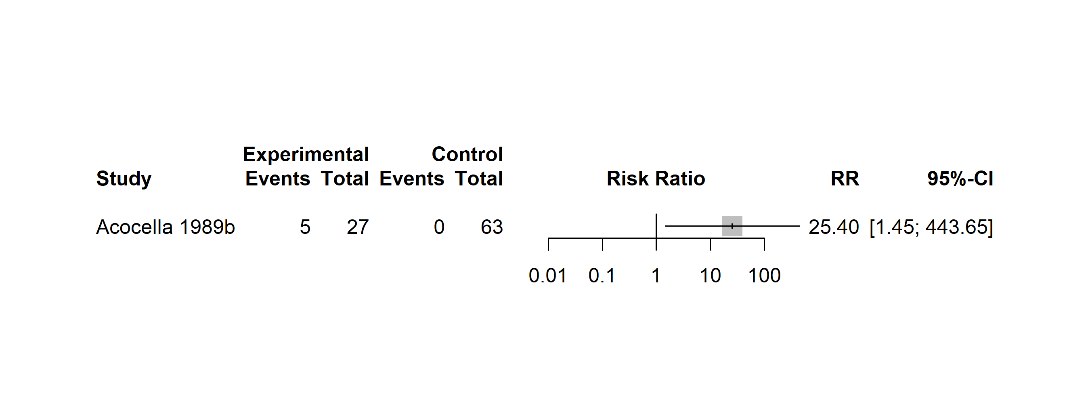


4.7 Single vs DR


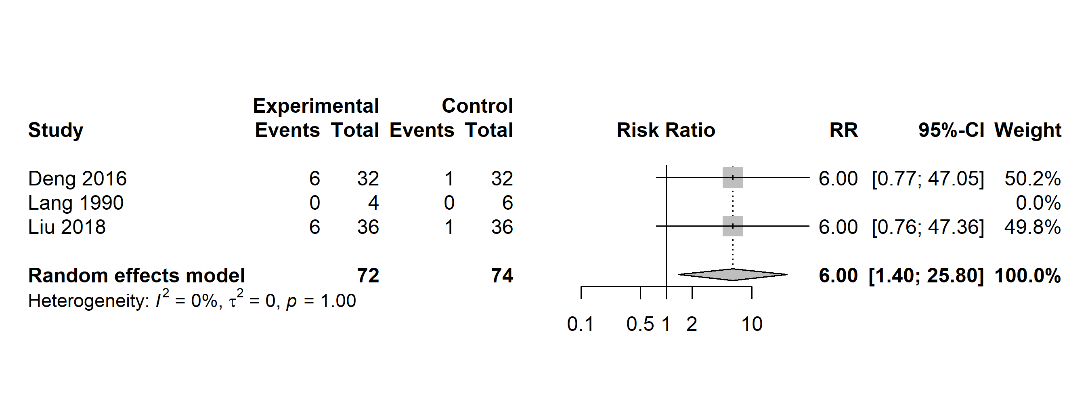


4.8 Triple vs DR


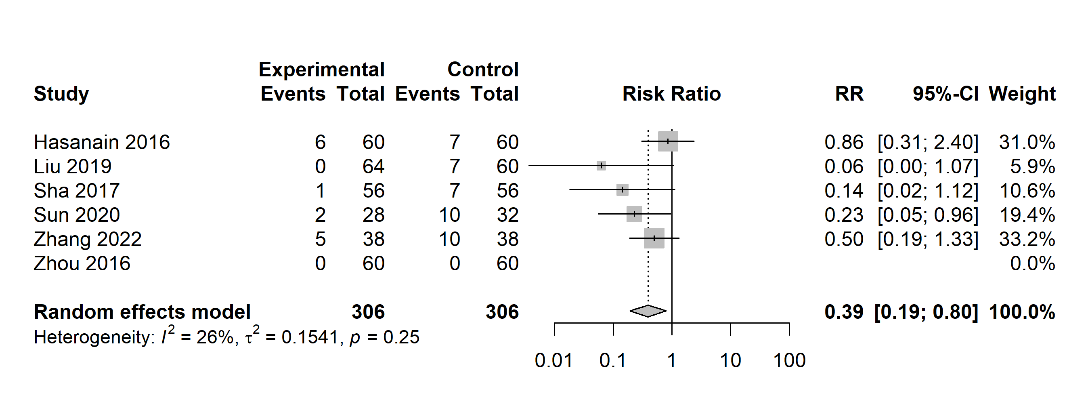

Supplement: S1 Fig — (DOCX) [file pntd.0012405.s015.docx]
