# Supplementary material for: Updated therapeutic options for human brucellosis: A systematic review and network meta-analysis of randomized controlled trials
Source: PLoS Negl Trop Dis. 2024 Aug 22;18(8):e0012405. doi: 10.1371/journal.pntd.0012405 (PMC11340890; doi:10.1371/journal.pntd.0012405)

**S2 Fig**. The network plots for secondary outcomes

**1. The network plot for relapse**


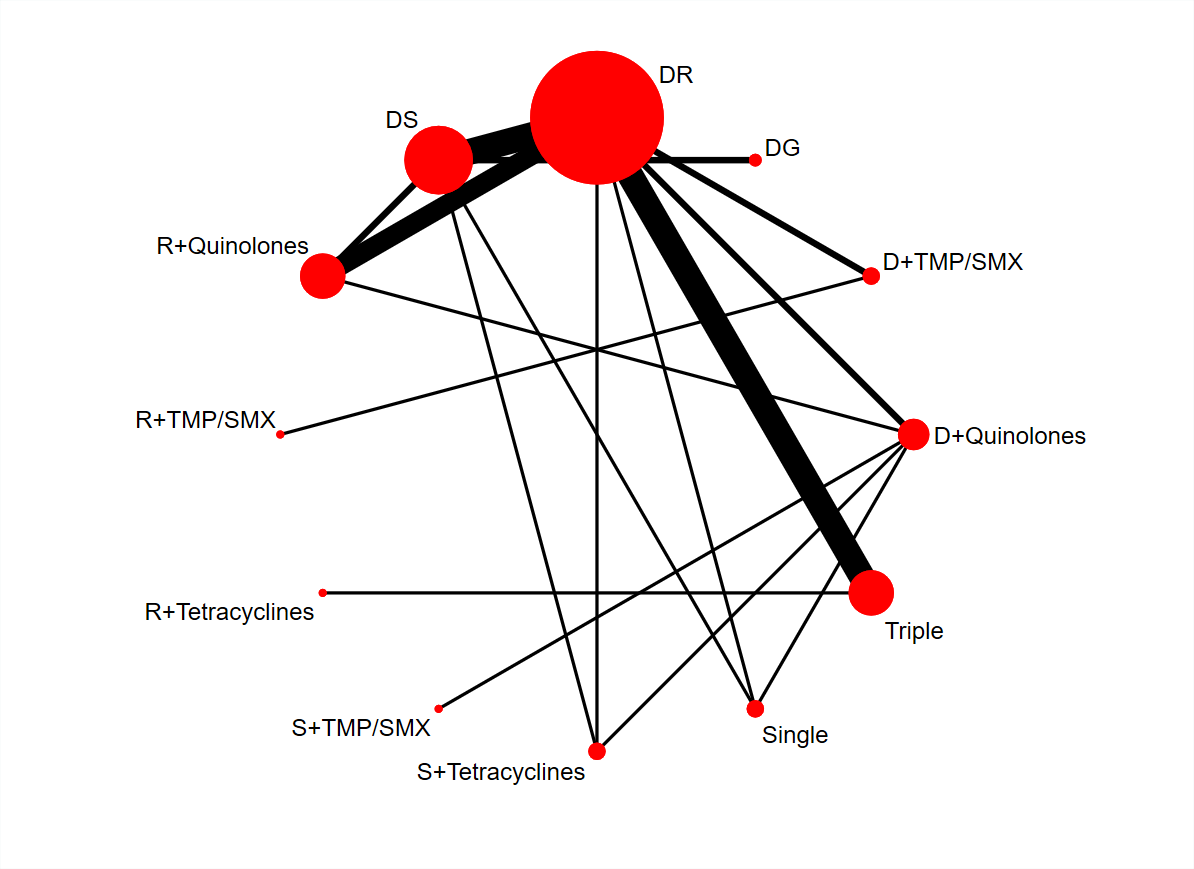


**2. The network plot for therapeutic failure**


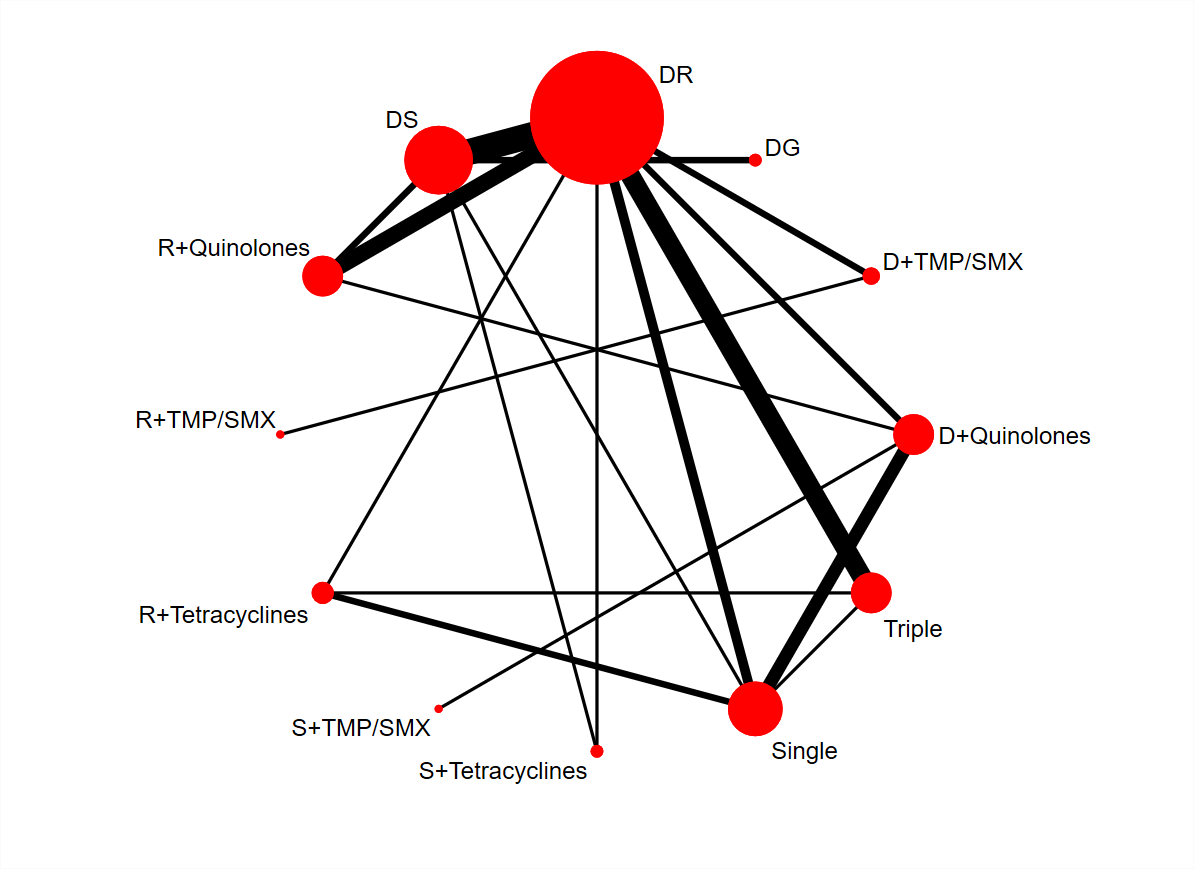

Supplement: S2 Fig — (DOCX) [file pntd.0012405.s016.docx]
