# Supplementary material for: Updated therapeutic options for human brucellosis: A systematic review and network meta-analysis of randomized controlled trials
Source: PLoS Negl Trop Dis. 2024 Aug 22;18(8):e0012405. doi: 10.1371/journal.pntd.0012405 (PMC11340890; doi:10.1371/journal.pntd.0012405)

**S3 Fig**. Sensitivity analyses

We conducted our sensitivity analysis by excluding studies with sample size less than 50, lack of follow-up time, and high risk of bias

**1. Results of overall failure**

1.1 Sample size less than 50


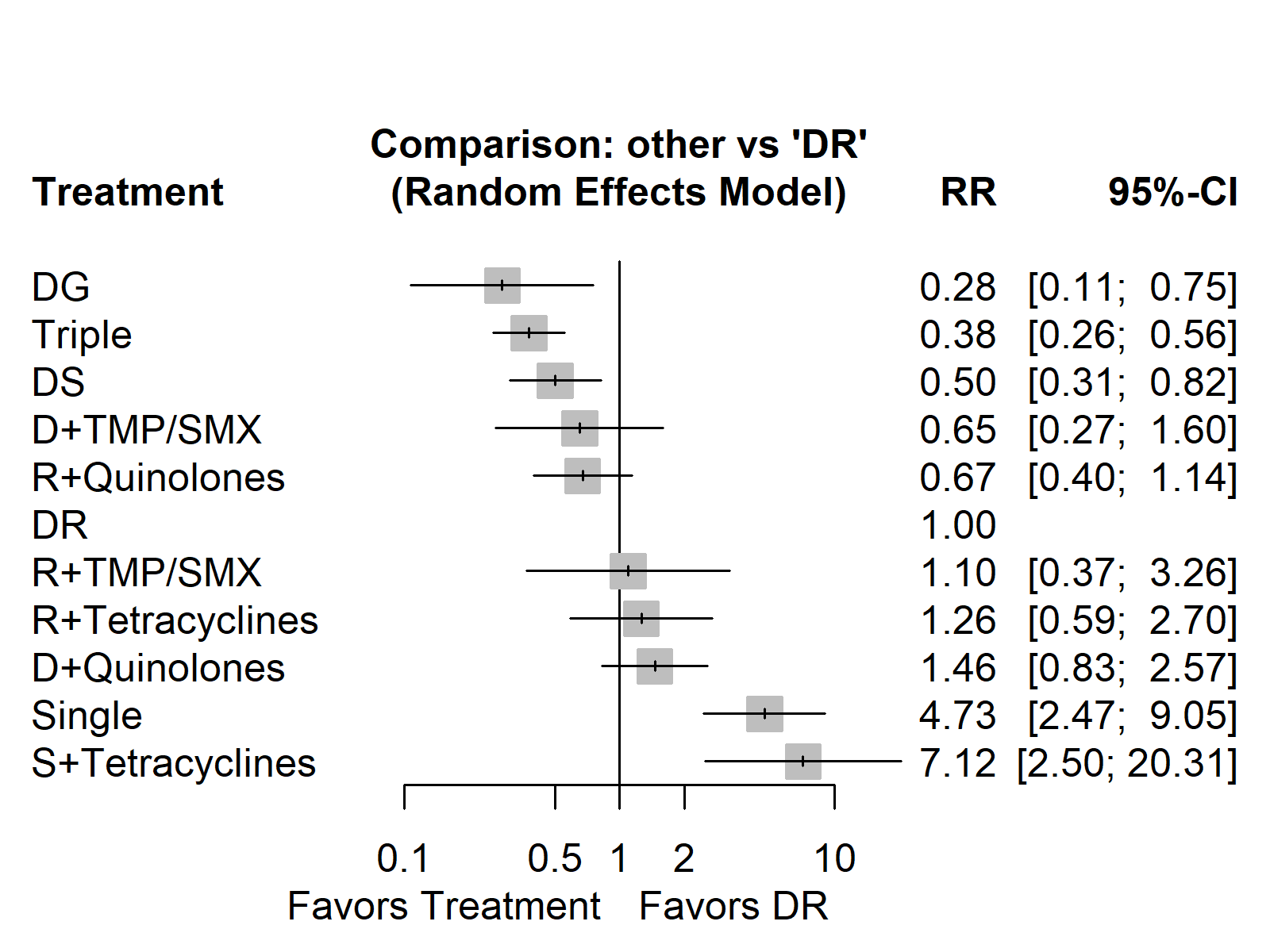


1.2 Lack of follow-up time


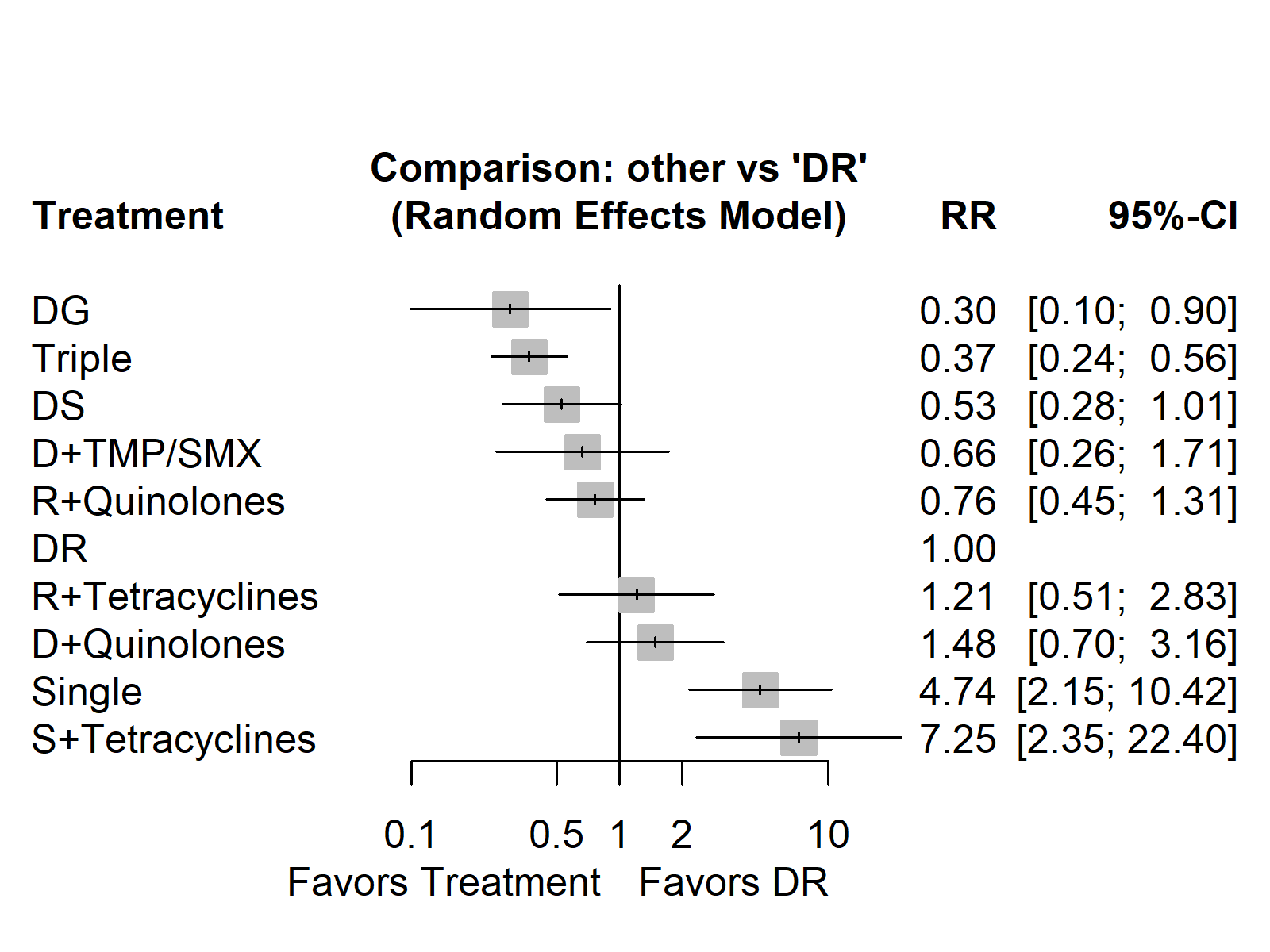


1.3 High risk of bias


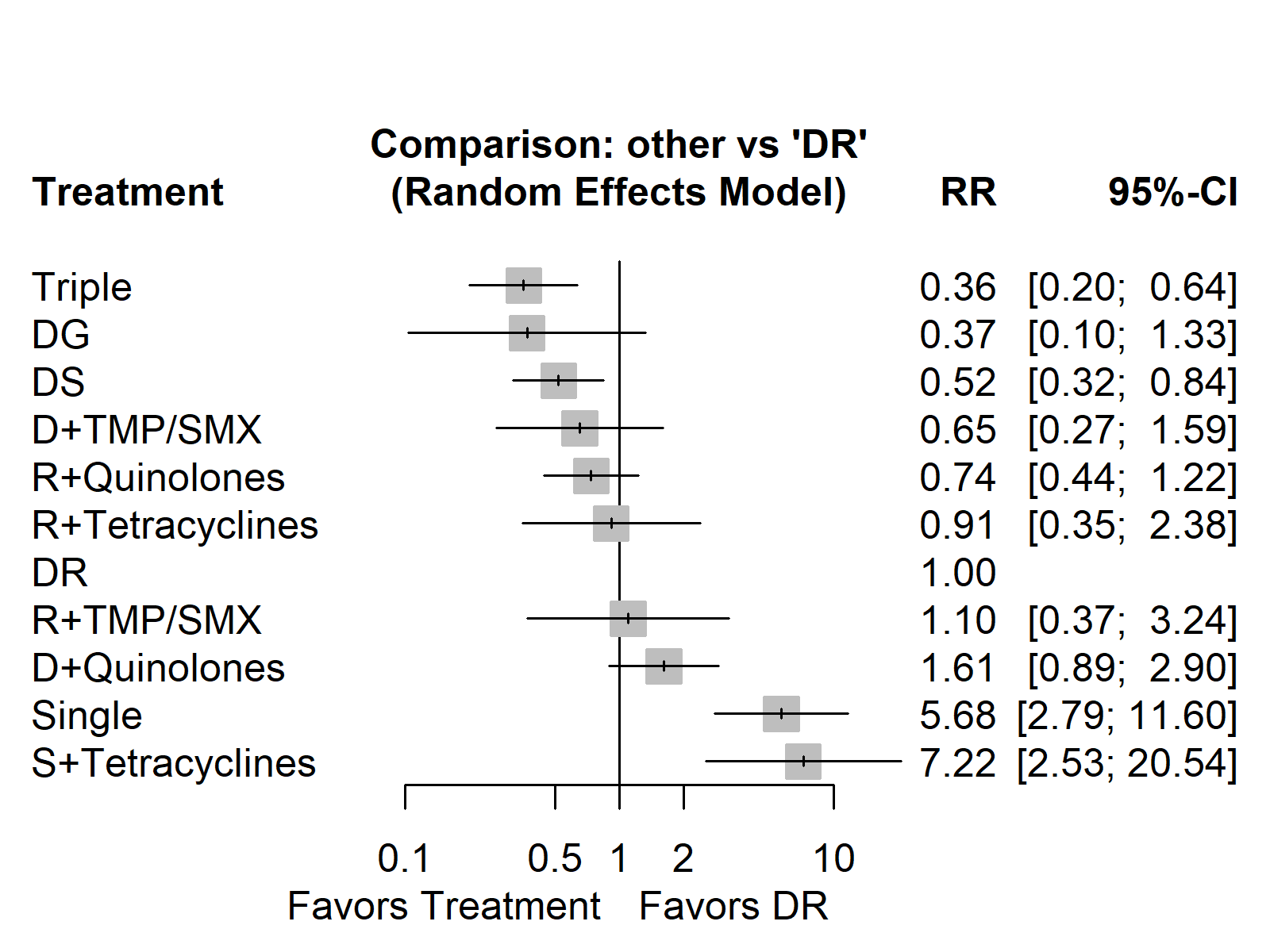


**2. Results of side effects**

2.1 Sample size less than 50


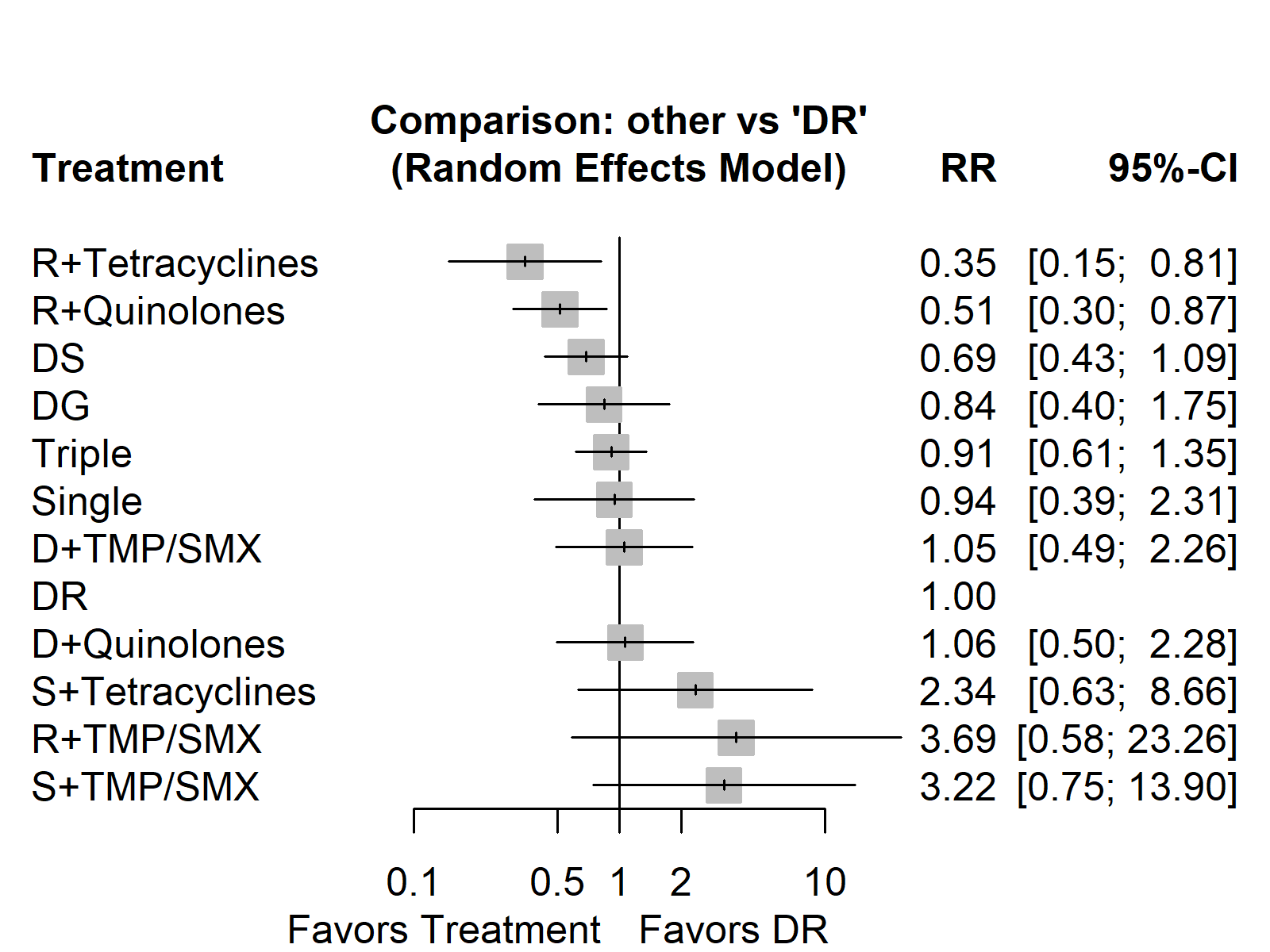


2.2 Lack of follow-up time


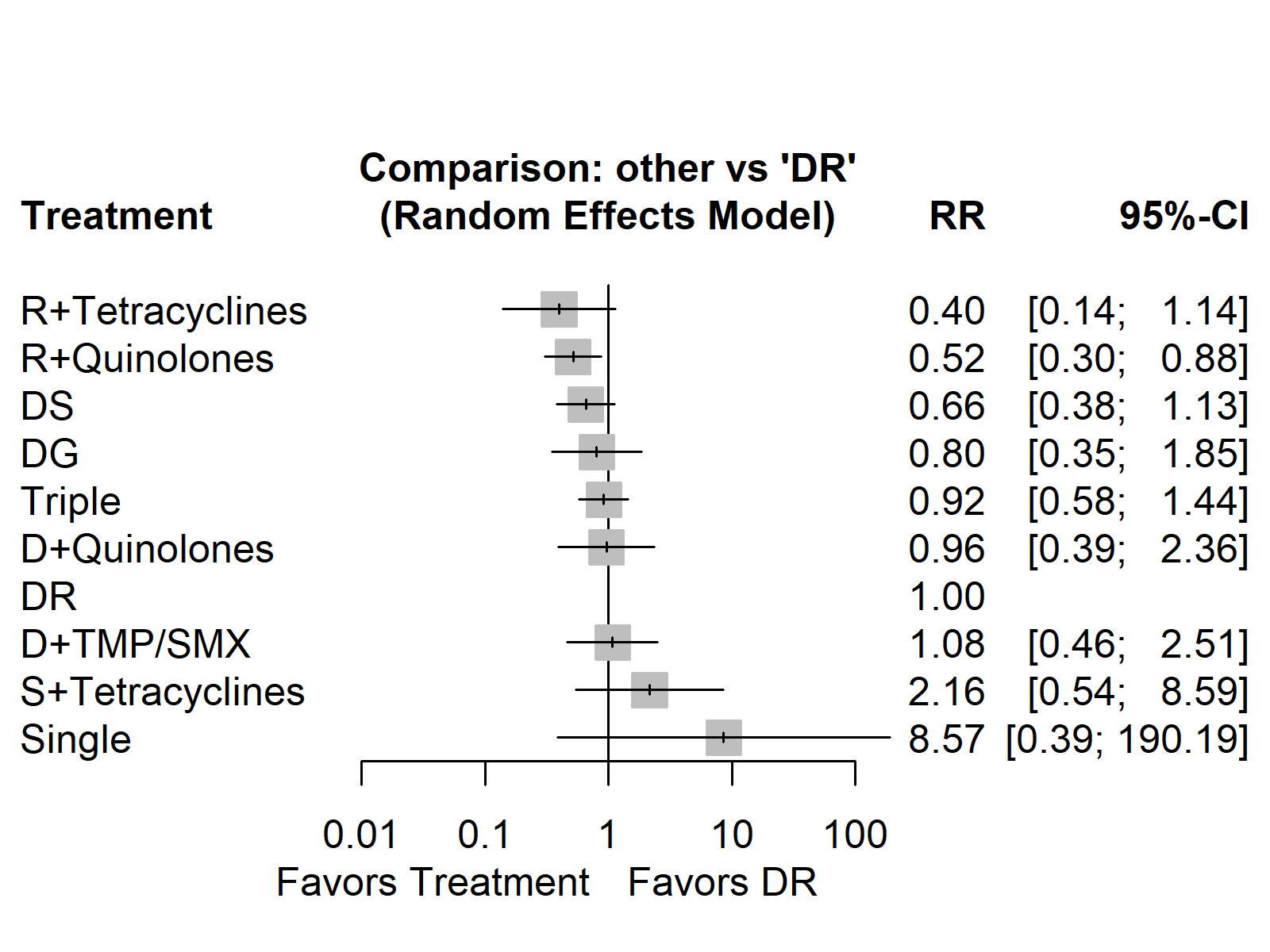


2.3 High risk of bias


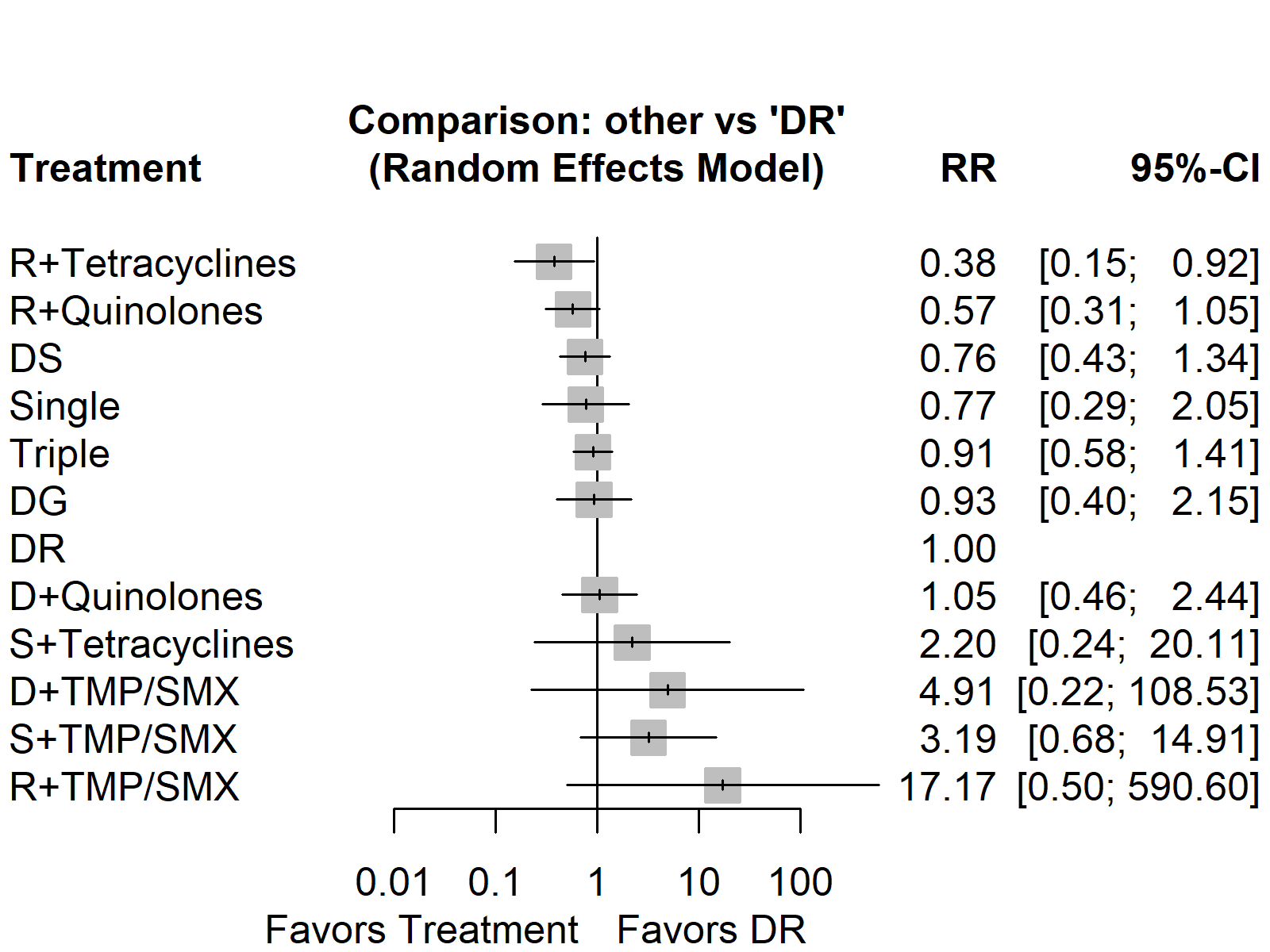

Supplement: S3 Fig — (DOCX) [file pntd.0012405.s017.docx]
