# Supplementary material for: Updated therapeutic options for human brucellosis: A systematic review and network meta-analysis of randomized controlled trials
Source: PLoS Negl Trop Dis. 2024 Aug 22;18(8):e0012405. doi: 10.1371/journal.pntd.0012405 (PMC11340890; doi:10.1371/journal.pntd.0012405)

**S4 Fig**. Publication bias assessment

Points of the same color represent the same comparison group

**1. The funnel plot of the network meta-analysis for overall failure (Egger’s test: t = 0.39, P = 0.696)**


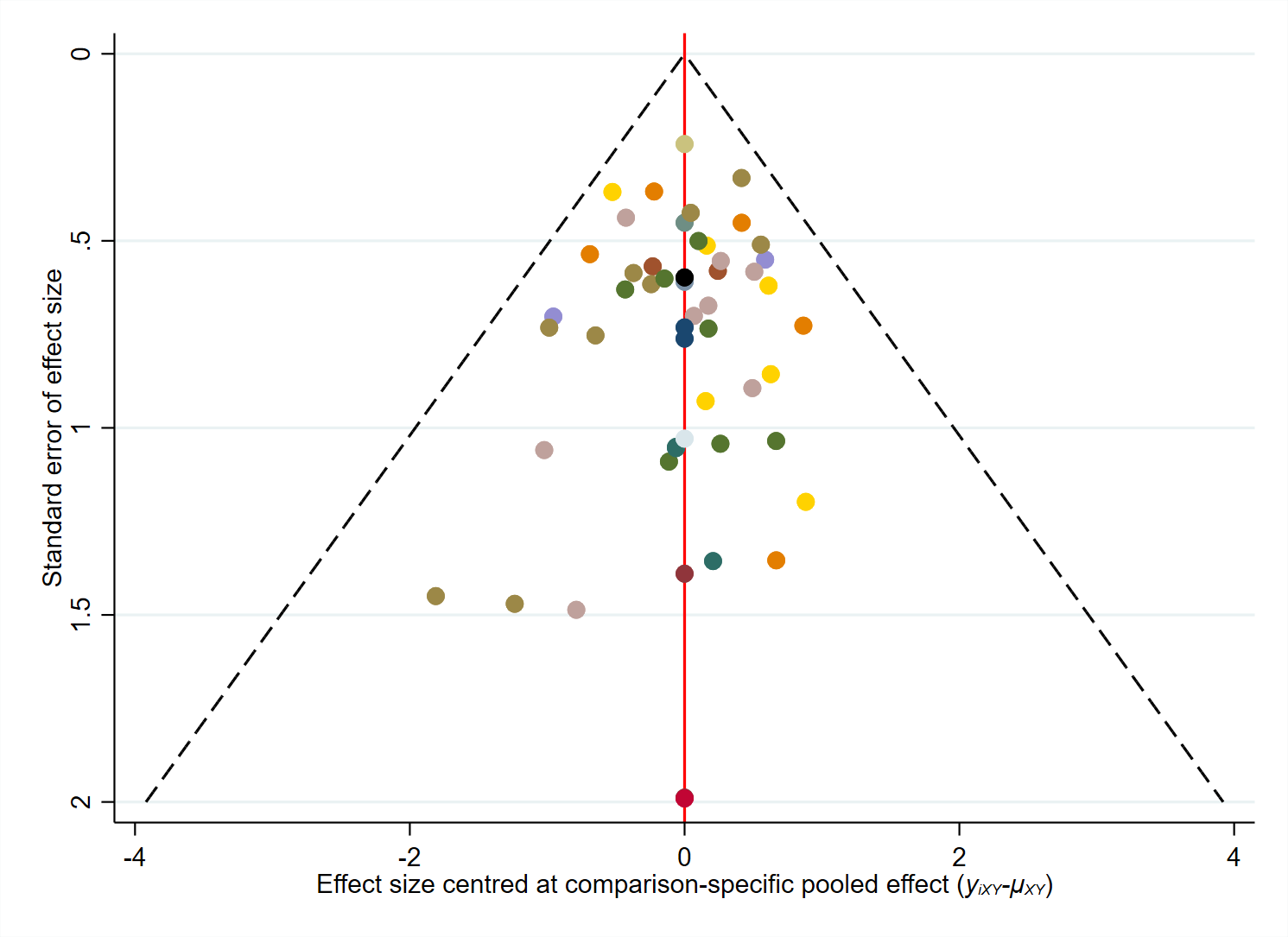


**2. The funnel plot of the network meta-analysis for side effects (Egger’s test: t = 1.93, P = 0.061)**

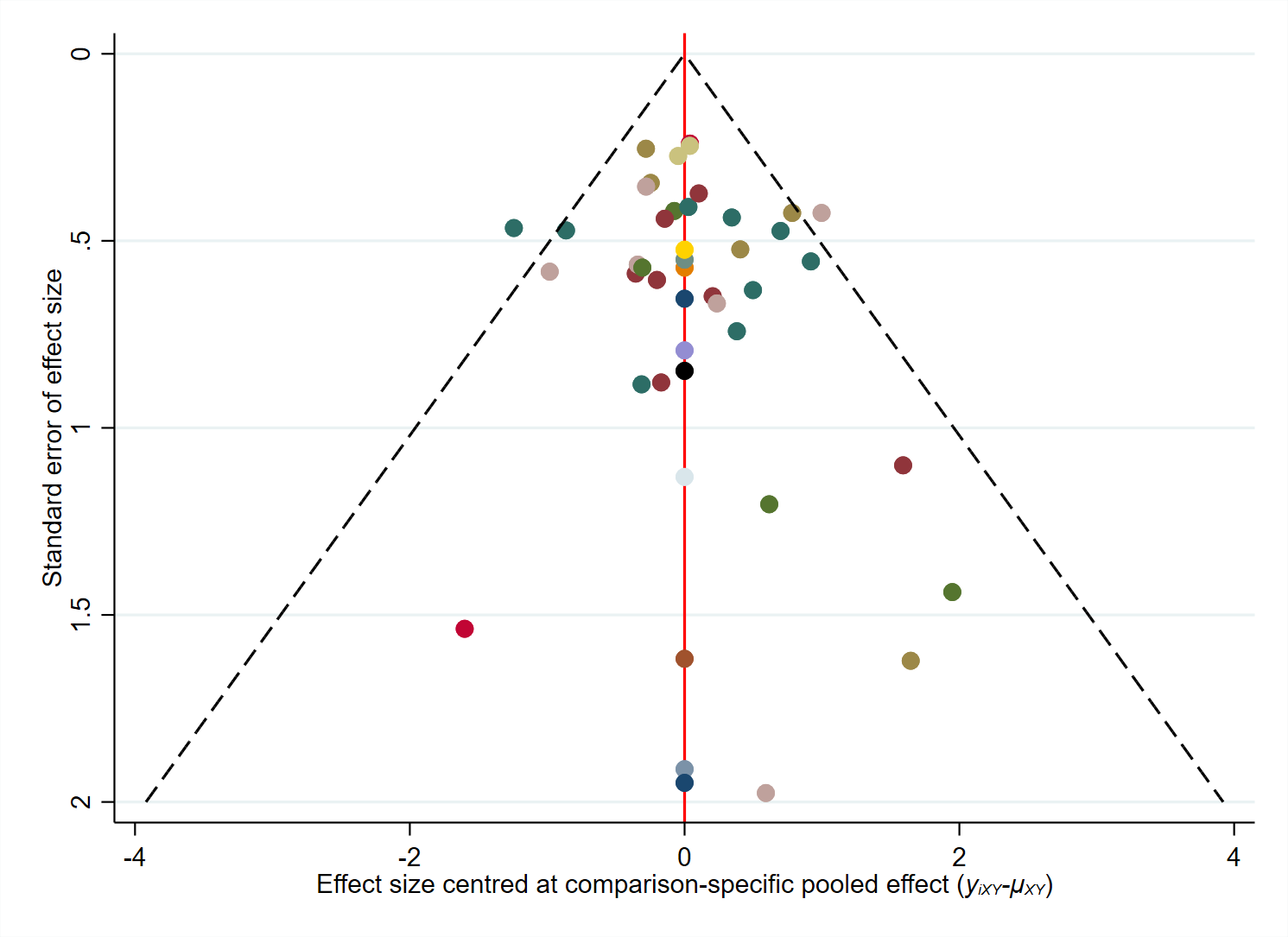


**3. The funnel plot of the network meta-analysis for relapse (Egger’s test: t = 0.18, P = 0.855)**


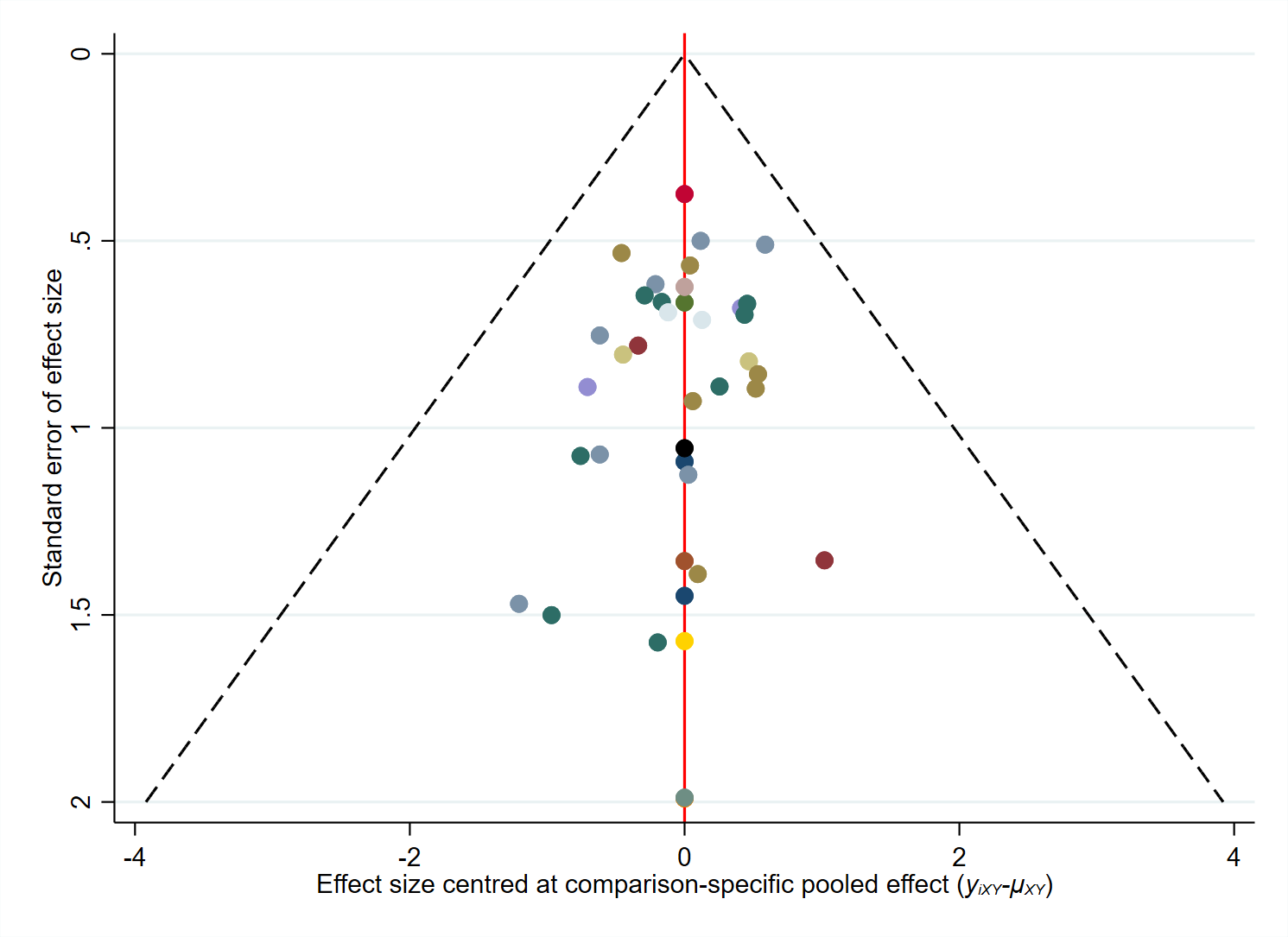


**4. The funnel plot of the network meta-analysis for therapeutic failure (Egger’s test: t = -0.12, P = 0.906)**


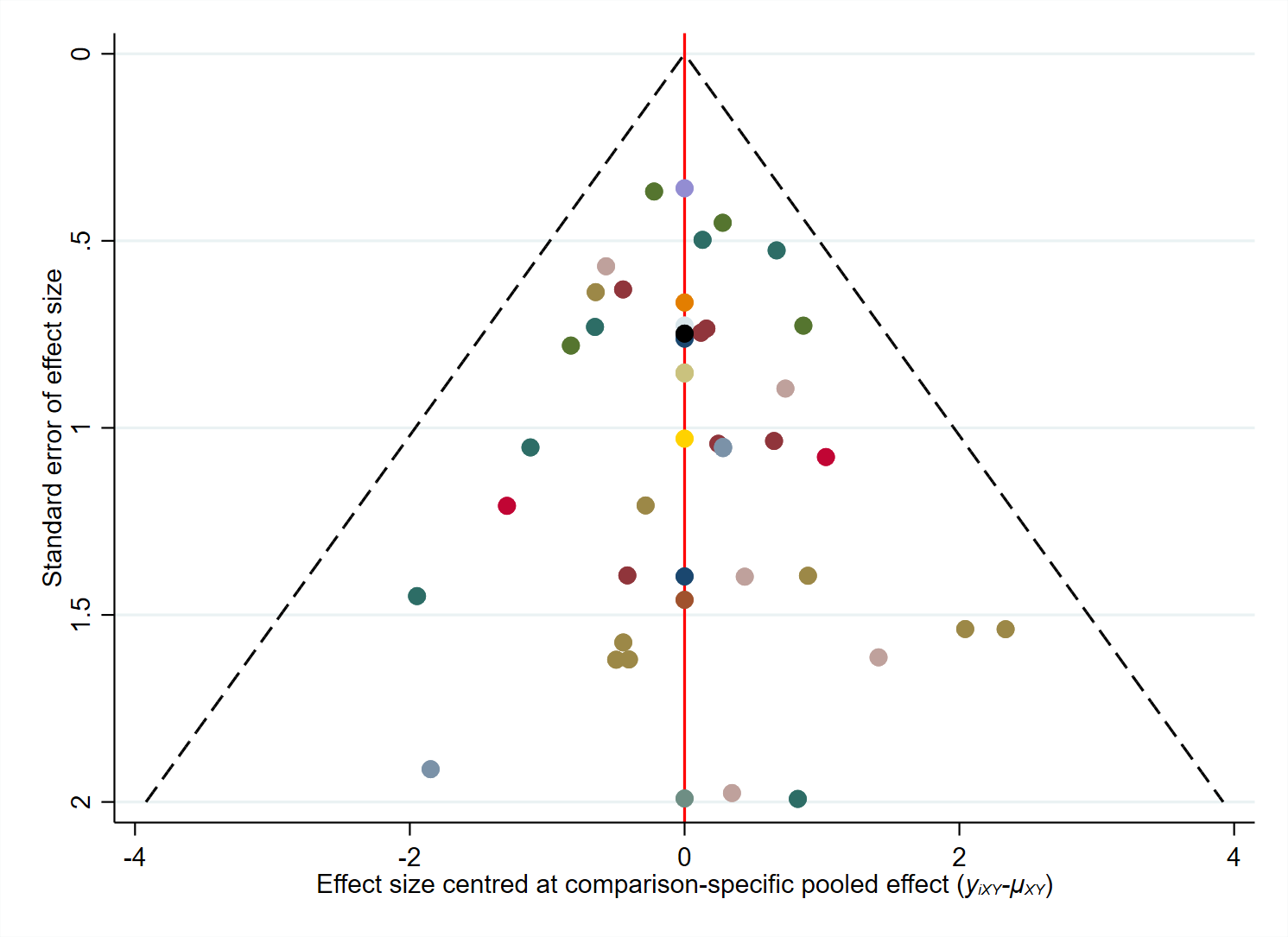

Supplement: S4 Fig — (DOCX) [file pntd.0012405.s018.docx]
